# On the nature of *fur *evolution: A phylogenetic approach in Actinobacteria

**DOI:** 10.1186/1471-2148-8-185

**Published:** 2008-06-25

**Authors:** Catarina L Santos, João Vieira, Fernando Tavares, David R Benson, Louis S Tisa, Alison M Berry, Pedro Moradas-Ferreira, Philippe Normand

**Affiliations:** 1 IBMC – Instituto de Biologia Molecular e Celular, Universidade do Porto, Rua do Campo Alegre, 823, 4150-180 Porto, Portugal; 2 Faculdade de Ciências da Universidade do Porto, Departamento de Botânica, Rua do Campo Alegre 1191, 4150-181 Porto, Portugal; 3 Department of Molecular and Cell Biology, University of Connecticut, Storrs, CT 06279, USA; 4 Department of Microbiology, University of New Hampshire, Durham, NH, 03824, USA; 5 Department of Plant Sciences, Mail Stop 1, PES Building, University of California, Davis, CA 95616, USA; 6 Instituto de Ciências Biomédicas Abel Salazar, Lg. Prof. Abel Salazar, 4099-003 Porto, Portugal; 7 UMR 5557 CNRS Ecologie Microbienne, IFR41 Bio-Environnement et Santé, Université Lyon 1, 43 Bd du 11 novembre 1918, 69622 Villeurbanne Cedex, France

## Abstract

**Background:**

An understanding of the evolution of global transcription regulators is essential for comprehending the complex networks of cellular metabolism that have developed among related organisms. The *fur *gene encodes one of those regulators – the ferric uptake regulator Fur – widely distributed among bacteria and known to regulate different genes committed to varied metabolic pathways. On the other hand, members of the Actinobacteria comprise an ecologically diverse group of bacteria able to inhabit various natural environments, and for which relatively little is currently understood concerning transcriptional regulation.

**Results:**

BLAST analyses revealed the presence of more than one *fur *homologue in most members of the Actinobacteria whose genomes have been fully sequenced. We propose a model to explain the evolutionary history of *fur *within this well-known bacterial phylum: the postulated scenario includes one duplication event from a primitive regulator, which probably had a broad range of co-factors and DNA-binding sites. This duplication predated the appearance of the last common ancestor of the Actinobacteria, while six other duplications occurred later within specific groups of organisms, particularly in two genera: *Frankia *and *Streptomyces*. The resulting paralogues maintained main biochemical properties, but became specialised for regulating specific functions, coordinating different metal ions and binding to unique DNA sequences. The presence of syntenic regions surrounding the different *fur *orthologues supports the proposed model, as do the evolutionary distances and topology of phylogenetic trees built using both Neighbor-Joining and Maximum-Likelihood methods.

**Conclusion:**

The proposed *fur *evolutionary model, which includes one general duplication and two in-genus duplications followed by divergence and specialization, explains the presence and diversity of *fur *genes within the Actinobacteria. Although a few rare horizontal gene transfer events have been reported, the model is consistent with the view of gene duplication as a main force of microbial genomes evolution. The parallel study of Fur phylogeny across diverse organisms offers a solid base to guide functional studies and allows the comparison between response mechanisms in relation with the surrounding environment. The survey of regulators among related genomes provides a relevant tool for understanding the evolution of one of the first lines of cellular adaptability, control of DNA transcription.

## Background

Fur proteins form a ubiquitous family of metal-responsive transcription factors known to regulate the transcription of several different genes in many diverse bacterial lineages. Upon binding to a metal ion, a conformational change is induced in the Fur regulator that promotes interaction with a cognate DNA sequence, typically known as a Fur or iron box [1]. Initially, Fe (II) was thought to be the only metal able to play this role. In fact, Fur was initially characterised as being an iron-responsive regulator of ferric iron uptake systems in *Escherichia coli *[2,3], hence its name. However, several studies have shown that Fur can bind other metals – besides iron – as co-factors, and thus the range of known regulated genes became broader than what was initially thought. Fur will bind Fe (II) and regulate iron homeostasis in several organisms [2,4-8]. However, in addition to iron, different Fur homologues specifically require other divalent metals, including Zn^2+^, Ni^2+^, Mn^2+ ^or Co^2+^, in order to bind to their cognate promoter targets [9-11]. These transition metal ions are considered fundamental for bacterial growth, given that they perform various essential functions in cellular metabolism. However, most of them are toxic at elevated levels. Therefore, a strict balance between their uptake and efflux, effected by metalloregulators like Fur, is essential for homeostasis [12-15]. Excess amounts of these metal ions elicit a number of stress conditions inside the cells, particularly oxidative stress [16]. Accordingly, some Fur-like proteins, through sensing the availability of their specific metal co-factor, are sensitive to the redox status of the cell, establishing a relationship between these regulators and the oxidative stress response [17-20].

Recent publications support a major role for Fur in the regulation of various environmental conditions including acid shock response, detoxification of oxygen radicals, production of toxins and virulence factors, and several other metabolic functions ([1] and references therein). Particularly during pathogenic infections, iron and possibly other metals become generally unavailable. Therefore, bacterial metalloregulatory proteins including Fur are often crucial in pathogenesis processes. These observations have led to a growing recognition of the importance of Fur as a global transcriptional factor.

Among the Fur-like proteins, those responding to oxidative stress by regulating a downstream catalase-peroxidase have been the most studied in the Actinobacteria [19,21-24]. However, despite detailed knowledge on catalase-peroxidase regulation and the variety of functional and structural studies on numerous microorganisms, very little is known about the origin and molecular evolution of *fur*. The increasingly recognised importance of Fur as a virulence factor [24-26] and as a potential target for novel antibiotics [10,25] would be better addressed with a deeper knowledge on its evolutionary history. The enormous diversity of Fur in terms of both required co-factors and regulated genes has led to several efforts to organise the family [9,27].

The phylum *Actinobacteria *is comprised of Gram-positive bacteria with an overall high Mol% G+C content. The primary habitat for many of these bacteria is the soil, where they degrade organic compounds and play an important role in mineralisation. The lineage also contains important secondary metabolite-producers and several important pathogens and symbionts. The latter groups include the mycobacterial agents of tuberculosis and leprosy and the nitrogen-fixing plant microsymbionts *Frankia *spp., among other ecologically and economically important microorganisms [28]. Actinobacteria also inhabit aquatic systems, while others are associated with extreme environments such as acidic thermal springs [29], Antarctic regolith [30] or gamma [31] and UV irradiated biotopes [32]. The ability to inhabit these different environments probably selects for the capacity to sense and cope with a wide range of metals, for which regulators of the Fur family are important. It is this diversity of habitats and lifestyles that makes Actinobacteria such an excellent subject for an evolutionary study of a global regulator like Fur.

The diversity of genes encoding regulators in a genome defines an organism's ability to adjust to the surrounding environment. Therefore, towards a parallel vision of the different organisms and their conserved response mechanisms, we have undertaken a phylogenetic approach to the Fur family using Actinobacteria as the model clade, intending to extend and complement previous functional and structural studies in an evolutionary perspective. We have analysed the factors that shaped Fur regulatory functions in different bacteria in order to create a bridge between origin and cellular role. An hypothesis that clarifies the presence and diversity of the Fur homologues in Actinobacteria is presented, leading to a stable protein family division based on functionality and phylogeny. Furthermore, a relationship is established between each organism's ecological niche, genome size, and number of Fur homologues.

## Results and Discussion

### Overview of Fur homologues

To describe the phylogenetic history of the Fur regulators, and knowing by previous reports that organisms may have more than one Fur, only completely sequenced actinobacterial genomes present in the NCBI (National Centre for Biotechnology Information) database (March/2007) were used. Since regulatory proteins are small and not highly conserved, the Fur homologues included in this study were chosen based not only on sequence similarity/identity values, but also on the presence of specific residues necessary for the *in vivo *regulatory activity of Fur. Functional studies on *Streptomyces reticuli *FurS have shown the importance of five key residues: C96 and C99 are involved in reversible S-S bond formation, Y59 is required for DNA binding and C96, H92 and H93 are implicated in zinc coordination [20]. These residues were conserved not only in the closely related mycobacterial FurA, but also in the more distantly related *Escherichia coli *Fur, making them good indicators to validate the occurrence of a Fur homologue.

An initial BlastP screening against each of the 36 actinobacterial genomes yielded 82 putative homologues. These sequences were aligned with *S. reticuli *FurS to check for the presence of the above mentioned key residues [see Additional File 1]. Two putative homologues were eliminated at this stage, the Mjls_1895 from *Mycobacterium *sp. JLS, that lacks all the key residues, and the Rxyl_1224 from *Rubrobacter xylanophilus *DSM 9941, that lacks one of the histidines. Conversely, Lxx02790, from *Leifsonia xyli *subsp. xyli str. CTCB07, has an H instead of the Y59. Since histidines and tyrosines are both polar and have similar properties, this homologue was nevertheless retained. Also retained were a number of homologues that presented the two histidines corresponding to H92 and H93 in the form HXH. The remaining 80 sequences were distributed as listed in Table 1: five genomes had four Fur homologues, ten genomes had three, eleven genomes had two, eight genomes had one, and finally two genomes did not present any Fur homologue.

**Table 1:** Ecological and genomic context of the *fur *homologues

species and strains designation	RefSeq	genome size (Mbps)	%GC	Fur homologues	group	*fur *%GC	species habitat
***Acidothermus cellulolyticus *11B**	NC_008578	2,4	67	Acel_2095	A	67,1	aquatic; aerobic; thermophilic
				Acel_0061	E	64,9	
				Acel_2085	B	65,5	
***Arthrobacter aurescens *TC1**	NC_008711	4,6	62	AAur_3058	A	60,6	terrestrial – can transform heavy metals into less toxic forms; aerobic; mesophilic
				AAur_2630	B	61,1	
***Arthrobacter *sp. FB24**	NC_008541	4,7	66	Arth_3077	A	65,8	terrestrial – high degree of tolerance to chromium and other metals, may be radiation resistant and is a hydrocarbon degrader; mesophilic
				Arth_2638	B	68,2	
***Bifidobacterium adolescentis *ATCC 15703**	NC_008618	2,1	59	BAD_0517	B	62,2	host-associated – normal inhabitant of the healthy human gut; anaerobic; mesophilic
***Bifidobacterium longum *NCC2705**	NC_004307	2,3	60	BL1128	B	61,2	host-associated – normal component of gut flora; anaerobic; mesophilic
***Corynebacterium diphtheriae *NCTC 13129**	NC_002935	2,5	54	DIP1710	B	51,5	multiple – human pathogen, causative agent of diphtheria; aerobic; mesophilic
***Corynebacterium efficiens *YS-314**	NC_004369	3,1	63	CE2180	B	63,1	multiple; facultative aerobic; mesophilic
***Corynebacterium glutamicum *ATCC 13032**	NC_006958	3,3	54	NCgl2200	B	53,3	multiple; facultative aerobic; mesophilic
***Corynebacterium jeikeium *K411**	NC_007164	2,5	61	jk0612	B	62,5	multiple – member of the human skin flora, opportunistic pathogen; facultative aerobic; mesophilic
***Frankia alni *ACN14a**	NC_008278	7,5	73	FRAAL3168	A	72,0	free-living or in symbiosis with plants roots; aerobic; mesophilic
				FRAAL0074	B	72,0	
				FRAAL5117	C	71,4	
				FRAAL2798	E	76,4	
***Frankia *sp. CcI3**	NC_007777	5,4	70	Francci3_3112	C	70,3	free-living or in symbiosis with plants roots; aerobic; mesophilic
				Francci3_2661	E	73,9	
				Francci3_0061	B	71,8	
***Frankia *sp. EAN1pec**	NZ_AAII00000000	9,0	71	Franean1_1532	A	70,3	free-living or in symbiosis with plants roots; aerobic; mesophilic
				Franean1_1806	C	73,2	
				Franean1_6149	E	72,4	
				Franean1_7263	B	74,1	
***Leifsonia xyli *subsp. xyli str. CTCB07**	NC_006087	2,6	68	Lxx02790	A	70	host-associated; plant pathogen; aerobic; mesophilic
				Lxx25010	B	69,1	
***Mycobacterium avium *104**	NC_008595	5,5	69	MAV_2752	A	68,9	host-associated – mammal pathogen; aerobic; mesophilic
				MAV_2036	B	68,4	
***Mycobacterium avium *subsp. paratuberculosis K-10**	NC_002944	4,8	69	MAP1669c	A	68,7	multiple – mammal pathogen, causative agent of Johne's disease or paratuberculosis; aerobic; mesophilic
				MAP2139	B	67,9	
***Mycobacterium bovis *AF2122/97**	NC_002945	4,3	66	Mb1944c	A	62,3	host-associated – mammal pathogen, causative agent of classic bovine tuberculosis; aerobic; mesophilic
				Mb2380	B	62,8	
***Mycobacterium bovis *BCG str. Pasteur**	NC_008769	4,4	66	BCG_1948c	A	62,0	host-associated – mammal pathogen, causative agent of classic bovine tuberculosis; aerobic; mesophilic
				BCG_2373	B	62,8	
***Mycobacterium leprae *TN**	NC_002677	3,3	58	ML0824	B	59,1	host-associated – human pathogen, causative agent of leprosy; aerobic; mesophilic
***Mycobacterium smegmatis *str. MC2 155**	NC_008596	7,0	67	MSMEG_6253	A	67,3	host-associated – human pathogen; aerobic; mesophilic
				MSMEG_6383	A	70,6	
				MSMEG_3460	A	64,9	
				MSMEG_4487	B	70,6	
***Mycobacterium *sp. JLS**	NC_009077	6,0	68	Mjls_2712	A	68,5	multiple; mesophilic
				Mjls_5253	A	72,7	
				Mjls_3458	B	71,1	
***Mycobacterium *sp. KMS**	NC_008705	5,7	68	Mkms_2726	A	68,8	multiple; mesophilic
				Mkms_4974	A	72,2	
				Mkms_3510	B	71,5	
***Mycobacterium *sp. MCS**	NC_008146	5,7	69	Mmcs_2681	A	66,5	multiple; mesophilic
				Mmcs_4885	A	72,2	
				Mmcs_3447	B	71,5	
***Mycobacterium tuberculosis *CDC1551**	NC_002755	4,4	66	MT1960	A	62,3	host-associated – human pathogen, causative agent of tuberculosis; aerobic; mesophilic
				MT2428	B	62,6	
***Mycobacterium tuberculosis *H37Rv**	NC_000962	4.4	66	Rv1909c	A	62,3	host-associated – human pathogen, causative agent of tuberculosis; aerobic; mesophilic
				Rv2359	B	62,6	
***Mycobacterium ulcerans *Agy99**	NC_008611	5,6	66	MUL_2189	A	63,8	host-associated – human pathogen, causative agent of Buruli or Bairnsdale ulcer; aerobic; mesophilic
				MUL_3612	B	65,2	
***Mycobacterium vanbaalenii *PYR-1**	NC_008726	6,5	68	Mvan_2983	A	69,3	multiple; aerobic; mesophilic
				Mvan_3209	A	65,8	
				Mvan_3820	B	67,9	
***Nocardia farcinica *IFM 10152**	NC_006361	6,0	71	nfa3250	A	75,0	multiple – opportunistic pathogens in a broad range of species; aerobic; mesophilic
				nfa29490	A	68,5	
				nfa14570	B	70,2	
***Nocardioides *sp. JS614**	NC_008699	5,0	72	Noca_0839	A	74,5	terrestrial; aerobic; mesophilic
				Noca_0874	A	75,1	
				Noca_4251	E	70,2	
				Noca_1934	B	74,0	
***Propionibacterium acnes*****KPA171202**	NC_006085	2,6	60	PPA0948	B	57,0	host-associated – opportunistic human pathongen, causative agent of acne; anaerobic; mesophilic
***Rhodococcus *sp. RHA1**	NC_008268	7,8	68	RHA1_ro04308	A	67,5	terrestrial; aerobic; mesophilic
				RHA1_ro05274	A	66,2	
				RHA1_ro01222	B	67,4	
***Rubrobacter xylanophilus *DSM 9941**	NC_008148	3,2	71	Rxyl_1228	-	67,6	specialized – exhibits high tolerance to radiation; aerobic; thermophilic
				Rxyl_1140	-	68,0	
				Rxyl_1144	-	75,6	
***Streptomyces avermitilis *MA-4680**	NC_003155	9,0	71	SAV3053	D	71,9	multiple – mainly found in the soil; aerobic; mesophilic
				SAV4029	E	64,8	
				SAV5631	B	70,7	
***Streptomyces coelicolor *A3(2)**	NC_003888	8,7	72	SCO0561	A	74,3	multiple – mainly found in the soil; aerobic; mesophilic
				SCO5206	D	72,2	
				SCO4180	E	66,4	
				SCO2508	B	72,6	
***Thermobifida fusca *YX**	NC_007333	3,6	68	Tfu_0145 Tfu_0856	A B	68,8 64,6	multiple – mainly found on self-heated organic materials, its spores are known to cause allergic respiratory diseases; aerobic; thermophilic
***Tropheryma whipplei *TW08/27**	NC_004551	0,9	46	-		-	Host-associated – human pathogen, causative agent of Whipple's disease; aerobic; mesophilic
***Tropheryma whipplei *str. Twist**	NC_004572	0,9	46	-		-	Host-associated – human pathogen, causative agent of Whipple's disease; aerobic; mesophilic

### *fur *genes as part of a paralogous gene family

**Figure 1:**
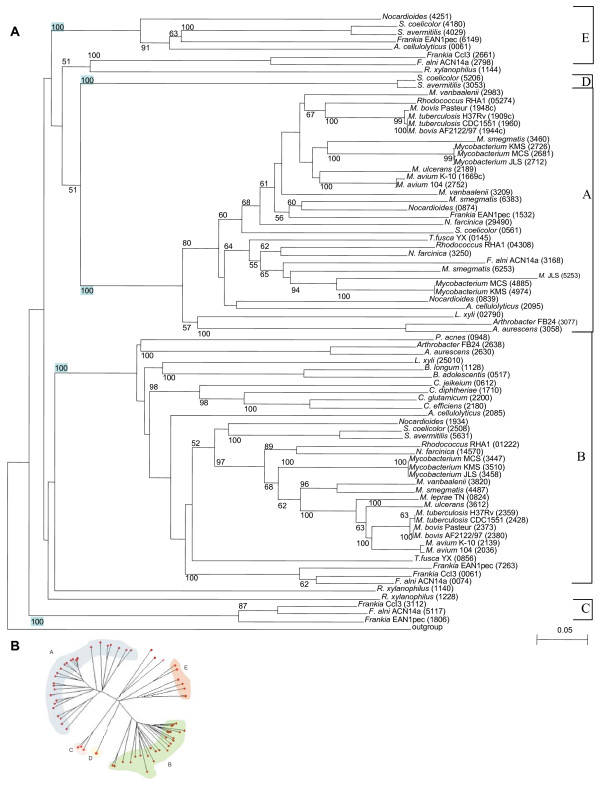
**Neighbour-Joining tree of the Fur homologues**. **A**. Phylogenetic tree of Fur amino acid sequences generated by NJ analysis. The numbers beside nodes are the percentages of bootstrap values calculated for 10000 replicates: only those above 50% are represented. Blue boxes highlight the high statistical significance of a few nodes crucial for the model described in the Fig. 2B. Each sequence is identified by the host species name and by the numerical part of the locus tag of each coding gene (in brackets). The five groups – A, B, C, D and E – mentioned in the text are indicated on the right side of the tree. **B**. Unrooted phylogenetic tree of Fur amino acid sequences generated by NJ analysis. The five groups – A, B, C, D and E – mentioned in the text are highlighted in different colours.

**Figure 2:**
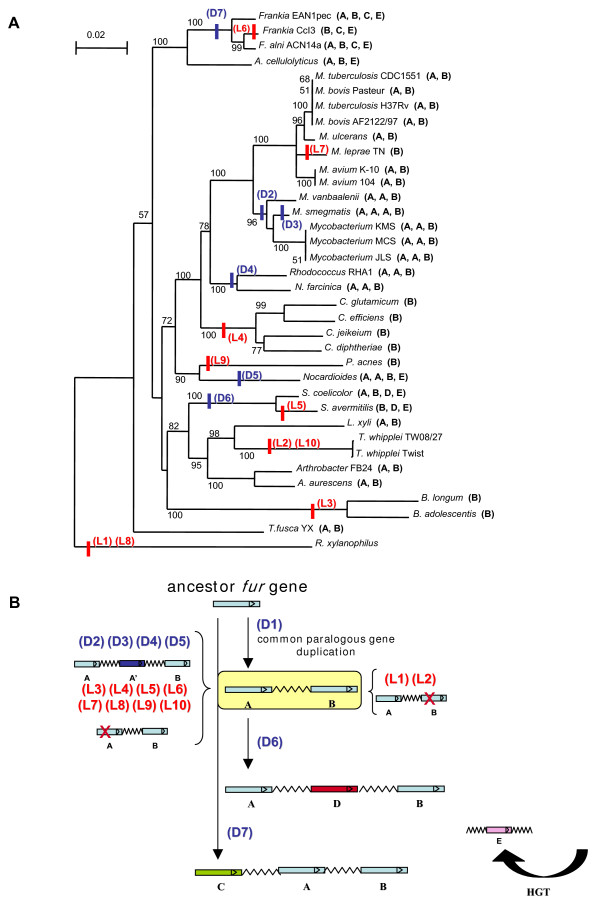
**Proposed model for *fur *evolution**. **A**. Phylogenetic tree of 16S nucleotide sequences generated by NJ analysis. The numbers beside nodes are the percentages of bootstrap values calculated for 10000 replicates: only those above 50% are represented. Evolutionary events are indicated by a short coloured line: blue for duplications and red for losses. In front of the species name, the number of homologues belonging to each one of the groups A, B, C, D and E is indicated. **B**. Schematic model of *fur *evolution. The losses and duplications referred on 2A are here located in terms of homologues gain and lost.

Since multiple *fur *homologues are present in many genomes, it seems logical that duplication and divergence from a common ancestor gene have occurred during their evolution. To test this hypothesis and to analyse the degree of similarity and identity among the different homologues, a multiple alignment was done using ClustalX [see Additional File 2] and a phylogenetic tree was computed using the Neighbor-Joining (NJ) method (Fig. 1). The distribution of the evolutionary distances and the tree topology strongly suggest that a duplication event took place before the divergence of the actinobacterial lineages, implying that two paralogues were already present in the last common ancestor. Duplication of *fur *after divergence would have yielded a tree more closely resembling the 16S tree (Fig. 2A). However, this pattern was not observed. Instead, homologues within each genome are almost always separated by a node close to the root, while orthologues from different organisms cluster together, with strong bootstrap support.

### Modelling *fur *evolution

We propose a duplication-based model to explain the evolutionary history that took place leading to the various *fur *genes in the Actinobacteria (Fig. 2B). According to this scenario, the ancestral organism possessed a regulator that had affinity for several metals and that could bind to various DNA sequences. This ancestral *fur *gene underwent a paralogous duplication event, giving rise to homologues designated as A and B in the Figs. 1 and 2. After the lineage leading to *Frankia *diverged, the initial *fur *gene underwent a second paralogous duplication event in this specific lineage, giving rise to the C homologue. Finally, in the progenitor of *Streptomyces*, the A homologue underwent a third paralogous duplication leading to the D homologue. The E homologues (Fig. 1), which are only present in seven copies, apparently had a different origin and will be discussed below.

**Figure 3:**
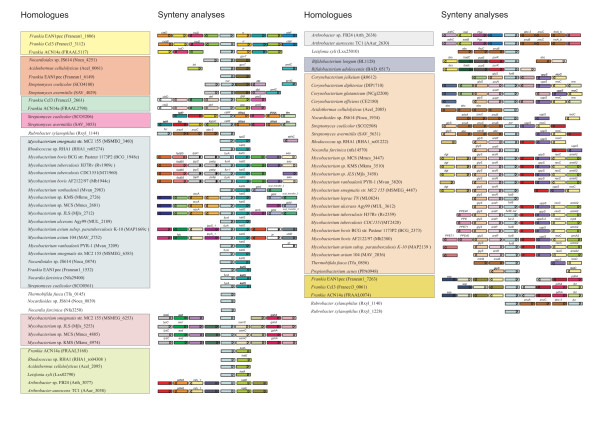
**Synteny analyses**. Schematic representation of the chromosomal regions surrounding *fur*. The blue bar in the middle represents the *fur *itself. The acronyms of the *fur *homologues that have been annotated with a name other than *fur *are indicated in the top of the bar. Four genes upstream and four genes downstream of the *fur *were analysed: the ones that present a significant degree of similarity (according to the parameters defined in the methods) have the same colour; the ones that do not have a significant degree of similarity with any other gene located in the explored regions are not represented. The position of each bar in the scheme is representative of its position in the genome relative to the considered *fur *homologue, and the arrow heads represent the transcriptional orientation. The annotation respects the one that is used in each genome. List of acronyms in order of appearance (left to right, top to bottom): ctaD, cytochrome c oxidase polypeptide I; trpD, anthranilate phosphoribosyltransferase 1; rr, rubrerythrin; ctaF, cytochrome c oxidase polypeptide III; cyoB, cytochrome-c oxidase; gcvT, glycine cleavage T protein (aminomethyl transferase); tst, thiosulfate sulfurtransferase; amfC, AmfC protein; dtd, D-tyrosyl-tRNA deacylase; cbiD, precorrin-6A synthase (deacetylating); def, peptide deformylase; gcp, sialoglycoprotein endopeptidase; ureG, rease accessory protein UreG; tetR, tetR-family transcriptional regulator; tRNA, tRNA-encoding DNA; itm, integral membrane protein; fbp, fructose-1,6-bisphosphatase; katA1, catalase; znuA, periplasmic solute binding protein; znuC, ABC transporter related; abc-3, ABC transporter related; katG, catalase-peroxidase; adhC, alcohol dehydrogenase; fadB5, oxidoreductase fadB5; lppC, lipoprotein lppC; aao, D-amino acid oxidase; pebp, phosphatidylethanolamine-binding protein; gdsl, lipolytic enzyme, G-D-S-L family; acyl_transfer_3, acyltransferase 3; accA, Propionyl-CoA carboxylase; anrk, aminoglycoside/hydroxyurea antibiotic resistance kinase; lppS_1, lipoprotein; fdo, FAD dependent oxidoreductase; gtr, glycosyl transferase; ubiE, methyltransferase; lysC, asparate kinase; asd, aspartate-semialdehyde dehydrogenase; gshA, glutamate-cysteine ligase; osmC, OsmC family protein; katE, catalase; pM48, peptidase M48, Ste24p; mfs1, major facilitator superfamily MFS_1; tst, 3-mercaptopyruvate sulfurtransferase; pyp, pyridoxal-5'-phosphate-dependent enzyme, beta subunit; tro_A, periplasmic solute binding protein; sseB, thiosulfate sulfurtransferase; fhu, ABC transporter, ATP-binding protein; dxs, 1-deoxy-D-xylulose-5-phosphate synthase; badC, NADPH:quinone oxidoreductase or alcohol dehydrogenase; purE, phosphoribosylaminoimidazole carboxylase catalytic subunit; purK, phosphoribosylaminoimidazole carboxylase ATPase subunit; glyS, glycyl-tRNA synthetase; uppS, undecaprenyl pyrophosphate synthase; recO, DNA repair protein RecO; arsR, ArsR-family transcriptional regulator; era, GTP-binding protein Era; adcA, periplasmic solute binding protein; mtnB, manganese/zinc transport system ABC transporter ATP-binding protein; dgt, deoxyguanosinetriphosphate triphosphohydrolase; amiA2, amidase; PPE40, PPE family protein; PPE, PPE family protein; upps-2, undecaprenyl diphosphate synthase; PPE71, PPE family protein; asp, aspartate aminotransferase; pdhA, pyruvate dehydrogenase E1, alpha subunit; tkt, transketolase.

These duplication events were followed by specific losses and further duplications that likely modulated the response capacity of each organism to the particular set of evolutionary pressures that characterise its ecological niche. Based on the model described above, one would expect to find one homologue of each kind in each taxon. While the B homologue was generally conserved – there are only two losses recognizable in *R. xylanophylus *and in *Tropheryma *spp. – the A homologue was the object of several duplications and losses. This pattern suggests that the selective pressures acting on this homologue were more variable than the ones affecting the B homologue. Seven organisms have two A homologues and one organism has three A homologues (Table 1). Based on the principle of parsimony, and looking at the 16S tree (Fig. 2A), one can postulate four gains to explain these nine additional A homologues (Fig. 2): one in the common ancestor of *M. smegmatis*, *Mycobacterium *JLS, *Mycobacterium *KMS, *Mycobacterium *MCS and *M. vanbaaleni*; one in the common ancestor of *N. farcinica *and *Rhodococcus *RHA1; one *M. smegmatis *and another in *Nocardioides*. Given the synteny surrounding some orthologues of each gain (Fig. 3), duplication events are the most parsimonious explanation. This issue will be further discussed below. On the other hand, thirteen organisms do not retain the A homologue, which can be explained by eight independent gene losses (Fig. 2): one in the common ancestor of *B. adolescentis *and *B. longum*; one in the common ancestor of *C. diphtheriae*, *C. efficiens*, *C. glutamicum *and *C. jeikeium*; one in the common ancestor of *Tropheryma whipplei *TW08/27 and *Tropheryma whipplei *Twist; and one in each of *S. avermitilis*, *Frankia *CcI3, *M. leprae*, *P. acnes *and *R. xylanophylus*.

Taking the two major groups originating from the first duplication, one can speculate why A homologues are subjected to such strong and diverse selective forces when compared to the more conserved B homologues. As mentioned previously in the introduction, A homologues have been extensively studied in several Actinobacteria, and at least in *Mycobacterium *and *Streptomyces *spp. they control the transcription of a downstream catalase-peroxidase, having therefore a major role in the oxidative stress response. Given that this kind of stress is inherent to all oxygen-consuming organisms and able to affect many molecules inside a living cell, it is not surprising to find that anti-oxidant mechanisms and their regulators are quite sensitive to evolutionary pressures related to each specific ecological niche. Since orthologues most likely retain their function across different species, it is reasonable to argue that the A homologues are involved in oxidative stress response regulation. On the other hand, B homologues may either be involved in regulating a cellular function more conserved across organisms, or its involvement in cellular metabolism is not as broad as in a situation of oxidative stress, and therefore these homologues are more stable across organisms throughout geological time.

The E group of sequences likely originated from three HGT (horizontal gene transfer) events: one to the *Streptomyces *ancestor (originating SCO4180 and SAV_4029), and then from that to the *Frankia *– *Acidothermus *common ancestor (originating Acel_0061, Franean1_6149, Francci3_2661 and FRAAL2798) and to *Nocardioides *(originating Noca_4251) in two separate horizontal transfers. These transfers would include the *fur *and the surrounding genes which, in parallel with the *fur*-*katG *case, could be putatively regulated by Fur constituting an operon. In the common ancestor of *Frankia *CcI3 and ACN14a, a reshuffling of the genome could be responsible for disrupting of the genomic context, which is maintained among Noca_4251, Acel_0061, Franean1_6149, SCO4180 and SAV4029 (Fig. 3). This hypothesis can explain both the presence and the synteny encountered among these genes, and is supported by their %GC values – FRAAL2798, Francci3_2661, SAV4029 and SCO4180 have a %GC value that deviates from the genome (Table 1). Lastly, with the exception of *A. cellulolyticus*, all these organisms inhabit soil ecological niches which facilitates the occurrence of HGT.

Finally, the *R. xylanophilus *homologues are the only ones that are not considered in our model – they seem to have been acquired independently from other Actinobacteria by three individual HGTs. In fact, their %GC values are different both among the sequences and comparing with the genome. A single HGT followed by duplication is also an hypothesis to be considered, especially since it is known that laterally transferred genes have higher rates of duplication [33].

In order to validate the NJ results and to evaluate the strength of the proposed model, a Maximum-Likelihood (ML) tree was computed [see Additional File 3]. The outcome of it corroborates the NJ phylogeny. Despite the fact that the ML tree is not completely resolved, having 14.8% unresolved quartets, it is clear that the groups identified by NJ are maintained, as well as the relations between them. In fact, the C group of homologues emerges from a node close to the root, while the D group of homologues is related to the A group, which suggests that C had its origin by duplication of the ancestor gene while D had its origin on a duplication of the A.

### From Actinobacteria to the big picture

One interesting question regarding evolution of Fur in Actinobacteria is whether the first duplication occurred in the ancestor of the Actinobacteria, or if it was an earlier event that would have appeared in other lineages. To address this question, we randomly selected three different species from each Eubacteria group (except in cases like Acidobacteria where fewer than three genomes are available) and investigated the presence of Fur homologues combining a relaxed BlastP screening against each genome individually with annotation information. The resulting sequences were aligned with the Fur homologues from the Actinobacteria [see Additional File 4] and a NJ tree was computed [see Additional File 5]. The first bifurcation of this tree divides it into two major groups: one of them contains the actinobacterial A homologues and the other contains the actinobacterial B homologues, indicating that the initial duplication occurred in the eubacterial common ancestor. This tree also supports a common origin for the E group of homologues, which are clustered together and separated from the other actinobacterial groups. Multiple origins for this group, either by HGT or duplication, would result in the distribution of these homologues all through the tree. Their clustering shows that either they resulted from a common duplication that was lost in all the other Actinobacteria, or, more parsimoniously, that they resulted from the described HGT. The *R. xylanophylus *homologues appeared later, which results in a scattered distribution in the tree, supporting an origin in independent HGT events.

Interestingly, previously published work concerning the evolutionary history of Fur and other iron and manganese-responsive transcriptional regulators in alphaproteobacteria [34] indicates that most of bacteria in this lineage have only one Fur, involved in iron homeostasis regulation, which evolved towards a manganese-homeostasis regulator (Mur) in Rhizobiales and Rhodobacteraceae. However, and in the same study, another regulator present in some of the alphaproteobacteria and named Irr was characterised and considered to be part of the Fur superfamily. Putative Irr-binding sites have been found upstream of genes encoding iron-homeostasis and iron-containing proteins, in particular catalase-peroxidases, suggesting that this regulator might functionally correspond to the actinobacterial oxidative stress-related Fur. Although Rodionov et. al (2006) have studied the Fur/Mur phylogeny separately from the Irr one, the fact that they are placed in the same superfamily suggests a common origin, thus supporting the hypothesis that the first Fur duplication occurred in the eubacterial common ancestor, from which a group related with oxidative stress response has emerged.

### Duplicate to evolve

The proposed evolutionary scenario for *fur *is consistent with the current view of gene duplication as a major means of microbial genome evolution [35]. It has been suggested that broadly functional genes are more easily duplicated than functionally established ones, and that the modifications that follow the duplications should provide the appearance of new functional specificities. In fact, although paralogues and orthologues have the same general function, paralogues usually differ in specific biochemical details such as the primary target or a required co-factor [36]. Thus, it is reasonable to conceive that an ancestral *fur *gene, encoding a Fur protein with a broad range of DNA-binding motifs and ionic co-factors, through duplication and divergence, gave rise to the modern *fur *genes, now optimised and specialised.

Consistent with the described model are the results of independent biochemical characterization of three of the *S. coelicolor *homologues: one of them (SCO0561 – group A) is able to bind *in vitro *several divalent metals (Ni^2+^, Mn^2+^, Zn^2+ ^and Fe^2+^) and regulates the downstream catalase-peroxidase in a redox-dependent manner [22]; a second (SCO4180 – group E) binds specifically Ni^2+ ^and regulates the transcription of a FeSOD and a cluster of genes related to nickel-uptake [9]; and the third (SCO5206 – group D) binds metals yet to be identified, responds to the redox changes of the cell and regulates a monofunctional catalase [18]. In the same way, the biochemical characterisation of the Fur homologues in *M. tuberculosis *have shown that the A homologue (Rv1909c) regulates the downstream catalase-peroxidase in the presence of metals and in a redox-dependent manner [21,24], while the B homologue (Rv2359) binds Zn^2+ ^and is likely involved in the regulation of genes responsible for zinc-uptake [10]. Thus, the main function – transcription regulation – is maintained among paralogues and orthologues. However, functional specificities such as the metal coordinated and the genes regulated are only maintained within orthologues (SCO0561 and Rv1909c), and diverge in the paralogues of the same organism (SCO0561/SCO4180/SCO5206 and Rv1909c/Rv2359). As might be expected, paralogues resulting from recent duplications are more similar than more ancient ones. In fact, SCO5206, which according to the present model is predicted to be the result of a duplication of the A homologue, is functionally closer to its correspondent A paralogue (SCO0561) than to the others. Although they regulate different genes, both are functionally related to oxidative stress response and dependent on the redox status of the cell. Paralogues from older duplication events have had more time to evolve and have accumulated more differences than those generated from recent duplication events.

### Genomic context

In support of the proposed evolutionary model, it can be observed that several *fur *orthologues are located in equivalent regions of their genomes (Fig. 3). Syntenic genes reveal the core chromosomal segments present in a common ancestor, encoding a high proportion of essential gene functions and presenting a significantly lower HGT rate [37]. These syntenic regions are evidence for a group with a common origin, and were considered significant whenever at least two genes remained contiguous across different chromosomes [37]. The high degree of synteny observed for the *fur *orthologues points toward an early origin. The identification of the regulators that were lost or gained in each specific case may provide clues concerning the metabolic properties and pathways that are common to the lineage and those which specificity is more species-related.

### Correlation between genome size, ecological niche and Fur homologues

Assuming that genome size is somehow related to the selective pressures acting upon an organism, and that Fur is a global transcriptional regulator able to sense different metals and to regulate the expression of different genes, we argue that the larger the genome size and the underlying selective pressures, the higher is the need for Fur regulators and the lower the pressure toward gene loss. Indeed, the number of Fur homologues tends to increase with increasing genome size. As seen in Table 1, organisms with genomes smaller than 2 Mbps have no Fur homologues, organisms with genomes between 2 and 5 Mbps have 1 or 2 Fur homologues, while organisms with genomes between 5 and 7 Mbps have 3 Fur homologues and organisms with genomes between 7 and 9 Mbps have 4 Fur homologues, with seven exceptions:*A. cellulolyticus*, *M. avium *104, *M. ulcerans*, *Nocardioides *JS614, *Rhodococcus *RHA1, *R. xylanophilus *and *S. avermitilis*. This relation finds support in statistical analysis: the Pearson Product Moment Correlation was calculated using genome size and number of Furs as variables, and the result was 0.823, statistically different from 0 with α = 0.05.

*T. whipplei *spp. is an interesting case. Adaptation to a strictly host-adapted lifestyle has led to gene loss and several metabolic pathways, namely those related to amino acid biosynthesis and energy production, have been lost [38]. In this scenario, the need for regulators is reduced, leading to the loss of Fur proteins. On the other hand, the ecological niche occupied by each organism may explain the seven exceptions noted above. *A. cellulolyticus*, *Nocardioides *JS614, and *R. xylanophilus *have one more Fur than predicted by their chromosome size. While the first organism is a thermophile and the second one inhabits the soil, the third organism exhibits high tolerance to radiation. These considerable stressful situations may have imposed selective pressures that maintained an extra *fur *homologue in the chromosome, despite its size. On the other hand, *M. avium *104 and *M. ulcerans *have one less homologue than predicted by their chromosome size. This may be explained by the fact that these organisms are host associated: it is known that host-associated bacteria tend to undergo a genome reducing process. Finally, *Rhodococcus *RHA1 and *S. avermitilis *also have one less homologue than predicted by their chromosome size. There is no obvious explanation for these cases.

Frankiae, a group of plant microsymbionts able to fix atmospheric nitrogen, illustrates what is stated above. Recently, three *Frankia *strains have been sequenced, ACN14a, EAN1pec and CcI3, and the presence of a high number of transcriptional regulators has been noted [39]. Interestingly, these genomes present highly divergent sizes: ACN14a has a genome of 7.5 Mbps, encoding 6711 proteins; EAN1pec has a genome of 9.0 Mbps, encoding 7976 proteins; and finally, CcI3 has a genome of 5.4 Mbps, encoding 4499 proteins. One possible explanation proposed to account for the differences in genome size is related to the variation in their lifestyles. While ACN14a and EAN1pec survive well in the soil, CcI3 appears to be undergoing a genome reducing process, becoming more and more dependent on the plant symbiont and less able to survive by itself [39]. The increased gene contents of ACN14a and EAN1pec provide a variety of "extra" genes that allow these strains to survive in a variety of soils and in symbiosis. CcI3 is apparently losing its ability to survive in the free-living state so selective pressures are lower and fewer regulators are needed. This is clearly in agreement with the number of Fur homologues that were identified for each strain:*Frankia *ACN14a and EAN1pec have 4 homologues each, while CcI3 has only 3.

### Alternative hypotheses

As mentioned above, there are nine gains of the A homologue that cannot be explained by the proposed model. Although four independent duplications appear as the most parsimonious explanation for their origin, the hypothesis of HGT should also be considered, especially since six out of the nine homologues have a %G+C content different from the genome (MSMEG_6383, Mjls_5253, Mkms_4974, Mmcs_3447, nfa3250 and Noca_0874). The presence of synteny within at least one of the groups of homologues (MSMEG_6253, Mjls_5253, Mmcs_4885, Mkms_4974) favours the hypothesis of duplication but it does not exclude HGT, since the transfer of entire chromosomal fragments (instead of single genes) is possible.

On the other hand, one of the factors that limits HGT is that the transferred genes must outcompete indigenous ones, which are already part of a complex and adapted network, in order to be fixed in the genomes [40,41]. One expects essential regulatory genes to be stable during evolution. In fact, it is not simple for a regulator like *fur *to be horizontally transferred, enter a complex network, and establish itself as a major regulator. Furthermore, recent work has suggested that HGTs seldom affects orthologues [42]. Therefore, due to the number of the *fur *homologues in the genomes, their synteny and their nature as global regulators, explaining the presence of these genes by HGT should be used with care, and consequently %G+C value alone should not be the exclusive argument to sustain a HGT situation.

Regarding the E sequences, the situation is inverted. Besides %G+C values, other factors seem to favour HGT. One could hypothesise three independent duplication events as the origin of these genes: in the *Streptomyces *ancestor, in the *Frankia *– *Acidothermus *common ancestor and in *Nocardioides*. However, the presence of synteny across these different groups – and not only within them, as happens in the duplicated A homologues – indicates a common origin for all of the genes that constitute them, excluding the three independent duplications as well as three independent HGTs. Another explanatory hypothesis would include a single genomic duplication in the last common ancestor of the organisms involved. However, this last common ancestor is actually the last common ancestor to most of all the other Actinobacteria considered, and so the possibility of duplication would imply a high rate of gene loss. In fact, and in terms of number of evolutionary steps, the 3 in-tandem described HGT is the most parsimonious explanation for this group of genes.

## Conclusion

The abundance of *fur *genes in Actinobacteria and their phylogenetic relationship points towards early duplications in the evolution of these regulators, along with additional HGT and later intra-species duplications. A strong synteny between *fur *orthologues regions is consistent with the proposed model and supported by functional studies. These observations provide clues for future studies concerning the importance of Fur in regulating other systems besides oxidative stress in organisms inhabiting diverse ecological niches and under dissimilar selective pressures. Furthermore, they help to differentiate between the basic essential processes and the species-specific ones. Exploring the phylogeny of regulators at the same time as their functionality and the organisms' ecology is a promising strategy to explore how different bacteria adapt to their various habitats and lifestyles by a fine-tune control of DNA transcription.

## Methods

### Blast searches and sequences retrieval

In order to identify all the *fur *homologues in the completely sequenced actinobacterial genomes present in NCBI database (March/2007), a two-step approach was used. Initially, BlastP analyses were performed against each genome individually, using *Frankia alni *ACN14a FRAAL3168 as the query sequence: only hits with an e-value below or equal to e-05 were retained for further analyses. Afterwards, the retrieved sequences were aligned with *Streptomyces reticuli *FurS (CAA74697), in order to check for the presence of five key residues shown by D. Ortiz de Orué Lucana et al. (2003) to be essential for Fur functionality: cysteines 96 and 99, histidines 92 and 93 and tyrosine 59.

### Multiple alignments and phylogenetic trees

Multiple alignments were performed using ClustalX 1.81 [43] with all the default parameters. The data set included the Fur homologues' amino-acid sequences, with or without *Archaeoglobus fulgidus *DSM 4304 Fur (NP_071057) as the outgroup, and 16S ribosomal RNA nucleotide sequences [see Additional File 6] retrieved from each genome page on NCBI. The resulting alignments were used to generate phylogenetic trees by the Neighbor-Joining (NJ) method [44] using the same software, and by the Maximum Likelihood (ML) method [45] using Tree-Puzzle 5.2 [46]. Bootstrap values were calculated for the NJ trees using 10000 replicates to evaluate the robustness of the nodes [47].

For the ML analysis, the evolution model used was the WAG model [48], selected by Tree-Puzzle as being the one that best described our data. The parameter estimation was exact and used quartet sampling (for substitution process) and NJ data (for rate variation). The chosen tree search procedure was Quartet Puzzling and 50000 puzzling steps were computed in order to obtain the consensus tree.

Trees were visualised by NJPLOT [49] and PhyloDraw 0.8 [50].

### Synteny analyses

For each *fur *homologue, the adjacent regions were visually inspected in order to detect the presence or absence of synteny. The inferred amino acid sequences for the 4 genes found upstream and downstream of each *fur *were used as query in a BlastP search against all the sequenced actinobacterial genomes, and those with e-values below e-05 were analysed to determine if any hit corresponded to a gene-encoding protein occupying a similar position relative to a *fur *orthologue in another organism.

### Statistical analysis

The Pearson Product Moment Correlation was computed using XLSTAT 2008.2.02.

## Authors' contributions

CLS, JV, FT and PN conceived the study. CLS collected and analysed the data and wrote the manuscript. DRB, LST, AMB and PM–F assisted in the drafting and provided substantial editorial advice and a critical revision of the manuscript. FT and PN helped in coordinating the study. All authors have read and approved the manuscript.

## Supplementary Material

Additional File 1 Multiple alignment of the studied Fur homologues + *Streptomyces reticuli *FurS. Click here for file

Additional File 2 Multiple alignment of the studied Fur homologues. Click here for file

Additional File 3 Maximum-Likelihod tree of the Fur homologues. Click here for file

Additional File 4 Multiple alignment of the Fur homologues (global approach). Click here for file

Additional File 5 Neighbour-Joining tree of the Fur homologues (global approach). Click here for file

Additional File 6 Multiple alignment of the 16S sequences. Click here for file
