# Supplementary material for: On the nature of fur evolution: A phylogenetic approach in Actinobacteria
Source: BMC Evol Biol. 2008 Jun 25;8:185. doi: 10.1186/1471-2148-8-185 (PMC2464607; doi:10.1186/1471-2148-8-185)

```
           SAV4029 -------------------------------------------MVS--TDWKNDL----------RQRGYRLTPQRQLVL   25
           SCO4180 -------------------------------------------MVS--TDWKSDL----------RQRGYRLTPQRQLVL   25
Franean1DRAFT_3571 -------------------------------------------MAAKVDDWHTRL----------RARGYRLTPQRQLVL   27
         Acel_0061 -----------------------------------MPKGSSDRPSTRSAQWQGRL----------RSKGYRLTPQRRLVL   35
         Noca_4251 --------------------------------------------MT--EELAARL----------REQGYRLTPQRQLVL   24
         Rxyl_1228 --------------------------------------------MMEFEQIL-------------RSRGYRLTNQRRVIV   23
         Rxyl_1140 ----------------------MTPDPPPGSLSRVVEKFFQLGGDLELEAVLGRM----------RGRGYKVTPQRLAVL   48
         AAur_2630 ----------------------------------MGFASIPREVSLSMPQGTSSATTGNEAPATSGVKEQRVTKQRLAVS   46
         Arth_2638 -----------------------------------------------MAHGAKAEGTKLSAPASAGGREQRVTKQRLAVS   33
          MAV_2036 -----------------------------------------------MTPSGDG-----------AGVSVRSTRQRAAIS   22
           MAP2139 -----------------------------------------------MTPSGDG-----------AGVSVRSTRQRAAIS   22
            Mb2380 -----------------------------------------------MSAAG-----------------VRSTRQRAAIS   16
          BCG_2373 -----------------------------------------------MSAAG-----------------VRSTRQRAAIS   16
            MT2428 -----------------------------------------------MSAAG-----------------VRSTRQRAAIS   16
            Rv2359 -----------------------------------------------MSAAG-----------------VRSTRQRAAIS   16
          MUL_3612 -----------------------------------------------MTGTS-----------------VRSTRQRAAIS   16
            ML0824 ----------------------------------------------MMTGAS-----------------VRSTRQRAAIS   17
        MSMEG_4487 -----------------------------------------------MTGA------------------VRSTRQRAAIA   15
         Mvan_3820 -----------------------------------------------MTGA------------------VRSTRQRAAIS   15
         Mjls_3458 -----------------------------------------------MSPAP-----------------VRATRQRAAIA   16
         Mkms_3510 -----------------------------------------------MSPAP-----------------VRATRQRAAIA   16
         Mmcs_3447 -----------------------------------------------MSPAP-----------------VRATRQRAAIA   16
          nfa14570 -----------------------------------------------MQDKTSAP-------QKP--VGIRSTRQRSAIA   24
      RHA1_ro01222 -----------------------------------------------MTQNTGQ--------RKPVVVGVRATKQRSAIS   25
           SAV5631 -----------------------------------------------MTTAGSP-------------VRGRSTRQRAAVA   20
           SCO2508 -----------------------------------------------MTTAGPP-------------VKGRATRQRAAVS   20
         Noca_1934 -----------------------------------------------MTSTEHAE-------TP----RLRPTRQRRAVT   22
         FRAAL0074 ---------------------------------------------------------------------MRATRQGDAVC   11
     Francci3_0061 -----------------------------------------------MERRPAPSRGT----------AVRATRQGDAVS   23
Franean1DRAFT_4289 --------MDVVASVEGERVSSPRGMSAASDRAAGGAGAGSGDRPNGAVSRPGRGRGT----------SVRATRQGDAVS   62
          Tfu_0856 ---------------------------------------------------------------------MSSRR--EAVR    9
         Acel_2085 -----------------------------------------------MSGSSEGR-------------QSRSTRQKRALA   20
          BAD_0517 -----------------------------------------------MSEHI-----------------VRQTRQKDAIR   16
            BL1128 -------------------------------------------MTLPMVEHI-----------------ERQTKQKDAIR   20
          Lxx25010 --------------------------------------------------MV-----------------KRNTWQREAVR   13
            CE2180 ------------------MLKKPSRPLRSRIRGNNHCFPDSQEGGMGLNRHGEKS---------TPKLGVRSTRQRKAVV   53
          NCgl2200 ---------------------------------------------MGINRISQGS---------APKLGVRSTRQRKAVI   26
           DIP1710 -----------------------------------------------MNRTIDRS---------VPKLGVRSTRQRTAVV   24
            jk0612 -----------------------------------------------MSNPATNK---------LPKIGQRNTRQRRAVA   24
           PPA0948 -----------------------------------------------MENRR------------------RTTKQRLAIR   15
         FRAAL5117 ----------------------------------------------------------------------------MAVL    4
     Francci3_3112 -----------------------------------------------MNDQT----------------DLRLTPQRMAVL   17
Franean1DRAFT_4643 -----------------------------------------------MPERT----------------ELRLTPQRTRVL   17
         FRAAL2798 --------------------MEDRRPVGAAAATAAAGHDALPGRRDDLAAAGIAELR---------ALGERVTPARRAVL   51
     Francci3_2661 --------------------MKDTWIVKDSQTDRD-------GRRGDPSAAGIAALR---------ARGERVTPARRAVL   44
         Rxyl_1144 ------------------------------------------------------------------MSFRRRTRQREAIL   14
           SAV3053 -----------------------------------------------MSDLLERLR----------GRGWRMTAQRRVVA   23
           SCO5206 -----------------------------------------------MSDLLERLR----------GRGWRMTAQRRVVA   23
         Acel_2095 --------------------------------------------MRSPVDRLR-------------AAGMRVTPARLAVL   23
         Noca_0839 --------------------------------------------MAPAEEDLR-------------AAGLRVTRPRVAVL   23
         Mkms_4974 --------------------------------------------------------------------------------
         Mmcs_4885 --------------------------------------------------------------------------------
         Mjls_5253 ---------------------------------------MTPARETTPEARLR-------------ASGLRVTAPRLAVL   28
        MSMEG_6253 ---------------------------------------MTTVHDHDPKALLR-------------ASGLRVTAPRVAVL   28
         FRAAL3168 -------------------------------------------MATDRATTLR-------------GAGLRVTGPRLAVL   24
           nfa3250 ----------------------------------------MHTETAEPRDRLR-------------AAGLRVTAPRLAVL   27
      RHA1_ro04308 ----------------------------------------MRHDT-DPKAQLR-------------EVGLRVTAPRVAVL   26
          Tfu_0145 ---------------------------------------------MDDAASLR-------------AAGLRVTAARLAIL   22
         Mjls_2712 --------------------------------------MAVVSSSSEYVDQLR-------------GADLRVTRPRVAVL   29
         Mmcs_2681 MITPLGLKSRICLARRPIGVSAQNARDELLTYSKKAGDMAVVSSSSEYVDQLR-------------GADLRVTRPRVAVL   67
         Mkms_2726 --------------------------------------MAVVSSSSEYVDQLR-------------GADLRVTRPRVAVL   29
        MSMEG_3460 -----------------------------------------MPSRAEFEAQLR-------------MTDLRVTRPRIAVM   26
          MAV_2752 ------------------------------------------------------------------MADLRVTRPRVAVL   14
          MAP1669c -----------------------------------------MSSTADYADRLR-------------MADLRVTRPRVAVL   26
          MUL_2189 ------------------------------------------------------------------MADLRVTRPRLAVL   14
           Mb1944c --------------------------------------MSSVSSIPDYAEQLR-------------TADLRVTRPRVAVL   29
            MT1960 --------------------------------------MSSVSSIPDYAEQLR-------------TADLRVTRPRVAVL   29
           Rv1909c --------------------------------------MSSVSSIPDYAEQLR-------------TADLRVTRPRVAVL   29
         BCG_1948c --------------------------------------MSSVSSIPDYAEQLR-------------TADLRVTRPRVAVL   29
      RHA1_ro05274 --------------------------------------------MPDYAALLR-------------GAELRVTRPRVAVL   23
         Mvan_2983 -------------------------------------------MTSDYAELLR-------------GADLRVTRPRIAVL   24
Franean1DRAFT_1681 --------------------MVAWLLSGFLDWFQKMGDHVVVATAEDFRRMLR-------------GAALRVTRPRVAVL   47
         Noca_0874 -------------------------------------MATTTDRTRDFPELLR-------------GAALRVTRPRLAVL   30
        MSMEG_6383 -----------------------------------------MTHTTDFEQLLR-------------SAGMRVTRPRIAVL   26
         Mvan_3209 --------------------------------------MEPMTEEPDYSAVLR-------------SAALRVTRPRMAVL   29
          nfa29490 -----------------------------------------MSKVPDFEQLLR-------------GASLRVTAQRLAVL   26
           SCO0561 ------------------------------------MTASRNSRKPATADELR-------------RAGLRVTAARVALL   31
              FurS ------------------------------------MTASP---TPTTAEELR-------------GAGLRVTAARVALL   28
         AAur_3058 -------------------------------------MTDHTTEQAEWAAALH-------------AHGRRVTKQRLAVL   30
         Arth_3077 -------------------------------------MTVHEAGQDAWAAALR-------------AHGRRVTKQRLAVL   30
          Lxx02790 ------------------------------------------MDTAQLEDTLR-------------SAGLRVTRGRLAVL   25
         Rxyl_1224 --------------------------------------MAGFCMTERLEEDIREKFR---------RSGYTLTSQRRAVL   33
         Mjls_1895 -------------------------------------------MAAAVEDGVG-----------YLVKRVQQSLRRRCDA   26
             ruler 1.......10........20........30........40........50........60........70........80
```

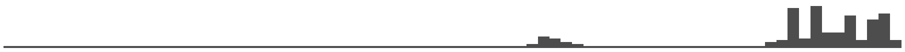

```
                                                            :                                    .
            SAV4029 EAVDKLE-HATPDDILVEVRKTASGVNISTVYRTLELLEELGLVSHAHLGHGAPTYHLADRH---HHIHLVCRDCTNVIE   101
            SCO4180 EAVDTLE-HATPDDILGEVRKTASGINISTVYRTLELLEELGLVSHAHLGHGAPTYHLADRH---HHIHLVCRDCTNVIE   101
Franean1DRAFT_3571 EAVARLG-HATPEAIVHEVRQTASGVNISTVYRTLDLLEELDLVSHAHLSHGAPTYHVTGVD---PHVHLVCGACGAILE   103
          Acel_0061 EAVAALR-HATPEEIINYVREVASGVNMSTVYRTLDLLEELSLVTHTHLGHGAPTYHVAEDA---GHVHLVCSSCGAVTE   111
          Noca_4251 QAVEKLG-HATPDEVLAEVHAQSSAVNVSTIYRTLEVLEELGLVRHAHLSDRAPTYHSVTDR---EHFHLVCRNCHKVVS   100
          Rxyl_1228 RELEGER-HLSAEEIYERVKEEHPELGLSTVYRTLDLLHELGIVRKEDFGEGYSRYELATER---MHHHARCRGCGKVIE    99
          Rxyl_1140 EALAAER-HQSLEELRGRC----PEVGLATLYRTLDLLTGIGAVRRLELGDG-PRYELAGD----HHHHLICEACGEISE   118
          AAur_2630 AALDELDDFVSTQELYRLLQNKGISVSLATAYRILQSLADDGLIDVLRNGDGEAVYRRCAV--TGHHHHLLCRNCGKAEE   124
          Arth_2638 AALDELDDFVSTQELYRMLQNQGTSVSLATAYRILQSLADDGLVDVLRNGEGEAVYRRCAV--TGHHHHLLCRNCGKAVE   111
           MAV_2036 TLLETVDDFRSAQELHDELRRRGENIGLTTVYRTLQSMAAAGLIDTLRTDTGESVYRRCS---EHHHHHLVCRSCGSTIE    99
            MAP2139 TLLETLDDFRSAQELHDELRRRGENIGLTTVYRTLQSMAAAGLIDTLRTDTGESVYRRCS---EHHHHHLVCRSCGSTIE    99
             Mb2380 TLLETLDDFRSAQELHDELRRRGENIGLTTVYRTLQSMASSGLVDTLRTDTGESVYRRCS---EHHHHHLVCRSCGSTIE    93
           BCG_2373 TLLETLDDFRSAQELHDELRRRGENIGLTTVYRTLQSMASSGLVDTLRTDTGESVYRRCS---EHHHHHLVCRSCGSTIE    93
             MT2428 TLLETLDDFRSAQELHDELRRRGENIGLTTVYRTLQSMASSGLVDTLHTDTGESVYRRCS---EHHHHHLVCRSCGSTIE    93
             Rv2359 TLLETLDDFRSAQELHDELRRRGENIGLTTVYRTLQSMASSGLVDTLHTDTGESVYRRCS---EHHHHHLVCRSCGSTIE    93
           MUL_3612 TLLETVDDFRSAQELHDELRRRGENIGLTTVYRTLQSMASAGTVDTLRTDTGESVYRRCS---QHHHHHLVCRSCGSTIE    93
             ML0824 TLLETVDDFRSAQELHNELRRRGNNISLTTVYRTLQSMAEAGMVDTLRTNTGESVYRRCS---RQHHHHLVCRGCGSTIE    94
         MSMEG_4487 DLLNETEGFRSAQELHDELRRRGEGIGLTTVYRTLQAMATAGVVDTLRTDTGESVYRRCS---EHHHHHLVCRSCGSTVE    92
          Mvan_3820 ALLENIEDFRSAQELHDELRRRGEGIGLTTVYRTLQQMAAAGVVDTLRTDTGESVYRRCS---EHHHHHLVCRACGCTVE    92
          Mjls_3458 DLLDGLDEFRSAQELHDALKRRGEGIGLTTVYRTLQSMAESGLVDVLRTDTGESVYRRCS---ADHHHHLVCRACGAAVE    93
          Mkms_3510 DLLDGLDEFRSAQELHDALKRRGEGIGLTTVYRTLQSMAESGLVDVLRTDTGESVYRRCS---ADHHHHLVCRACGAAVE    93
          Mmcs_3447 DLLDGLDEFRSAQELHDALKRRGEGIGLTTVYRTLQSMAESGLVDVLRTDTGESVYRRCS---ADHHHHLVCRACGAAVE    93
           nfa14570 ALLGDIDEFRSAQELHDELRKRGEGIGLTTVYRTLQSLADAGMVDVLRTDSGESVYRQCS---SGHHHHLVCRHCGRTVE   101
       RHA1_ro01222 ALLDDIEEFRSAQELHDELRKRGEGIGLTTVYRTLQTLADAGTVDVLRTDTGESVYRRCS---SGHHHHLVCRTCGYTVE   102
            SAV5631 AALDEVDEFRSAQELHDMLKHKGDSVGLTTVYRTLQNLADAGEVDVLRTSDGESVYRRCSS--GEHHHHLVCRVCGKAVE    98
            SCO2508 AALQEVEEFRSAQELHDMLKHKGDAVGLTTVYRTLQSLADAGEVDVLRTAEGESVYRRCST--GDHHHHLVCRACGKAVE    98
          Noca_1934 EALDSFSDFRSAQEIHELLGRRGETVGLATVYRTLQRLAEAGDVDVLRTEDGEAIYRRCS---EAHHHHLVCRSCGATVE    99
          FRAAL0074 AALTETDAFTSAQELHALLRSRGAGVGLTTVYRHLQVLVDRGEVDMIRRDDGETVYRRCAT--EAHHHHLVCRLCGRTVE    89
     Francci3_0061 AALAETDAFTSAQDLHARLRAQGAAVGLTTVYRHLQVLADRGEVDMIRRDDGETVYRRCAS--DDHHHHLVCRSCGRTVE   101
Franean1DRAFT_4289 SALAATNAFTSAQDLHARLRQEGQSVGLTTVYRHLQVLADRGEVDVLRRDDGETVYRRCTT--DGHHHHLVCRHCGHTVE   140
           Tfu_0856 QALHKSNGFRSAQDLYASLRADGAKIGLTTVYRALQALTDAGEVDVLMTDEGEAVYRACST--PTHHHHLVCRDCGKAVE    87
          Acel_2085 QVMAESRQFRSAQELHEALIDRGESVGLTTVYNQLRAMAEAGLVDTLRAPDGETLYRQCET--NAHHHHLRCRYCGRTVE    98
           BAD_0517 DALNGCDEFISAQELHRKLEDRGEHIGLATVYRQLNALAEAGRADTVR-LDGQQLFRLCGD--DGHHHHLVCTNCGKTVE    93
             BL1128 AALADCEEFISAQDLHRRLEDEGSKIGLATVYRQLNALADAGAADTIR-LDGQQLFRLCGD--DGHHHHLVCRRCGKTVE    97
           Lxx25010 EALSSTEGFISAQGLHLRLHEAGSPIGLATVYRALADLAAEGDADSLQSPEGESLYRACTS---GHHHHLICRNCGLTVE    90
             CE2180 DILEEIDNFASAKAIHAELNAREHSVGLTTVYRTLQSLAEIGAVDVLTVTGGETLYRQCHD--EGHHHHLVCTQCGRTEE   131
           NCgl2200 DVLEEIDNFASAKEIHHELSTREHNVGLTTVYRTLQSLADIGAVDVLTVTGGETLYRQCHD--EGHHHHLVCTNCGRTVE   104
            DIP1710 GVLKDLDYFASAKVIHQELTKRDLKVGLTTVYRTLQSLSEIEAVDVLHMSNGETLYRHCLS--DEHHHHLVCTECGRTVE   102
             jk0612 ELLEGLTTFSSAQDIHQKLVAQDHKVGLTTVYRTLQQMAEAGTIDTLYDDSGETLYRACAT--DDHHHHLVCTSCRRTVE   102
            PPA0948 ALFDDEPFFLTAQQVHDQLRNRGDQVGLATVYRNLQTMAEDGELDAIRAEDGEMTYRRCS---SAHHHHLVCRNCGKAVE    92
          FRAAL5117 EVLRAADDHPTAAEVYERVRRASPRIGSATVYRTLALLVSTGRALELNLGDGAAARYDAN---TRRHDHAVCDGCGRAVD    81
     Francci3_3112 AVLAAARDHPTAAEVHERVRTVSPGIGSATVYRTLALLVSAGRALELNLGDGTAARYDAN---TSRHDHVVCDGCRRAVD    94
Franean1DRAFT_4643 EVLKAAEDHPTAAEVYERVRLAAPGIGSATVYRALALLVETGHALELNLGDGAAARYDAN---TSRHDHAVCERCGRAAD    94
          FRAAL2798 RVLAGTSEHLSVDDVLTRADEIVPGLHRTTVYRALETLGELGLVTHVHPDHGPAVYHLASSLTGSGHLHVRCRRCGQISD   131
     Francci3_2661 QVLAGTSEHLSVDDVFARAETIVPGMHRTTVYRAVETLGDLGLVTHVHPDHGPAVYHLAAALTGGAHLHVRCRSCGTILD   124
          Rxyl_1144 AALRDAPGPLSAARIREAAEREAPGLGRATVYRALRTLREEGLVSVVELPGEEPLYESSG---RGEHHHFRCRGCGSILD    91
            SAV3053 EVLDGDHVHLTADEVHARAVVKLPEISRATVYNTLGELVSLGEVLEVATDKRAKRYDPNAHR---PHHHLVCARCGAIRD   100
            SCO5206 EVLDGEHVHLTADEVHSRAVDKLPEISRATVYNTLGELVSLGEVLEVATDKRAKRYDPNAHR---PHHHLVCARCGAIRD   100
          Acel_2095 QELPVGV-HRSVDEIATAVRRTLGAVSLQAVYNVLDALAGAGLVRRIEPLGHPARYESRVS---DNHHHLVCRVCGAIAD    99
          Noca_0839 AEVSEHP-HADVDTIATAARARLGKVSTQAVYDVVHALTDAGLLRRFQPAGSPARFELATG---DNHHHLVCRACHRIVD    99
          Mkms_4974 ------------------MREALGKVSTQAIYDVLRACVSAGLVRRIEPAGSSARYETRIG---DNHHHLVCRRCGRVAD    59
          Mmcs_4885 ------------------MREALGKVSTQAIYDVLRACVSAGLVRRIEPAGSSARYETRIG---DNHHHLVCRRCGRVAD    59
          Mjls_5253 SALEQAP-HSTADDVAKQVREALGKVSTQAIYDVLRACVSAGLVRRIEPAGSSARYETRIG---DNHHHLVCRRCGRVAD   104
         MSMEG_6253 EVLAEQP-HSTADDVAMAVRQNLGSVSTQAVYDVLRACVNAGLVRRIEPAGSSARYETRTG---DNHHHLVCRVCGRVAD   104
          FRAAL3168 DVLAEHP-HATADAVAERVRARLGTVSKQAIYDVLTVFVTAGLVRRIEPAGSPARFETRTA---DNHHHLVCRVCGRVAD   100
            nfa3250 KAVSAHP-HADADTIAAEVRAALGSVSTQGIYDVLHACVGAGLLRRIEPAGSPARYETRTG---DNHHHLVCRRCGAVAD   103
       RHA1_ro04308 NAVAVRP-HSDVDDIAAVVREELGSVSTQAVYDVLKACTAAGLLRRIEPAGSPARFETRTG---DNHHHLVCRSCGTVVD   102
           Tfu_0145 DAVRQGN-HLDADRVARAVRERVGHVSTQAVYDSLHALTAAGLLRRIEPAGSPARYETRVG---DNHHHLVCRACGEVTD    98
          Mjls_2712 EAVHAHP-HADTETIFATVRSSLPDVSRQAVYDVLHALTGARLIRRMQPSGSVARYETRVG---DNHHHVVCRSCGTIAD   105
          Mmcs_2681 EAVHAHP-HADTETIFATVRSSLPDVSRQAVYDVLHALTGARLIRRMQPSGSVARYETRVG---DNHHHVVCRSCGTIAD   143
          Mkms_2726 EAVHAHP-HADTETIFATVRSSLPDVSRQAVYDVLHALTGARLIRRMQPSGSVARYETRVG---DNHHHVVCRSCGTIAD   105
         MSMEG_3460 EAVHANP-HADTETIYSVVRGSLPTVSRQAVYDVLHALTAANLVRRIQPSGATARYEARVG---DNHHHVVCRNCGSIAD   102
           MAV_2752 EVVDANP-HADTETIFSAVRMALPDVSRQAVYDVLNALTAVGLVRRIQPLGMVARYESRVG---DNHHHVVCRSCGTIAD    90
          MAP1669c EVVDANP-HADTETIFSAVRMALPDVSRQAVYDVLNALTAVGLVRRIQPLGMVARYESRVG---DNHHHVVCRSCGTIAD   102
           MUL_2189 EAVGDHP-HADTETIYSAVREILPDVSRQAVYDVLGALTSVGLVRRIQPSGSVARYESRVG---DNHHHVVCRSCGAIAD    90
           Mb1944c EAVNAHP-HADTETIFGAVRFALPDVSRQAVYDVLHALTAAGLVRKIQPSGSVARYESRVG---DNHHHIVCRSCGVIAD   105
             MT1960 EAVNAHP-HADTETIFGAVRFALPDVSRQAVYDVLHALTAAGLVRKIQPSGSVARYESRVG---DNHHHIVCRSCGVIAD   105
           Rv1909c EAVNAHP-HADTETIFGAVRFALPDVSRQAVYDVLHALTAAGLVRKIQPSGSVARYESRVG---DNHHHIVCRSCGVIAD   105
          BCG_1948c EAVNAHP-HADTETIFGVVRFALPDVSRQAVYDVLHALTAAGLVRKIQPSGSVARYESRVG---DNHHHIVCRSCGVIAD   105
       RHA1_ro05274 EAVHARP-HADTETIFGAVRTVLEGVSRQAVYDVLHAVTAVGLVRRIQPPGSVARYESRVG---DNHHHVICRSCGAIAD    99
          Mvan_2983 EAVEAHP-HADTDTIFGAVRTALPEVSRQAVYDVLAALTGAGLVRKIQPSGSVARYETRVG---DNHHHVVCRSCGVITD   100
Franean1DRAFT_1681 TAVYAHP-HADTDSIVGSVREELPEVSHQAVYDSLRVLTTAGLVRRIQPAGSAARYESRVG---DNHHHVVCRSCRAIAD   123
          Noca_0874 GAVHALP-HADTESIIGAVREGLPGVSHQAVYDSLHALTAAGLVRRIQPAGSVARYEARVG---DNHHHVVCRACGIVAD   106
         MSMEG_6383 RSVYAHP-HADTDTVIRAVREALPEVSHQAVYDSLHALAAARLVRRIQPSGSVARYESRVG---DNHHHVVCRACGDIAD   102
          Mvan_3209 HAVGRHP-HADTELIINATRELVPDVSRQTVYDALNALAATGLVRRIQPAGSVARYETRVG---DNHHHVVCRNCGVIAD   105
           nfa29490 SVVHEHP-HSDTDSILGRVRESVGAVSHQAVYDVLRALTTAGLLRRIQPMGSVARYETRVD---DNHHHLVCRDCGVIVD   102
            SCO0561 ETVRAGD-HLDAEALTSGVRRRVGHVSLQAVYDALHALTAARLVRRIEPPGSPARFEGRVG---DNHHHVLCRSCGALAD   107
               FurS ETVRDGD-HLGVEAIASGVRDRVGHISLQAVYEALHALTAAGLVRRIEPAGHPARYEGRVG---DNHHHIVCRSCGAVAD   104
          AAur_3058 AAVQHHP-HSPAEGILAAARKELPELTAQSVYVVLSDLTDLQMLRRFEPPHSPALYETRVG---DNHHHAVCISCGKVED   106
          Arth_3077 TAVERHP-HSPAESILAAAREELPEMTAQSVYVVLGDLTDLHMLRRFEPPHSPALYETRVG---DNHHHAICISCGRVED   106
           Lxx02790 SALSDRP-HSDAETVLAAVQPALPGTSIQNIHNVLGDLTLTRMVRRIEPAHSPALYERRIG---DN-HHVVCTLCSTVAD   100
          Rxyl_1224 EALKEAEGHPSAEDVYLLVKRKNPRVALGTVYQALSVLEEIGVVESKHWSESPTRYDLNLEP----HLDIRCVRCGRVEE   109
          Mjls_1895 ALRETGLSMSQYAVLRALADHPQASAAELARLCFVTRQSLQDVSTGLRSAGLIDHAQVPAR----GRARALTLTATGRRR   102
              ruler ........90.......100.......110.......120.......130.......140.......150.......160
```

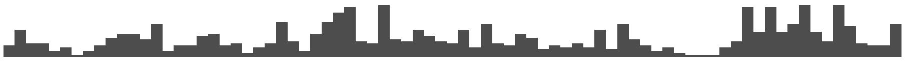

```
SAV4029            ADIG-VAAEFTAKLRETF---GFDTDMKHFAIFGRCQNCALKSSTTKS-----------------------   145
SCO4180            ADLS-VAADFTAKLREQF---GFDTDMKHFAIFGRCESCSLKGSTTDS-----------------------   145
Franean1DRAFT_3571 ISPE-RLAGIVGELRAEL---GFTVDMDHLTLAGRCAECTAAGRR--------------------------   144
Acel_0061          VGSE-VVQSLIQRLDDEH---GFAADVHHFAIFGRCSTCRSTT----------------------------   150
Noca_4251          VDPE-VIGPLAERLVRDH---GFVIDVGHLAVFGRCTDCEEDR----------------------------   139
Rxyl_1228          FNEE-LMEYLTLQVERET---GFVTDSYEITLHGLCRECAAAG----------------------------   138
Rxyl_1140          FEEC-PLDGAGLG--RIG---GFSPRAHSLEVYGRCAGCR-------------------------------   152
AAur_2630          VEAP-AVETWAARTATEH---GFTEVAHTVEIFGLCPECTAKKAAGKL-----------------------   168
Arth_2638          VEAP-AVETWAARTAAEH---GYTEVAHTVEIFGLCPDCTARKADAG------------------------   154
MAV_2036           VGDH-EVEEWATAVAAKH---GFSDVSHTIEIFGTCSECR-------------------------------   135
MAP2139            VGDH-EVEEWATAVAAKH---GFSDVSHTIEIFGTCSECR-------------------------------   135
Mb2380             VGDH-EVEAWAAEVATKH---GFSDVSHTIEIFGTCSDCRS------------------------------   130
BCG_2373           VGDH-EVEAWAAEVATKH---GFSDVSHTIEIFGTCSDCRS------------------------------   130
MT2428             VGDH-EVEAWAAEVATKH---GFSDVSHTIEIFGTCSDCRS------------------------------   130
Rv2359             VGDH-EVEAWAAEVATKH---GFSDVSHTIEIFGTCSDCRS------------------------------   130
MUL_3612           VADH-EVETWAANVAARH---GFSDVSHTIEIFGTCSDCRRPGAAALR-----------------------   137
ML0824             VGDH-EVEAWAAEVAAKH---GFSDVSHTIEIFGTCSECRS------------------------------   131
MSMEG_4487         VSGG-DVETWAAQVARDH---GFSDVSHTIEIFGVCGDCVERPAGNQT-----------------------   136
Mvan_3820          IQGG-QVETWAAEVAHEH---GFSDVSHTIEIFGICSNCA---AGN-------------------------   131
Mjls_3458          IQGG-QVETWAADIAREH---GFSDVSHTIEIFGLCGDCSAR-----------------------------   131
Mkms_3510          IQGG-QVETWAADIAREH---GFSDVSHTIEIFGLCGDCSAR-----------------------------   131
Mmcs_3447          IQGG-QVETWAADIAREH---GFSDVSHTIEIFGLCGDCSAR-----------------------------   131
nfa14570           VAGP-TVEAWADAIAGEH---GFTDVSHTIEVFGTCRDCAERRS---------------------------   141
RHA1_ro01222       VEGP-TVEQWSQSIADAH---GFSDVSHTIEIFGLCKDCSATQ----------------------------   141
SAV5631            VEGP-AVEKWAEAIAAEH---GYVNVAHTVEIFGTCAECAAAKS---------------------------   138
SCO2508            VEGP-AVEKWAEAIAAEH---GYVNVAHTVEIFGTCADCAGASGG--------------------------   139
Noca_1934          VEGP-AVERWTRAIAAEH---GYAEVSHTLEIFGTCARCG-------------------------------   135
FRAAL0074          IAGP-EIEAWTAAIADAE---GFVDVEHTVEIYGTCAGCAAASAAS-------------------------   131
Francci3_0061      IAGP-EIEAWTAKVANAE---GFVDVVHTVEIYGTCTICAARPAT--------------------------   142
Franean1DRAFT_4289 IAGP-EIEAWTSSVASAE---GFTDVVHTVEIYGTCARCAASRNSDNSPGRPRGGG---------------   192
Tfu_0856           IEGP-AVESWADELAAQH---GFVDLTHTLELFGTCSDCAAAKPRT-------------------------   129
Acel_2085          VKGI-SVERWAERVAARA---GFVDVDHTLEVVGTCPGCAAGRR---------------------------   138
BAD_0517           IES--PSESWLKGISDKY---GFTIERHTLEVFGLCEDCRKADK---------------------------   132
BL1128             IDP--PSEAWLRKVADGH---GFTVESHTLEVFGLCSDCQKEQKAQKAQKAVSE-----------------   146
Lxx25010           IEAD-EVEAWARSAAADH---GFSQVQHVVDVFGLCAGCS-AKADLAASSAVP------------------   138
CE2180             IDGG-PVERWAGEVARTH---GFSVSSHEAEIFGLCPACRPDTVDGQA-----------------------   175
NCgl2200           IDGG-PVETWAQEIATKN---GFALSSHEAEIFGLCADCKEKVT---------------------------   144
DIP1710            IDGG-PVEKWAKEVAQLH---GFQVTGHDAEIYGLCESCSAATE---------------------------   142
jk0612             IDGG-PVEDWAAETAKKF---GYQKSGHTAEIFGLCGDCQKKSAS--------------------------   143
PPA0948            IGPEKILEDWTRDIAAKY---EFSETGHELELFGICSECSAVHTRSVS-----------------------   137
FRAAL5117          IDHP-VPDGMMAEIARRS---GFAITGYDLQFRGMCPDCQANGSDPARAAGGTGPTVNTHAKGALHAEA--   146
Francci3_3112      IDHP-VLDGMMAQIACRS---GFAITGYDLRFRGLCPDCQANGSGPARGAAGTGQQ---------------   146
Franean1DRAFT_4643 IDHP-VPDGMVAEIARRS---GFTITGYDLQFRGLCPECQTAGEGHRPGRR--------------------   141
FRAAL2798          VPAD-LLDGVADRLARQI---GFRLQPDHAALTGLCRPCAAAADPPD---HPR------------------   177
Francci3_2661      VPAD-LLDDVARRLATTV---GFTLQPDHAALAGRCRACSVTSPSVDGDLAPA------------------   173
Rxyl_1144          LPGA-LLRVPAGTLLPG----GHRVEECSLLLSGSCGECLSREGRTR---GPGG-----------------   137
SAV3053            VHPS-GNPLADLPDSERF---GFTVSDVEVTYRGVCPNCAAA-----------------------------   138
SCO5206            VHPT-GNPLADLPDSERF---GFTVSDVEVTYRGTCPNCAAA-----------------------------   138
Acel_2095          VDCAVGVVPCLSPADAAG----YLVDEAEVTYWGLCPQCQNHRRPSADHGGTTAGPTEKHHVPARGEGGDR   166
Noca_0839          VACATGEAPCLQASDDAG----YLLDEAEVTYWGLCANCQ------------AAGELELL-----------   143
Mkms_4974          VDCIVGAAPCLDHAPLEDG---FTVDEAEVVFWGTCADCRA--------EPPAAGASCRL-----------   108
Mmcs_4885          VDCIVGAAPCLDHAPLEDG---FTVDEAEVVFWGTCADCRA--------EPPAAGASCRL-----------   108
Mjls_5253          VDCIVGAAPLLGHPSPRGR---FHRRRGRSRLLGHMRGLSR--------G--AAGGRGVLPSVTGLA----   158
MSMEG_6253         VDCAVGEAPCLEPSDLAG----FAIDEAEVVYWGVCADCQH--------DS-ATTTRPQSSTI--------   154
FRAAL3168          VDCVTGARPCLEPADDAG----FVIEEAEVVFWGVCRDCRP--------GDSGSDGVPAR-----------   148
nfa3250            VDCAVGAAPCLEPSETHG----YAVDEAEVVFWGLCPDCR---------AAQD------------------   143
RHA1_ro04308       VDCAVGEAPCLDPSEAHG----FEIDEAEVVFWGLCSQCRT--------GAQSGTGSAAALS---------   152
Tfu_0145           IDCVVGEAPCLAPSQAHG----YTVDEAEVVFWGVCPSCKA--------KESEH-----------------   140
Mjls_2712          VDCAVGEAPCLMPS-EHDALDGFVLDEAEVIYWGVCPDCAA--------ESSTADSSRSHP----------   157
Mmcs_2681          VDCAVGEAPCLMPS-EHDALDGFVLDEAEVIYWGVCPDCAA--------ESSTADSSRSHP----------   195
Mkms_2726          VDCAVGEAPCLMPS-EHDALDGFVLDEAEVIYWGVCPDCAA--------ESSTADSSRSHP----------   157
MSMEG_3460         VDCAVGEAPCLTPSNDDHALDGFLLDEAEVIYWGRCRECSV--------DGP-------------------   146
MAV_2752           VDCAVGEAPCLTPSDDDNVLDGFVLDEAEVIYWGLCADCST--------AGS-------------------   134
MAP1669c           VDCAVGEAPCLTPSDDDNVLDGFVLDEAEVIYWGLCAECST--------AGS-------------------   146
MUL_2189           IDCAVGEAPCLAPSDDSNVLDGFVVDEAEVIFWGMCPDCST--------AMPRSQP---------------   138
Mb1944c            VDCAVGEAPCLTASDHNG----FLLDEAEVIYWGLCPDCSI--------SDTS----RSHP----------   150
MT1960             VDCAVGEAPCLTASDHNG----FLLDEAEVIYWGLCPDCSI--------SDTS----RSHP----------   150
Rv1909c            VDCAVGEAPCLTASDHNG----FLLDEAEVIYWGLCPDCSI--------SDTS----RSHP----------   150
BCG_1948c          VDCAVGEAPCLTASDHNG----FLLDEAEVIYWGLCPDCSI--------SDTS----RSHP----------   150
RHA1_ro05274       VDCAVGEAPCLTASDDNG----FLVEEAEVIYWGLCPDCST--------SQTS----RSNP----------   144
Mvan_2983          VDCAVGEAPCLVPSDPDGSLDGFVVDEAEVVYWGLCPGCLA--------AEIS----RSHQ----------   149
Franean1DRAFT_1681 VDCAVGSAPCLAASDDRG----FSIDEAEVFYWGRCPDCSPPA----TPDRPRAAPDRS------------   174
Noca_0874          VDCAVGGAPCLTASDDHG----FRIDEAEVVYWGICPACS---------------TTRS------------   146
MSMEG_6383         VDCAVGEAPCLTGSDHHG----YAIDEAEVIYWGTCPACAQ------DPDRAKARTSRSHP----------   153
Mvan_3209          VDCAVGEAPCLTASDDLG----FEVDEAEVIYWGICPACST--------------APSPQ-----------   147
nfa29490           VDCAVGEAPCLDASHDHG----FVVDEAEVIYWGHCPDCSK--------ALSAPPK---------------   146
SCO0561            VDCAVGDAPCLTASDDRG----FVIDEAEVIYRGLCPDCST--------PGSSPAP---------------   151
FurS               VDCAVGDAPCLTAADDHG----FAIDEAEVVYWGLCPDCST--------GRSS------------------   145
AAur_3058          VDCAVGHAPCLTPHWDENSKP-MTIQIADVLYQGICQDCQS---------KQQLPVTSVTQK---------   158
Arth_3077          VDCAVGHAPCLTPHWDENSEP-MTIQIADVMYQGICQDCQR---------TQKLPAERN------------   155
Lxx02790           VDCVVGHAPCLR--LSEAAG--FAVDTAEVTFWRLCPSCQE---------STAATPSRSE-----------   147
Rxyl_1224          VPGVDAGDLESRIQENTP----YRVTRATMLLEGVCPACQDG-----------------------------   147
Mjls_1895          LAAAHSAVTAVEEQMLTG-----LSGAECRRLSGLLAACAANLEGE-------------------------   143
ruler              .......170.......180.......190.......200.......210.......220.......230.
```

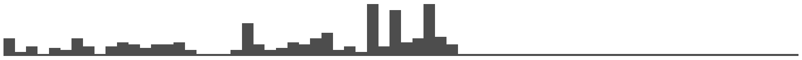

Supplement: Additional File 1 — Multiple alignment of the studied Fur homologues + Streptomyces reticuli FurS. [file 1471-2148-8-185-S1.pdf]
