# Supplementary material for: On the nature of fur evolution: A phylogenetic approach in Actinobacteria
Source: BMC Evol Biol. 2008 Jun 25;8:185. doi: 10.1186/1471-2148-8-185 (PMC2464607; doi:10.1186/1471-2148-8-185)

```
SAV4029             ----------------------------------------MVS--TDWKNDLRQRGYRLTPQRQLVLEAVD-KL-EHATP   36
SCO4180             ----------------------------------------MVS--TDWKSDLRQRGYRLTPQRQLVLEAVD-TL-EHATP   36
Franean1DRAFT_3571  ----------------------------------------MAAKVDDWHTRLRARGYRLTPQRQLVLEAVA-RL-GHATP   38
Acel_0061           --------------------------------MPKGSSDRPSTRSAQWQGRLRSKGYRLTPQRRLVLEAVA-AL-RHATP   46
Noca_4251           -----------------------------------------MT--EELAARLREQGYRLTPQRQLVLQAVE-KL-GHATP   35
Acel_2095           --------------------------------------------MRSPVDRLRAAGMRVTPARLAVLQELP-VG-VHRSV   34
Noca_0839           --------------------------------------------MAPAEEDLRAAGLRVTRPRVAVLAEVS-EH-PHADV   34
Mkms_4974           --------------------------------------------------------------------------------
Mmcs_4885           --------------------------------------------------------------------------------
Mjls_5253           ---------------------------------------MTPARETTPEARLRASGLRVTAPRLAVLSALE-QA-PHSTA   39
MSMEG_6253          ---------------------------------------MTTVHDHDPKALLRASGLRVTAPRVAVLEVLA-EQ-PHSTA   39
FRAAL3168           -------------------------------------------MATDRATTLRGAGLRVTGPRLAVLDVLA-EH-PHATA   35
nfa3250             ----------------------------------------MHTETAEPRDRLRAAGLRVTAPRLAVLKAVS-AH-PHADA   38
RHA1_ro04308        ----------------------------------------MRHDT-DPKAQLREVGLRVTAPRVAVLNAVA-VR-PHSDV   37
Tfu_0145            ---------------------------------------------MDDAASLRAAGLRVTAARLAILDAVR-QG-NHLDA   33
MAV_2752            -----------------------------------------------------MADLRVTRPRVAVLEVVD-AN-PHADT   25
MAP1669c            -----------------------------------------MSSTADYADRLRMADLRVTRPRVAVLEVVD-AN-PHADT   37
MUL_2189            -----------------------------------------------------MADLRVTRPRLAVLEAVG-DH-PHADT   25
Mjls_2712           --------------------------------------MAVVSSSSEYVDQLRGADLRVTRPRVAVLEAVH-AH-PHADT   40
Mmcs_2681           MITPLGLKSRICLARRPIGVSAQNARDELLTYSKKAGDMAVVSSSSEYVDQLRGADLRVTRPRVAVLEAVH-AH-PHADT   78
Mkms_2726           --------------------------------------MAVVSSSSEYVDQLRGADLRVTRPRVAVLEAVH-AH-PHADT   40
MSMEG_3460          -----------------------------------------MPSRAEFEAQLRMTDLRVTRPRIAVMEAVH-AN-PHADT   37
Mb1944c             --------------------------------------MSSVSSIPDYAEQLRTADLRVTRPRVAVLEAVN-AH-PHADT   40
MT1960              --------------------------------------MSSVSSIPDYAEQLRTADLRVTRPRVAVLEAVN-AH-PHADT   40
Rv1909c             --------------------------------------MSSVSSIPDYAEQLRTADLRVTRPRVAVLEAVN-AH-PHADT   40
BCG_1948c           --------------------------------------MSSVSSIPDYAEQLRTADLRVTRPRVAVLEAVN-AH-PHADT   40
RHA1_ro05274        --------------------------------------------MPDYAALLRGAELRVTRPRVAVLEAVH-AR-PHADT   34
Mvan_2983           -------------------------------------------MTSDYAELLRGADLRVTRPRIAVLEAVE-AH-PHADT   35
Franean1DRAFT_1681  --------------------MVAWLLSGFLDWFQKMGDHVVVATAEDFRRMLRGAALRVTRPRVAVLTAVY-AH-PHADT   58
Noca_0874           -------------------------------------MATTTDRTRDFPELLRGAALRVTRPRLAVLGAVH-AL-PHADT   41
MSMEG_6383          -----------------------------------------MTHTTDFEQLLRSAGMRVTRPRIAVLRSVY-AH-PHADT   37
Mvan_3209           --------------------------------------MEPMTEEPDYSAVLRSAALRVTRPRMAVLHAVG-RH-PHADT   40
nfa29490            -----------------------------------------MSKVPDFEQLLRGASLRVTAQRLAVLSVVH-EH-PHSDT   37
SCO0561             ------------------------------------MTASRNSRKPATADELRRAGLRVTAARVALLETVR-AG-DHLDA   42
AAur_3058           -------------------------------------MTDHTTEQAEWAAALHAHGRRVTKQRLAVLAAVQ-HH-PHSPA   41
Arth_3077           -------------------------------------MTVHEAGQDAWAAALRAHGRRVTKQRLAVLTAVE-RH-PHSPA   41
Lxx02790            ------------------------------------------MDTAQLEDTLRSAGLRVTRGRLAVLSALS-DR-PHSDA   36
SAV3053             --------------------------------------------MSDLLERLRGRGWRMTAQRRVVAEVLDGDH-VHLTA   35
SCO5206             --------------------------------------------MSDLLERLRGRGWRMTAQRRVVAEVLDGEH-VHLTA   35
FRAAL2798           ----------------MEDRRPVGAAAATAAAGHDALPGRRDDLAAAGIAELRALGERVTPARRAVLRVLAGTS-EHLSV   63
Francci3_2661       ----------------MKDTWIVKDSQTDRD-------GRRGDPSAAGIAALRARGERVTPARRAVLQVLAGTS-EHLSV   56
Rxyl_1144           -----------------------------------------------------MSFRRRTRQREAILAALRDAP-GPLSA   26
FRAAL0074           --------------------------------------------------------MRATRQGDAVCAALTETD-AFTSA   23
Francci3_0061       -----------------------------------------MERRPAPSRGT---AVRATRQGDAVSAALAETD-AFTSA   35
Franean1DRAFT_4289  --MDVVASVEGERVSSPRGMSAASDRAAGGAGAGSGDRPNGAVSRPGRGRGT---SVRATRQGDAVSSALAATN-AFTSA   74
Tfu_0856            --------------------------------------------------------MSSRR--EAVRQALHKSN-GFRSA   21
MAV_2036            -----------------------------------------MTPSGDG----AGVSVRSTRQRAAISTLLETVD-DFRSA   34
MAP2139             -----------------------------------------MTPSGDG----AGVSVRSTRQRAAISTLLETLD-DFRSA   34
Mb2380              -----------------------------------------MSAAG----------VRSTRQRAAISTLLETLD-DFRSA   28
BCG_2373            -----------------------------------------MSAAG----------VRSTRQRAAISTLLETLD-DFRSA   28
MT2428              -----------------------------------------MSAAG----------VRSTRQRAAISTLLETLD-DFRSA   28
Rv2359              -----------------------------------------MSAAG----------VRSTRQRAAISTLLETLD-DFRSA   28
ML0824              ----------------------------------------MMTGAS----------VRSTRQRAAISTLLETVD-DFRSA   29
MUL_3612            -----------------------------------------MTGTS----------VRSTRQRAAISTLLETVD-DFRSA   28
MSMEG_4487          -----------------------------------------MTGAV-----------RSTRQRAAIADLLNETE-GFRSA   27
Mvan_3820           -----------------------------------------MTGAV-----------RSTRQRAAISALLENIE-DFRSA   27
Mjls_3458           -----------------------------------------MSPAP----------VRATRQRAAIADLLDGLD-EFRSA   28
Mkms_3510           -----------------------------------------MSPAP----------VRATRQRAAIADLLDGLD-EFRSA   28
Mmcs_3447           -----------------------------------------MSPAP----------VRATRQRAAIADLLDGLD-EFRSA   28
nfa14570            -----------------------------------------MQDKTSAPQKP--VGIRSTRQRSAIAALLGDID-EFRSA   36
RHA1_ro01222        -----------------------------------------MTQNTGQ-RKPVVVGVRATKQRSAISALLDDIE-EFRSA   37
SAV5631             -----------------------------------------MTTAGSP------VRGRSTRQRAAVAAALDEVD-EFRSA   32
SCO2508             -----------------------------------------MTTAGPP------VKGRATRQRAAVSAALQEVE-EFRSA   32
Noca_1934           -----------------------------------------MTSTEHAETP----RLRPTRQRRAVTEALDSFS-DFRSA   34
Acel_2085           -----------------------------------------MSGSSEG------RQSRSTRQKRALAQVMAESR-QFRSA   32
BAD_0517            -----------------------------------------MSEHI----------VRQTRQKDAIRDALNGCD-EFISA   28
BL1128              -------------------------------------MTLPMVEHI----------ERQTKQKDAIRAALADCE-EFISA   32
Lxx25010            --------------------------------------------MV----------KRNTWQREAVREALSSTE-GFISA   25
AAur_2630           ---------------------MGFASIPREVSLSMPQGTSSATTGNEAPATSGVKEQRVTKQRLAVSAALDELD-DFVST   58
Arth_2638           ----------------------------------MAHGAKAEGTKLSAPASAGGREQRVTKQRLAVSAALDELD-DFVST   45
PPA0948             -----------------------------------------MENRR-----------RTTKQRLAIRALFDDEP-FFLTA   27
CE2180              ------------MLKKPSRPLRSRIRGNNHCFPDSQEGGMGLNRHGEK--STPKLGVRSTRQRKAVVDILEEID-NFASA   65
NCgl2200            ---------------------------------------MGINRISQG--SAPKLGVRSTRQRKAVIDVLEEID-NFASA   38
DIP1710             -----------------------------------------MNRTIDR--SVPKLGVRSTRQRTAVVGVLKDLD-YFASA   36
jk0612              -----------------------------------------MSNPATN--KLPKIGQRNTRQRRAVAELLEGLT-TFSSA   36
FRAAL5117           ---------------------------------------------------------------MAVLEVLRAAD-DHPTA   16
Francci3_3112       -----------------------------------------MNDQT---------DLRLTPQRMAVLAVLAAAR-DHPTA   29
Franean1DRAFT_4643  -----------------------------------------MPERT---------ELRLTPQRTRVLEVLKAAE-DHPTA   29
Rxyl_1228           --------------------------------------------MMEFEQILRSRGYRLTNQRRVIVRELE-GE-RHLSA   34
Rxyl_1140           -------------------MTPDPPPGSLSRVVEKFFQLGGDLELEAVLGRMRGRGYKVTPQRLAVLEALA------AER   55
outgroup            ------------------------------------------MWKERAMEEMKKAGLRLTPQRLKLIEVIEKIGGRHPTL   38
ruler               1.......10........20........30........40........50........60........70........80
```

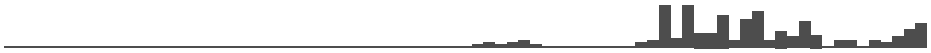

```
                                  :  :                                   :      .
SAV4029              DDILVEVRKTASGVNISTVYRTLELLEELGLVSHAHLGHGAPTYHLAD---RHHHIHLVCRDCTNVIEADIG-VAAEFTA   112
SCO4180              DDILGEVRKTASGINISTVYRTLELLEELGLVSHAHLGHGAPTYHLAD---RHHHIHLVCRDCTNVIEADLS-VAADFTA   112
Franean1DRAFT_3571   EAIVHEVRQTASGVNISTVYRTLDLLEELDLVSHAHLSHGAPTYHVTG---VDPHVHLVCGACGAILEISPE-RLAGIVG   114
Acel_0061            EEIINYVREVASGVNMSTVYRTLDLLEELSLVTHTHLGHGAPTYHVAE---DAGHVHLVCSSCGAVTEVGSE-VVQSLIQ   122
Noca_4251            DEVLAEVHAQSSAVNVSTIYRTLEVLEELGLVRHAHLSDRAPTYHSVT---DREHFHLVCRNCHKVVSVDPE-VIGPLAE   111
Acel_2095            DEIATAVRRTLGAVSLQAVYNVLDALAGAGLVRRIEPLGHPARYESRV---SDNHHHLVCRVCGAIADVDCA-VGVVPCL   110
Noca_0839            DTIATAARARLGKVSTQAVYDVVHALTDAGLLRRFQPAGSPARFELAT---GDNHHHLVCRACHRIVDVACA-TGEAPCL   110
Mkms_4974            ------MREALGKVSTQAIYDVLRACVSAGLVRRIEPAGSSARYETRI---GDNHHHLVCRRCGRVADVDCI-VGAAPCL    70
Mmcs_4885            ------MREALGKVSTQAIYDVLRACVSAGLVRRIEPAGSSARYETRI---GDNHHHLVCRRCGRVADVDCI-VGAAPCL    70
Mjls_5253            DDVAKQVREALGKVSTQAIYDVLRACVSAGLVRRIEPAGSSARYETRI---GDNHHHLVCRRCGRVADVDCI-VGAAPLL   115
MSMEG_6253           DDVAMAVRQNLGSVSTQAVYDVLRACVNAGLVRRIEPAGSSARYETRT---GDNHHHLVCRVCGRVADVDCA-VGEAPCL   115
FRAAL3168            DAVAERVRARLGTVSKQAIYDVLTVFVTAGLVRRIEPAGSPARFETRT---ADNHHHLVCRVCGRVADVDCV-TGARPCL   111
nfa3250              DTIAAEVRAALGSVSTQGIYDVLHACVGAGLLRRIEPAGSPARYETRT---GDNHHHLVCRRCGAVADVDCA-VGAAPCL   114
RHA1_ro04308         DDIAAVVREELGSVSTQAVYDVLKACTAAGLLRRIEPAGSPARFETRT---GDNHHHLVCRSCGTVVDVDCA-VGEAPCL   113
Tfu_0145             DRVARAVRERVGHVSTQAVYDSLHALTAAGLLRRIEPAGSPARYETRV---GDNHHHLVCRACGEVTDIDCV-VGEAPCL   109
MAV_2752             ETIFSAVRMALPDVSRQAVYDVLNALTAVGLVRRIQPLGMVARYESRV---GDNHHHVVCRSCGTIADVDCA-VGEAPCL   101
MAP1669c             ETIFSAVRMALPDVSRQAVYDVLNALTAVGLVRRIQPLGMVARYESRV---GDNHHHVVCRSCGTIADVDCA-VGEAPCL   113
MUL_2189             ETIYSAVREILPDVSRQAVYDVLGALTSVGLVRRIQPSGSVARYESRV---GDNHHHVVCRSCGAIADIDCA-VGEAPCL   101
Mjls_2712            ETIFATVRSSLPDVSRQAVYDVLHALTGARLIRRMQPSGSVARYETRV---GDNHHHVVCRSCGTIADVDCA-VGEAPCL   116
Mmcs_2681            ETIFATVRSSLPDVSRQAVYDVLHALTGARLIRRMQPSGSVARYETRV---GDNHHHVVCRSCGTIADVDCA-VGEAPCL   154
Mkms_2726            ETIFATVRSSLPDVSRQAVYDVLHALTGARLIRRMQPSGSVARYETRV---GDNHHHVVCRSCGTIADVDCA-VGEAPCL   116
MSMEG_3460           ETIYSVVRGSLPTVSRQAVYDVLHALTAANLVRRIQPSGATARYEARV---GDNHHHVVCRNCGSIADVDCA-VGEAPCL   113
Mb1944c              ETIFGAVRFALPDVSRQAVYDVLHALTAAGLVRKIQPSGSVARYESRV---GDNHHHIVCRSCGVIADVDCA-VGEAPCL   116
MT1960               ETIFGAVRFALPDVSRQAVYDVLHALTAAGLVRKIQPSGSVARYESRV---GDNHHHIVCRSCGVIADVDCA-VGEAPCL   116
Rv1909c              ETIFGAVRFALPDVSRQAVYDVLHALTAAGLVRKIQPSGSVARYESRV---GDNHHHIVCRSCGVIADVDCA-VGEAPCL   116
BCG_1948c            ETIFGVVRFALPDVSRQAVYDVLHALTAAGLVRKIQPSGSVARYESRV---GDNHHHIVCRSCGVIADVDCA-VGEAPCL   116
RHA1_ro05274         ETIFGAVRTVLEGVSRQAVYDVLHAVTAVGLVRRIQPPGSVARYESRV---GDNHHHVICRSCGAIADVDCA-VGEAPCL   110
Mvan_2983            DTIFGAVRTALPEVSRQAVYDVLAALTGAGLVRKIQPSGSVARYETRV---GDNHHHVVCRSCGVITDVDCA-VGEAPCL   111
Franean1DRAFT_1681   DSIVGSVREELPEVSHQAVYDSLRVLTTAGLVRRIQPAGSAARYESRV---GDNHHHVVCRSCRAIADVDCA-VGSAPCL   134
Noca_0874            ESIIGAVREGLPGVSHQAVYDSLHALTAAGLVRRIQPAGSVARYEARV---GDNHHHVVCRACGIVADVDCA-VGGAPCL   117
MSMEG_6383           DTVIRAVREALPEVSHQAVYDSLHALAAARLVRRIQPSGSVARYESRV---GDNHHHVVCRACGDIADVDCA-VGEAPCL   113
Mvan_3209            ELIINATRELVPDVSRQTVYDALNALAATGLVRRIQPAGSVARYETRV---GDNHHHVVCRNCGVIADVDCA-VGEAPCL   116
nfa29490             DSILGRVRESVGAVSHQAVYDVLRALTTAGLLRRIQPMGSVARYETRV---DDNHHHLVCRDCGVIVDVDCA-VGEAPCL   113
SCO0561              EALTSGVRRRVGHVSLQAVYDALHALTAARLVRRIEPPGSPARFEGRV---GDNHHHVLCRSCGALADVDCA-VGDAPCL   118
AAur_3058            EGILAAARKELPELTAQSVYVVLSDLTDLQMLRRFEPPHSPALYETRV---GDNHHHAVCISCGKVEDVDCA-VGHAPCL   117
Arth_3077            ESILAAAREELPEMTAQSVYVVLGDLTDLHMLRRFEPPHSPALYETRV---GDNHHHAICISCGRVEDVDCA-VGHAPCL   117
Lxx02790             ETVLAAVQPALPGTSIQNIHNVLGDLTLTRMVRRIEPAHSPALYERRI---GDN-HHVVCTLCSTVADVDCV-VGHAPCL   111
SAV3053              DEVHARAVVKLPEISRATVYNTLGELVSLGEVLEVATDKRAKRYDPNA---HRPHHHLVCARCGAIRDVHPS-GNPLADL   111
SCO5206              DEVHSRAVDKLPEISRATVYNTLGELVSLGEVLEVATDKRAKRYDPNA---HRPHHHLVCARCGAIRDVHPT-GNPLADL   111
FRAAL2798            DDVLTRADEIVPGLHRTTVYRALETLGELGLVTHVHPDHGPAVYHLASSLTGSGHLHVRCRRCGQISDVPAD-LLDGVAD   142
Francci3_2661        DDVFARAETIVPGMHRTTVYRAVETLGDLGLVTHVHPDHGPAVYHLAAALTGGAHLHVRCRSCGTILDVPAD-LLDDVAR   135
Rxyl_1144            ARIREAAEREAPGLGRATVYRALRTLREEGLVSVVELPGEEPLYESSG---RGEHHHFRCRGCGSILDLPGA-LLRVPAG   102
FRAAL0074            QELHALLRSRGAGVGLTTVYRHLQVLVDRGEVDMIRRDDGETVYRRCAT--EAHHHHLVCRLCGRTVEIAGP-EIEAWTA   100
Francci3_0061        QDLHARLRAQGAAVGLTTVYRHLQVLADRGEVDMIRRDDGETVYRRCAS--DDHHHHLVCRSCGRTVEIAGP-EIEAWTA   112
Franean1DRAFT_4289   QDLHARLRQEGQSVGLTTVYRHLQVLADRGEVDVLRRDDGETVYRRCTT--DGHHHHLVCRHCGHTVEIAGP-EIEAWTS   151
Tfu_0856             QDLYASLRADGAKIGLTTVYRALQALTDAGEVDVLMTDEGEAVYRACST--PTHHHHLVCRDCGKAVEIEGP-AVESWAD    98
MAV_2036             QELHDELRRRGENIGLTTVYRTLQSMAAAGLIDTLRTDTGESVYRRCS---EHHHHHLVCRSCGSTIEVGDH-EVEEWAT   110
MAP2139              QELHDELRRRGENIGLTTVYRTLQSMAAAGLIDTLRTDTGESVYRRCS---EHHHHHLVCRSCGSTIEVGDH-EVEEWAT   110
Mb2380               QELHDELRRRGENIGLTTVYRTLQSMASSGLVDTLRTDTGESVYRRCS---EHHHHHLVCRSCGSTIEVGDH-EVEAWAA   104
BCG_2373             QELHDELRRRGENIGLTTVYRTLQSMASSGLVDTLRTDTGESVYRRCS---EHHHHHLVCRSCGSTIEVGDH-EVEAWAA   104
MT2428               QELHDELRRRGENIGLTTVYRTLQSMASSGLVDTLHTDTGESVYRRCS---EHHHHHLVCRSCGSTIEVGDH-EVEAWAA   104
Rv2359               QELHDELRRRGENIGLTTVYRTLQSMASSGLVDTLHTDTGESVYRRCS---EHHHHHLVCRSCGSTIEVGDH-EVEAWAA   104
ML0824               QELHNELRRRGNNISLTTVYRTLQSMAEAGMVDTLRTNTGESVYRRCS---RQHHHHLVCRGCGSTIEVGDH-EVEAWAA   105
MUL_3612             QELHDELRRRGENIGLTTVYRTLQSMASAGTVDTLRTDTGESVYRRCS---QHHHHHLVCRSCGSTIEVADH-EVETWAA   104
MSMEG_4487           QELHDELRRRGEGIGLTTVYRTLQAMATAGVVDTLRTDTGESVYRRCS---EHHHHHLVCRSCGSTVEVSGG-DVETWAA   103
Mvan_3820            QELHDELRRRGEGIGLTTVYRTLQQMAAAGVVDTLRTDTGESVYRRCS---EHHHHHLVCRACGCTVEIQGG-QVETWAA   103
Mjls_3458            QELHDALKRRGEGIGLTTVYRTLQSMAESGLVDVLRTDTGESVYRRCS---ADHHHHLVCRACGAAVEIQGG-QVETWAA   104
Mkms_3510            QELHDALKRRGEGIGLTTVYRTLQSMAESGLVDVLRTDTGESVYRRCS---ADHHHHLVCRACGAAVEIQGG-QVETWAA   104
Mmcs_3447            QELHDALKRRGEGIGLTTVYRTLQSMAESGLVDVLRTDTGESVYRRCS---ADHHHHLVCRACGAAVEIQGG-QVETWAA   104
nfa14570             QELHDELRKRGEGIGLTTVYRTLQSLADAGMVDVLRTDSGESVYRQCS---SGHHHHLVCRHCGRTVEVAGP-TVEAWAD   112
RHA1_ro01222         QELHDELRKRGEGIGLTTVYRTLQTLADAGTVDVLRTDTGESVYRRCS---SGHHHHLVCRTCGYTVEVEGP-TVEQWSQ   113
SAV5631              QELHDMLKHKGDSVGLTTVYRTLQNLADAGEVDVLRTSDGESVYRRCSS--GEHHHHLVCRVCGKAVEVEGP-AVEKWAE   109
SCO2508              QELHDMLKHKGDAVGLTTVYRTLQSLADAGEVDVLRTAEGESVYRRCST--GDHHHHLVCRACGKAVEVEGP-AVEKWAE   109
Noca_1934            QEIHELLGRRGETVGLATVYRTLQRLAEAGDVDVLRTEDGEAIYRRCS---EAHHHHLVCRSCGATVEVEGP-AVERWTR   110
Acel_2085            QELHEALIDRGESVGLTTVYNQLRAMAEAGLVDTLRAPDGETLYRQCET--NAHHHHLRCRYCGRTVEVKGI-SVERWAE   109
BAD_0517             QELHRKLEDRGEHIGLATVYRQLNALAEAGRADTVR-LDGQQLFRLCGD--DGHHHHLVCTNCGKTVEIES--PSESWLK   103
BL1128               QDLHRRLEDEGSKIGLATVYRQLNALADAGAADTIR-LDGQQLFRLCGD--DGHHHHLVCRRCGKTVEIDP--PSEAWLR   107
Lxx25010             QGLHLRLHEAGSPIGLATVYRALADLAAEGDADSLQSPEGESLYRACTS---GHHHHLICRNCGLTVEIEAD-EVEAWAR   101
AAur_2630            QELYRLLQNKGISVSLATAYRILQSLADDGLIDVLRNGDGEAVYRRCAV--TGHHHHLLCRNCGKAEEVEAP-AVETWAA   135
Arth_2638            QELYRMLQNQGTSVSLATAYRILQSLADDGLVDVLRNGEGEAVYRRCAV--TGHHHHLLCRNCGKAVEVEAP-AVETWAA   122
PPA0948              QQVHDQLRNRGDQVGLATVYRNLQTMAEDGELDAIRAEDGEMTYRRCS---SAHHHHLVCRNCGKAVEIGPEKILEDWTR   104
CE2180               KAIHAELNAREHSVGLTTVYRTLQSLAEIGAVDVLTVTGGETLYRQCHD--EGHHHHLVCTQCGRTEEIDGG-PVERWAG   142
NCgl2200             KEIHHELSTREHNVGLTTVYRTLQSLADIGAVDVLTVTGGETLYRQCHD--EGHHHHLVCTNCGRTVEIDGG-PVETWAQ   115
DIP1710              KVIHQELTKRDLKVGLTTVYRTLQSLSEIEAVDVLHMSNGETLYRHCLS--DEHHHHLVCTECGRTVEIDGG-PVEKWAK   113
jk0612               QDIHQKLVAQDHKVGLTTVYRTLQQMAEAGTIDTLYDDSGETLYRACAT--DDHHHHLVCTSCRRTVEIDGG-PVEDWAA   113
FRAAL5117            AEVYERVRRASPRIGSATVYRTLALLVSTGRALELNLGDGAAARYDAN---TRRHDHAVCDGCGRAVDIDHP-VPDGMMA    92
Francci3_3112        AEVHERVRTVSPGIGSATVYRTLALLVSAGRALELNLGDGTAARYDAN---TSRHDHVVCDGCRRAVDIDHP-VLDGMMA   105
Franean1DRAFT_4643   AEVYERVRLAAPGIGSATVYRALALLVETGHALELNLGDGAAARYDAN---TSRHDHAVCERCGRAADIDHP-VPDGMVA   105
Rxyl_1228            EEIYERVKEEHPELGLSTVYRTLDLLHELGIVRKEDFGEGYSRYELAT---ERMHHHARCRGCGKVIEFNEE-LMEYLTL   110
Rxyl_1140            HQSLEELRGRCPEVGLATLYRTLDLLTGIGAVRRLELGDGP-RYELAG----DHHHHLICEACGEISEFEEC---PLDGA   127
outgroup             KEVYEEVVKEFPTMSFSTLYSNLLIFRGLGLLDFFTLEGETRVEVNCEP-----HFNVIER-----EEIRDF-VDEELIG   107
ruler                ........90.......100.......110.......120.......130.......140.......150.......160
```

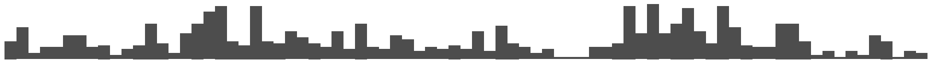

```
           SAV4029 KLRETFG----FDTDMKHFAIFGRCQNCALKSSTTKS-----------------------  145
           SCO4180 KLREQFG----FDTDMKHFAIFGRCESCSLKGSTTDS-----------------------  145
Franean1DRAFT_3571 ELRAELG----FTVDMDHLTLAGRCAECTAAGRR--------------------------  144
         Acel_0061 RLDDEHG----FAADVHHFAIFGRCSTCRSTT----------------------------  150
         Noca_4251 RLVRDHG----FVIDVGHLAVFGRCTDCEEDR----------------------------  139
         Acel_2095 SPADAAG----YLVDEAEVTYWGLCPQCQNHRRPSADHGGTTAGPTEKHHVPARGEGGDR  166
         Noca_0839 QASDDAG----YLLDEAEVTYWGLCANCQ------------AAGELELL-----------  143
         Mkms_4974 DHAPLEDG---FTVDEAEVVFWGTCADCRA--------EPPAAGASCRL-----------  108
         Mmcs_4885 DHAPLEDG---FTVDEAEVVFWGTCADCRA--------EPPAAGASCRL-----------  108
         Mjls_5253 GHPSPRGR---FHRRRGRSRLLGHMRGLSR--------G--AAGGRGVLPSVTGLA----  158
        MSMEG_6253 EPSDLAG----FAIDEAEVVYWGVCADCQH--------DS-ATTTRPQSSTI--------  154
         FRAAL3168 EPADDAG----FVIEEAEVVFWGVCRDCRP--------GDSGSDGVPAR-----------  148
           nfa3250 EPSETHG----YAVDEAEVVFWGLCPDCR---------AAQD------------------  143
      RHA1_ro04308 DPSEAHG----FEIDEAEVVFWGLCSQCRT--------GAQSGTGSAAALS---------  152
          Tfu_0145 APSQAHG----YTVDEAEVVFWGVCPSCKA--------KESEH-----------------  140
          MAV_2752 TPSDDDNVLDGFVLDEAEVIYWGLCADCST--------AGS-------------------  134
          MAP1669c TPSDDDNVLDGFVLDEAEVIYWGLCAECST--------AGS-------------------  146
          MUL_2189 APSDDSNVLDGFVVDEAEVIFWGMCPDCST--------AMPRSQP---------------  138
         Mjls_2712 MPS-EHDALDGFVLDEAEVIYWGVCPDCAA--------ESSTADSSRSHP----------  157
         Mmcs_2681 MPS-EHDALDGFVLDEAEVIYWGVCPDCAA--------ESSTADSSRSHP----------  195
         Mkms_2726 MPS-EHDALDGFVLDEAEVIYWGVCPDCAA--------ESSTADSSRSHP----------  157
        MSMEG_3460 TPSNDDHALDGFLLDEAEVIYWGRCRECSV--------DGP-------------------  146
           Mb1944c TASDHNG----FLLDEAEVIYWGLCPDCSI--------SDTS----RSHP----------  150
            MT1960 TASDHNG----FLLDEAEVIYWGLCPDCSI--------SDTS----RSHP----------  150
           Rv1909c TASDHNG----FLLDEAEVIYWGLCPDCSI--------SDTS----RSHP----------  150
         BCG_1948c TASDHNG----FLLDEAEVIYWGLCPDCSI--------SDTS----RSHP----------  150
      RHA1_ro05274 TASDDNG----FLVEEAEVIYWGLCPDCST--------SQTS----RSNP----------  144
         Mvan_2983 VPSDPDGSLDGFVVDEAEVVYWGLCPGCLA--------AEIS----RSHQ----------  149
Franean1DRAFT_1681 AASDDRG----FSIDEAEVFYWGRCPDCSPPA----TPDRPRAAPDRS------------  174
         Noca_0874 TASDDHG----FRIDEAEVVYWGICPACS---------------TTRS------------  146
        MSMEG_6383 TGSDHHG----YAIDEAEVIYWGTCPACAQ------DPDRAKARTSRSHP----------  153
         Mvan_3209 TASDDLG----FEVDEAEVIYWGICPACST-------------APSPQ------------  147
          nfa29490 DASHDHG----FVVDEAEVIYWGHCPDCSK--------ALSAPPK---------------  146
           SCO0561 TASDDRG----FVIDEAEVIYRGLCPDCST--------PGSSPAP---------------  151
         AAur_3058 TPHWDENSKP-MTIQIADVLYQGICQDCQS---------KQQLPVTSVTQK---------  158
         Arth_3077 TPHWDENSEP-MTIQIADVMYQGICQDCQR---------TQKLPAERN------------  155
          Lxx02790 R--LSEAAG--FAVDTAEVTFWRLCPSCQE---------STAATPSRSE-----------  147
           SAV3053 PDSERFG----FTVSDVEVTYRGVCPNCAAA-----------------------------  138
           SCO5206 PDSERFG----FTVSDVEVTYRGTCPNCAAA-----------------------------  138
         FRAAL2798 RLARQIG----FRLQPDHAALTGLCRPCAAA-------ADPPD---HPR-----------  177
     Francci3_2661 RLATTVG----FTLQPDHAALAGRCRACSVT-------SPSVDGDLAPA-----------  173
         Rxyl_1144 TLLPG-G----HRVEECSLLLSGSCGECLSR-------EGRTR---GPGG----------  137
         FRAAL0074 AIADAEG----FVDVEHTVEIYGTCAGCAAASAAS-------------------------  131
     Francci3_0061 KVANAEG----FVDVVHTVEIYGTCTICAARPAT--------------------------  142
Franean1DRAFT_4289 SVASAEG----FTDVVHTVEIYGTCARCAASRNSDNSPGRPRGGG---------------  192
          Tfu_0856 ELAAQHG----FVDLTHTLELFGTCSDCAAAKPRT-------------------------  129
          MAV_2036 AVAAKHG----FSDVSHTIEIFGTCSECR-------------------------------  135
           MAP2139 AVAAKHG----FSDVSHTIEIFGTCSECR-------------------------------  135
            Mb2380 EVATKHG----FSDVSHTIEIFGTCSDCRS------------------------------  130
          BCG_2373 EVATKHG----FSDVSHTIEIFGTCSDCRS------------------------------  130
            MT2428 EVATKHG----FSDVSHTIEIFGTCSDCRS------------------------------  130
            Rv2359 EVATKHG----FSDVSHTIEIFGTCSDCRS------------------------------  130
            ML0824 EVAAKHG----FSDVSHTIEIFGTCSECRS------------------------------  131
          MUL_3612 NVAARHG----FSDVSHTIEIFGTCSDCRRPGAAALR-----------------------  137
        MSMEG_4487 QVARDHG----FSDVSHTIEIFGVCGDCVERPAGNQT-----------------------  136
         Mvan_3820 EVAHEHG----FSDVSHTIEIFGICSNCA---AGN-------------------------  131
         Mjls_3458 DIAREHG----FSDVSHTIEIFGLCGDCSAR-----------------------------  131
         Mkms_3510 DIAREHG----FSDVSHTIEIFGLCGDCSAR-----------------------------  131
         Mmcs_3447 DIAREHG----FSDVSHTIEIFGLCGDCSAR-----------------------------  131
          nfa14570 AIAGEHG----FTDVSHTIEVFGTCRDCAERRS---------------------------  141
      RHA1_ro01222 SIADAHG----FSDVSHTIEIFGLCKDCSATQ----------------------------  141
           SAV5631 AIAAEHG----YVNVAHTVEIFGTCAECAAAKS---------------------------  138
           SCO2508 AIAAEHG----YVNVAHTVEIFGTCADCAGASGG--------------------------  139
         Noca_1934 AIAAEHG----YAEVSHTLEIFGTCARCG-------------------------------  135
         Acel_2085 RVAARAG----FVDVDHTLEVVGTCPGCAAGRR---------------------------  138
          BAD_0517 GISDKYG----FTIERHTLEVFGLCEDCRKADK---------------------------  132
            BL1128 KVADGHG----FTVESHTLEVFGLCSDCQKEQKAQKAQKAVSE-----------------  146
          Lxx25010 SAAADHG----FSQVQHVVDVFGLCAGCS-AKADLAASSAVP------------------  138
         AAur_2630 RTATEHG----FTEVAHTVEIFGLCPECTAKKAAGKL-----------------------  168
         Arth_2638 RTAAEHG----YTEVAHTVEIFGLCPDCTARKADAG------------------------  154
           PPA0948 DIAAKYE----FSETGHELELFGICSECSAVHTRSVS-----------------------  137
            CE2180 EVARTHG----FSVSSHEAEIFGLCPACRPDTVDGQA-----------------------  175
          NCgl2200 EIATKNG----FALSSHEAEIFGLCADCKEKVT---------------------------  144
           DIP1710 EVAQLHG----FQVTGHDAEIYGLCESCSAATE---------------------------  142
            jk0612 ETAKKFG----YQKSGHTAEIFGLCGDCQKKSAS--------------------------  143
         FRAAL5117 EIARRSG----FAITGYDLQFRGMCPDCQANGSDPARAAGGTGPTVNTHAKGALHAEA--  146
     Francci3_3112 QIACRSG----FAITGYDLRFRGLCPDCQANGSGPARGAAGTGQQ---------------  146
Franean1DRAFT_4643 EIARRSG----FTITGYDLQFRGLCPECQTAGEGHRPGRR--------------------  141
         Rxyl_1228 QVERETG----FVTDSYEITLHGLCRECAAAG----------------------------  138
         Rxyl_1140 GLGRIGG----FSPRAHSLEVYGRCAGCR-------------------------------  152
          outgroup EIERRLG---------RNVKVVNVFMEDRD------------------------------  128
             ruler .......170.......180.......190.......200.......210.......220
```

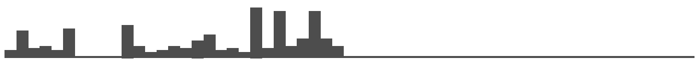

Supplement: Additional File 2 — Multiple alignment of the studied Fur homologues. [file 1471-2148-8-185-S2.pdf]
