# Supplementary figures and images for: On the nature of fur evolution: A phylogenetic approach in Actinobacteria
Source: BMC Evol Biol. 2008 Jun 25;8:185. doi: 10.1186/1471-2148-8-185 (PMC2464607; doi:10.1186/1471-2148-8-185)

Additional File 3

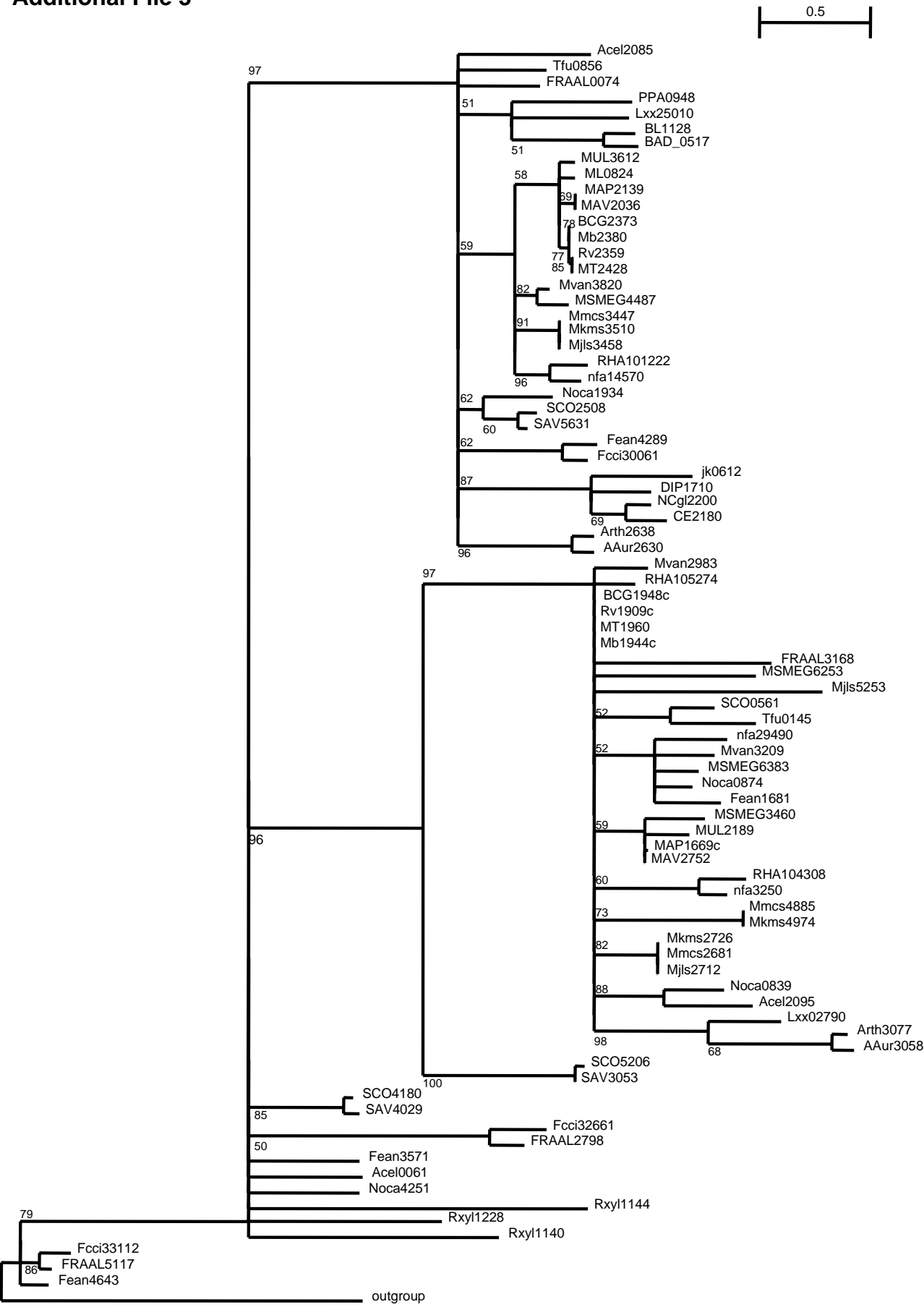

Supplement: Additional File 3 — Maximum-Likelihod tree of the Fur homologues. [file 1471-2148-8-185-S3.pdf]

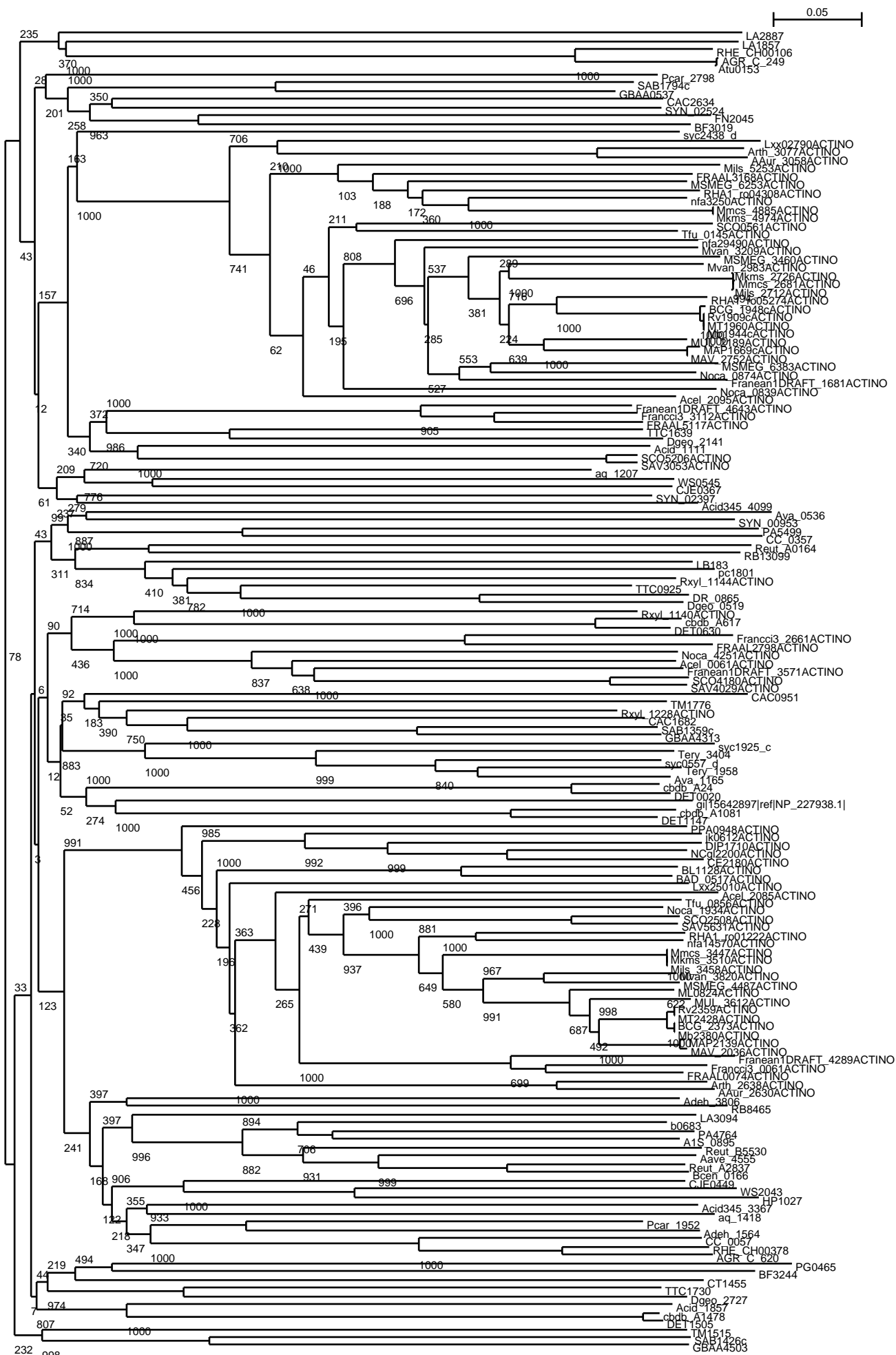

Supplement: Additional File 5 — Neighbour-Joining tree of the Fur homologues (global approach). [file 1471-2148-8-185-S5.pdf]
