# Supplementary material for: On the nature of fur evolution: A phylogenetic approach in Actinobacteria
Source: BMC Evol Biol. 2008 Jun 25;8:185. doi: 10.1186/1471-2148-8-185 (PMC2464607; doi:10.1186/1471-2148-8-185)
Supplement: Additional File 4 — Multiple alignment of the Fur homologues (global approach). [file 1471-2148-8-185-S4.pdf]

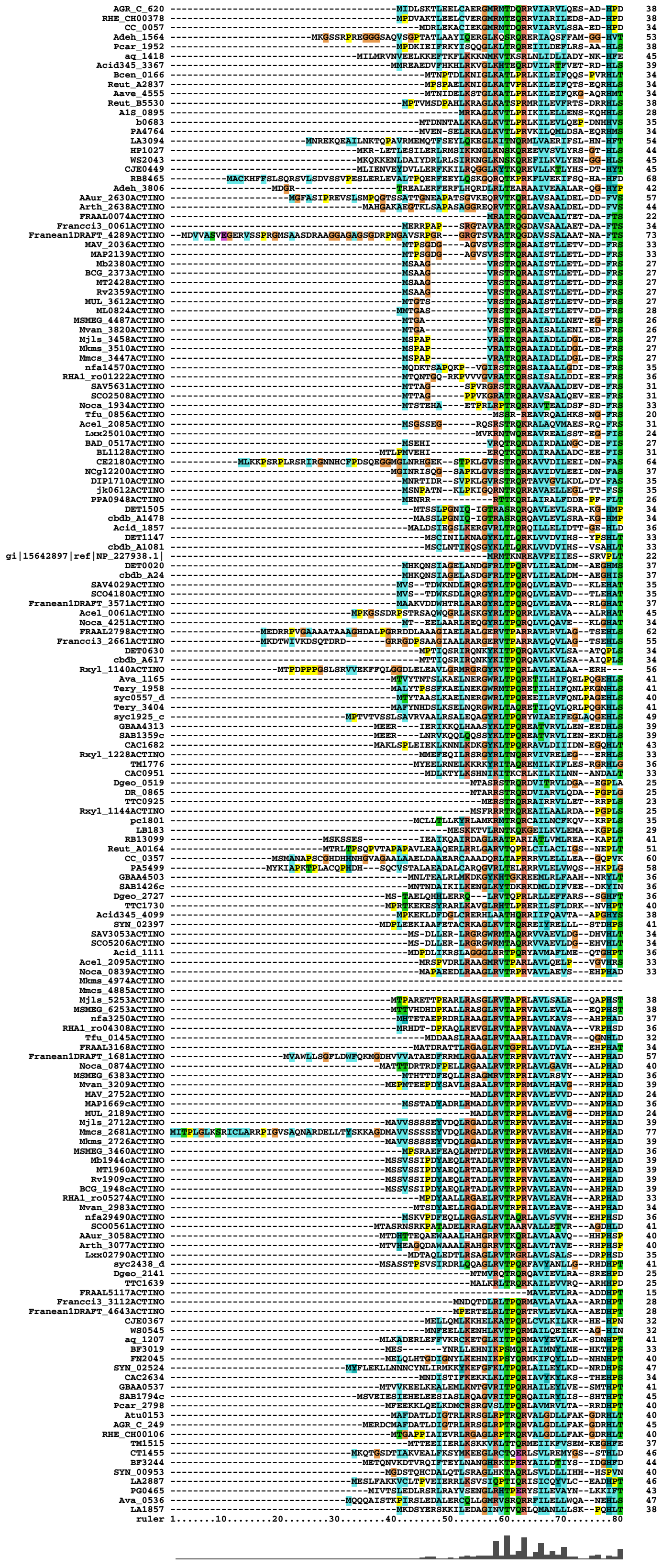

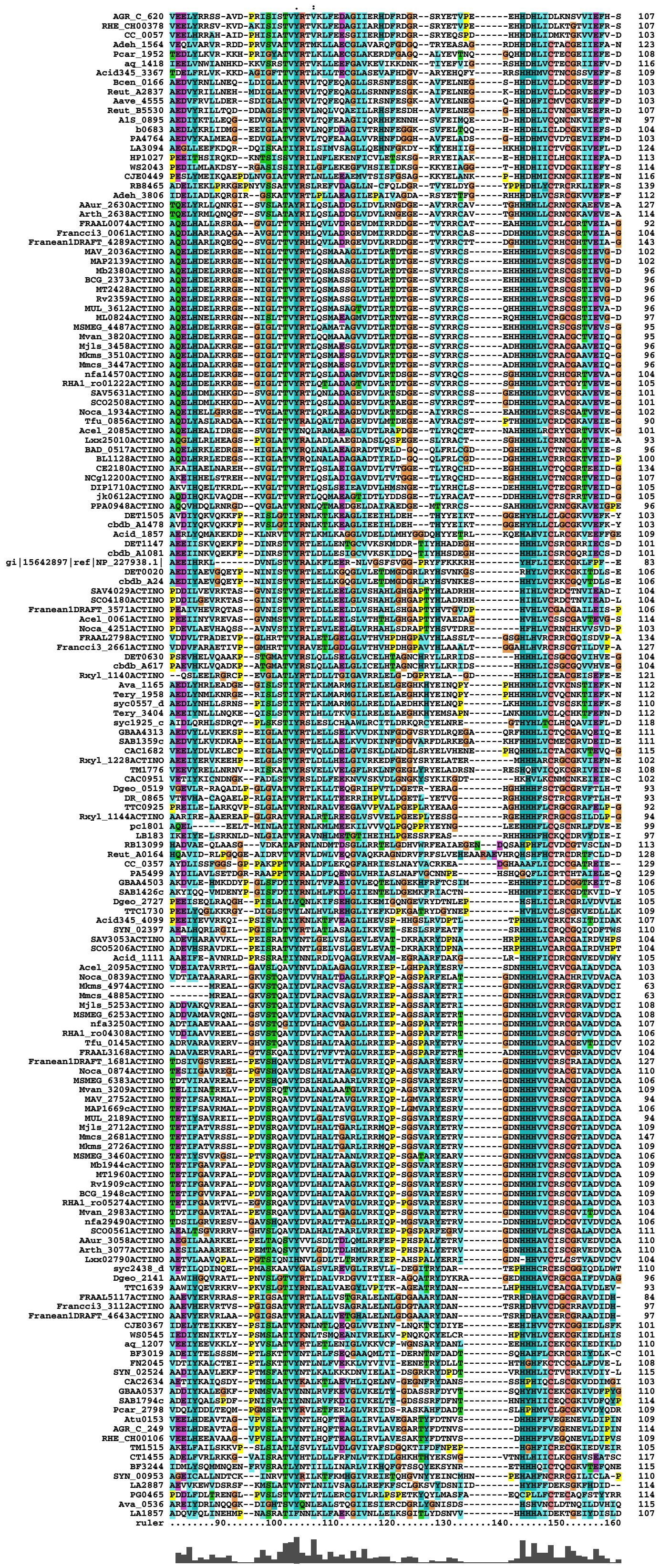

```
AGR_C_620 PEIEALQEKIAR----EHGFKLVDHRLELYGVPLK---------------PEER-------------------------- 142
RHE_CH00378 PEIEALQERIAR----EHGFQLVDHRLELYGIPLK---------------KDDH-------------------------- 142
CC_0057 EEIEALQHAIAK----KLGYKLIDHRLELYGMPLE---------------E----------------------------- 135
Adeh_1564 ERIESLQEMVAR----RHGFEVESHRLELYGRCAR---------------CRPPGRARQEARP----------------- 167
Pcar_1952 ERIEELQSKVAK----EHNFTIRDHRLEIYGRCSK---------------CSSS-------------------------- 143
aq_1418 EEIEKLQEEICK----KYNFKPAHHRLEIFGICED---------------CQKSK------------------------- 152
Acid345_3367 PEIEKVEKDIGK----KHHYRTTRHSFQVFGICEN---------------CSKAEDNGR--------------------- 149
Bcen_0166 AEIESRQQAIAK----ERGFRLQEHSLAMYGSCTT---------------ENCPHRKH---------------------- 142
Reut_A2837 GEIEQRQQSIAR----ERGFALQEHALSLYGNCTK---------------TDCPHRPKR--------------------- 143
Aave_4555 PEIEKRQQLVAK----AKGWVVQDHSMALYGQCAS---------------CAAKASSGNGR------------------- 145
Reut_B5530 ERIEQRQRQVAA----DNDFALREHMLVLYGICLE---------------CRAEQPAG---------------------- 146
A1S_0895 DVIEKEQHSVAE----QHGFTLTGHSLNLYGYCNE---------------PECQEALRKK-------------------- 138
b0683 DSIEARQREIAA----KHGIRLTNHSLYLYGHCAE---------------GDCREDEHAHEGK----------------- 148
PA4764 AEIEKRQKEIVR----ERGFELVDHNLVLY--------------------------VRKKK------------------- 134
LA3094 ERIEQLQEQAAR----ENGFKITGHSLNIYGTCNE---------------HSSSK------------------------- 160
HP1027 PEIENRQNEVVK----KYQAKLISHDMKMFVWCKE---------------CQESEC------------------------ 150
WS2043 KEMEELQEKIAK----GHGAKIISHDMRIFAVCKE---------------CAKAQNL----------------------- 152
CJE0449 PIIERQQALIAK----EHGFKLTGHLMQLYGVCGD---------------CNNQKVKVKI-------------------- 157
RB8465 EELIATRDEAAA----KHGFRVSGHRMLVSGVCRE---------------CGKNRRKKRKQDLV---------------- 184
Adeh_3806 EAFEILQREVAA----RYGFRLEAHHHELVGTCPD---------------CQAREAPRG--------------------- 152
AAur_2630ACTINO PAVETWAARTAT----EHGFTEVAHTVEIFGLCPE---------------CTAKKAAGKL-------------------- 168
Arth_2638ACTINO PAVETWAARTAA----EHGYTEVAHTVEIFGLCPD---------------CTARKADAG--------------------- 154
FRAAL0074ACTINO PEIEAWTAAIAD----AEGFVDVEHTVEIYGTCAG---------------CAAASAAS---------------------- 131
Francci3_0061ACTINO PEIEAWTAKVAN----AEGFVDVVHTVEIYGTCTI---------------CAARPAT----------------------- 142
Franean1DRAFT_4289ACTINO PEIEAWTSSVAS----AEGFTDVVHTVEIYGTCAR---------------CAASRNSDNSPGRPRGGG------------ 192
MAV_2036ACTINO HEVEEWATAVAA----KHGFSDVSHTIEIFGTCSE---------------CR---------------------------- 135
MAP2139ACTINO HEVEEWATAVAA----KHGFSDVSHTIEIFGTCSE---------------CR---------------------------- 135
Mb2380ACTINO HEVEAWAAEVAT----KHGFSDVSHTIEIFGTCSD---------------CRS--------------------------- 130
BCG_2373ACTINO HEVEAWAAEVAT----KHGFSDVSHTIEIFGTCSD---------------CRS--------------------------- 130
MT2428ACTINO HEVEAWAAEVAT----KHGFSDVSHTIEIFGTCSD---------------CRS--------------------------- 130
Rv2359ACTINO HEVEAWAAEVAT----KHGFSDVSHTIEIFGTCSD---------------CRS--------------------------- 130
MUL_3612ACTINO HEVETWAANVAA----RHGFSDVSHTIEIFGTCSD---------------CRRPGAAALR-------------------- 137
ML0824ACTINO HEVEAWAAEVAA----KHGFSDVSHTIEIFGTCSE---------------CRS--------------------------- 131
MSMEG_4487ACTINO GDVETWAAQVAR----DHGFSDVSHTIEIFGVCGD---------------CVERPAGNQT-------------------- 136
Mvan_3820ACTINO GQVETWAAEVAH----EHGFSDVSHTIEIFGICSN---------------CA---AGN---------------------- 131
Mjls_3458ACTINO GQVETWAADIAR----EHGFSDVSHTIEIFGLCGD---------------CSAR-------------------------- 131
Mkms_3510ACTINO GQVETWAADIAR----EHGFSDVSHTIEIFGLCGD---------------CSAR-------------------------- 131
Mmcs_3447ACTINO GQVETWAADIAR----EHGFSDVSHTIEIFGLCGD---------------CSAR-------------------------- 131
nfa14570ACTINO PTVEAWADAIAG----EHGFTDVSHTIEVFGTCRD---------------CAERRS------------------------ 141
RHA1_ro01222ACTINO PTVEQWSQSIAD----AHGFSDVSHTIEIFGLCKD---------------CSATQ------------------------- 141
SAV5631ACTINO PAVEKWAEAIAA----EHGYVNVAHTVEIFGTCAE---------------CAAAKS------------------------ 138
SCO2508ACTINO PAVEKWAEAIAA----EHGYVNVAHTVEIFGTCAD---------------CAGASGG----------------------- 139
Noca_1934ACTINO PAVERWTRAIAA----EHGYAEVSHTLEIFGTCAR---------------CG---------------------------- 135
Tfu_0856ACTINO PAVESWADELAA----QHGFVDLTHTLELFGTCSD---------------CAAAKPRT---------------------- 129
Acel_2085ACTINO ISVERWAERVAA----RAGFVDVDHTLEVVGTCPG---------------CAAGRR------------------------ 138
Lxx25010ACTINO DEVEAWARSAAA----DHGFSQVQHVVDVFGLCAG---------------CSAKADLAASSAVP---------------- 138
BAD_0517ACTINO P-SESWLKGISD----KYGFTIERHTLEVFGLCED---------------CRKADK------------------------ 132
BL1128ACTINO P-SEAWLRKVAD----GHGFTVESHTLEVFGLCSD---------------CQKEQKAQKAQKAVSE-------------- 146
CE2180ACTINO GPVERWAGEVAR----THGFSVSSHEAEIFGLCPA---------------CRPDTVDGQA-------------------- 175
NCgl2200ACTINO GPVETWAQEIAT----KNGFALSSHEAEIFGLCAD---------------CKEKVT------------------------ 144
DIP1710ACTINO GPVEKWAKEVAQ----LHGFQVTGHDAEIYGLCES---------------CSAATE------------------------ 142
jk0612ACTINO GPVEDWAAETAK----KFGYQKSGHTAEIFGLCGD---------------CQKKSAS----------------------- 143
PPA0948ACTINO KILEDWTRDIAA----KYEFSETGHELELFGICSE---------------CSAVHTRSVS-------------------- 137
DET1505 PLEYILSN-VPE----ASGFRVLNTDLRFSGYCDS---------------CS-----------------------KNEDS 140
cbdb_A1478 PLEYILSN-VPE----ASGFRVLNTDLRFSGYCAS---------------CS-----------------------KNEDS 140
Acid_1857 PLQRLRKQ-IES----HFGFQVLLARTEVGGYCAH---------------CQTLRAREVEELRVLTADAAKPPATKSRRA 169
DET1147 DMLSSLREDIQS----KYGFNPNLSHMVINGLCAD---------------CRK--------------------------- 135
cbdb_A1081 DMLAKLRRDIQA----KYSFSPNLSHMVINGICAD---------------CRK--------------------------- 135
gi|15642897|ref|NP_227938.1| ECVDEFLEKLQK----KYDFSEESHLFLIHGICKE---------------CKKEVEK----------------------- 121
DET0020 DMVREQEQSILK----KYGFAADFKNLVIYGQCRK---------------CGDSA------------------------- 142
cbdb_A24 DMVKQLEQSIFK----TYGFAADFKNLVIYGQCQK---------------CSASD------------------------- 142
SAV4029ACTINO GVAAEFTAKLRE----TFGFDTDMKHFAIFGRCQN---------------CALKSSTTKS-------------------- 145
SCO4180ACTINO SVAADFTAKLRE----QFGFDTDMKHFAIFGRCES---------------CSLKGSTTDS-------------------- 145
Franean1DRAFT_3571ACTINO ERLAGIVGELRA----ELGFTVDMDHLTLAGRCAE---------------CTAAGRR----------------------- 144
Acel_0061ACTINO EVVQSLIQRLDD----EHGFAADVHHFAIFGRCST---------------CRSTT------------------------- 150
Noca_4251ACTINO EVIGPLAERLVR----DHGFVIDVGHLAVFGRCTD---------------CEEDR------------------------- 139
FRAAL2798ACTINO DLLDGVADRLAR----QIGFRLQPDHAALTGLCRP---------------CAAAADPPD---HPR--------------- 177
Francci3_2661ACTINO DLLDDVARRLAT----TVGFTLQPDHAALAGRCRA---------------CSVTSPSVDGDLAPA--------------- 173
DET0630 CGLKEITENLSR----QTGFAIDAHLLEFIGRCRN---------------CQKEAGKV---------------------- 143
cbdb_A617 CGLKEITENLSR----QTGFAIDTHLLEFIGRCRN---------------CQKGTGKV---------------------- 143
Rxyl_1140ACTINO CPLDG--AGLGR----IGGFSPRAHSLEVYGRCAG---------------CR---------------------------- 152
Ava_1165 DSILKIGAKTAQ----KEGFHLLDCQMTIHAVCPK---------------CQRALMPL---------------------- 151
Tery_1958 DSILKIGLKQAE----KSGLHLLDCQLTIHTICYEALRKGWPSLVSSDCYCPRALADKENCE------------------ 170
syc0557_d DSVLKIGAKTSE----KEGYHLLDCQLTIHGVCPT---------------CQRSLV------------------------ 147
Tery_3404 DSIMKISQKQAE----KEGVEMLDCQLTIHAICIEALRKGWPALVATNWSCPRTNLEITSKSNNHK-------------- 174
syc1925_c DRVIHLSEGIAD----RYGFQLLNCQLLIMGICAA---------------CRY--------------------------- 152
GBAA4313 DLLGEVERKVEH----DWSFKVKDHRLTFHGICKN---------------CQENETDEK--------------------- 151
SAB1359c DLLPEVENRVEN----EFNFKILDHRLTFHGVCET---------------CQAKGKG----------------------- 149
CAC1682 DLLDELEDCIEN----KYHFLIKNHSVKFYGICSE---------------CRKKL------------------------- 151
Rxyl_1228ACTINO ELMEYLTLQVER----ETGFVTDSYEITLHGLCRE---------------CAAAG------------------------- 138
TM1776 EQVNKIISDISK----KTGYVIKWHDLKFYGICPE---------------CQAKEKEEEKRSK----------------- 152
CAC0951 P-MDQVEEIIKN----KTGFVLIDHELKMKALCKD---------------CMDKSNNEKNS------------------- 143
Dgeo_0519 CP-VALPSGTVY----PGGFIVEAHEVTLYGRCPA---------------CAAAPDSSGAGL------------------ 135
DR_0865 CP-VTLPSSAVY----PGGFVVEAHEVTLYGQCPE---------------CAAR--KTGAH------------------- 132
TTC0925 C---RLEAHLDL----PPGFSAEDHEVVVYGRCPD---------------CA---------------------------- 122
Rxyl_1144ACTINO AL-LRVPAGTLL----PGGHRVEECSLLLSGSCGE---------------CLSREGRTRGPGG----------------- 137
pc1801 S---AVHASSHI----PEGFQLVKCEVTLHGVCGE---------------CNS--------------------------- 130
LB183 CP-IPLDKS-------PKGFTVDTHEIILYGTCSD---------------CNSKAR------------------------ 130
RB13099 VKLTAG---SQR----ESGKVGEVTEILLRGHCND---------------CQ---------------------------- 143
Reut_A0164 VGLPAQPAAPRV----PSGFAIEHVELTVNGVCAD---------------CGARVARHPDSAT----------------- 172
CC_0357 PKASAEIIAAGE----AAGYALTGVTIEAHGLCAD---------------CRS--------------------------- 163
PA5499 PDISRAIVAGAN----SVGFAVESQTVEVVGLCGT---------------CRDQKDA----------------------- 167
GBAA4503 CP---MDFMNKD----FNGYEVTGHKFEIYGRCPK---------------CAK--------------------------- 137
SAB1426c CP---IDQIKLS----LPGVNIHKHKLEVYGVCES---------------CQD--------------------------- 136
Dgeo_2727 -LPQLTPTGPGQ------GWVIAGARVDLQGICPA---------------CQ---------------------------- 135
TTC1730 NLPELEVSPAMQAAAERTGWALEGFRLEFRGKCPD---------------CQR--------------------------- 147
Acid345_4099 FLDPVRLRRAPKGF-----KIEQFAV-DILGTCSKCAK-------------QSAH------------------------- 143
SYN_02397 FLDEVSLPDEIGDWG----RIDSKNV-VVYGICSTCLN-------------QTHKKNF---------------------- 150
SAV3053ACTINO GNPLADLPDSERFG----FTVSDVEV-TYRGVCPNCAAA----------------------------------------- 138
SCO5206ACTINO GNPLADLPDSERFG----FTVSDVEV-TYRGTCPNCAAA----------------------------------------- 138
Acid_1111 EVPRPALGSLGKR------VLRECEL-IFRGLCTRCAPRR-----------ASR-------------------------- 141
Acel_2095ACTINO VGVVPCLSPADAAG----YLVDEAEV-TYWGLCPQCQNHRRP----SADHGGTTAGPTEKHHVPARGEGGDR-------- 166
Noca_0839ACTINO TGEAPCLQASDDAG----YLLDEAEV-TYWGLCANCQ----------------AAGELELL------------------- 143
Mkms_4974ACTINO VGAAPCLDHAPLEDG---FTVDEAEV-VFWGTCADCRA------------EPPAAGASCRL------------------- 108
Mmcs_4885ACTINO VGAAPCLDHAPLEDG---FTVDEAEV-VFWGTCADCRA------------EPPAAGASCRL------------------- 108
Mjls_5253ACTINO VGAAPLLGHPSPRGR---FHRRRGRS-RLLGHMRGLSR------------G--AAGGRGVLPSVTGLA------------ 158
MSMEG_6253ACTINO VGEAPCLEPSDLAG----FAIDEAEV-VYWGVCADCQH------------DS-ATTTRPQSSTI---------------- 154
nfa3250ACTINO VGAAPCLEPSETHG----YAVDEAEV-VFWGLCPDCR-------------AAQD-------------------------- 143
RHA1_ro04308ACTINO VGEAPCLDPSEAHG----FEIDEAEV-VFWGLCSQCRT------------GAQSGTGSAAALS----------------- 152
Tfu_0145ACTINO VGEAPCLAPSQAHG----YTVDEAEV-VFWGVCPSCKA------------KESEH------------------------- 140
FRAAL3168ACTINO TGARPCLEPADDAG----FVIEEAEV-VFWGVCRDCRP------------GDSGSDGVPAR------------------- 148
Franean1DRAFT_1681ACTINO VGSAPCLAASDDRG----FSIDEAEV-FYWGRCPDCSPPA--------TPDRPRAAPDRS-------------------- 174
Noca_0874ACTINO VGGAPCLTASDDHG----FRIDEAEV-VYWGICPACS-------------------TTRS-------------------- 146
MSMEG_6383ACTINO VGEAPCLTGSDHHG----YAIDEAEV-IYWGTCPACAQ----------DPDRAKARTSRSHP------------------ 153
Mvan_3209ACTINO VGEAPCLTASDDLG----FEVDEAEV-IYWGICPACST-----------------APSPQ-------------------- 147
MAV_2752ACTINO VGEAPCLTPSDDDNVLDGFVLDEAEV-IYWGLCADCST------------AGS--------------------------- 134
MAP1669cACTINO VGEAPCLTPSDDDNVLDGFVLDEAEV-IYWGLCAECST------------AGS--------------------------- 146
MUL_2189ACTINO VGEAPCLAPSDDSNVLDGFVVDEAEV-IFWGMCPDCST------------AMPRSQP----------------------- 138
Mjls_2712ACTINO VGEAPCLMPSEHD-ALDGFVLDEAEV-IYWGVCPDCAA------------ESSTADSSRSHP------------------ 157
Mmcs_2681ACTINO VGEAPCLMPSEHD-ALDGFVLDEAEV-IYWGVCPDCAA------------ESSTADSSRSHP------------------ 195
Mkms_2726ACTINO VGEAPCLMPSEHD-ALDGFVLDEAEV-IYWGVCPDCAA------------ESSTADSSRSHP------------------ 157
MSMEG_3460ACTINO VGEAPCLTPSNDDHALDGFLLDEAEV-IYWGRCRECSV------------DGP--------------------------- 146
Mb1944cACTINO VGEAPCLTASDHNG----FLLDEAEV-IYWGLCPDCSI------------SDTS----RSHP------------------ 150
MT1960ACTINO VGEAPCLTASDHNG----FLLDEAEV-IYWGLCPDCSI------------SDTS----RSHP------------------ 150
Rv1909cACTINO VGEAPCLTASDHNG----FLLDEAEV-IYWGLCPDCSI------------SDTS----RSHP------------------ 150
BCG_1948cACTINO VGEAPCLTASDHNG----FLLDEAEV-IYWGLCPDCSI------------SDTS----RSHP------------------ 150
RHA1_ro05274ACTINO VGEAPCLTASDDNG----FLVEEAEV-IYWGLCPDCST------------SQTS----RSNP------------------ 144
Mvan_2983ACTINO VGEAPCLVPSDPDGSLDGFVVDEAEV-VYWGLCPGCLA------------AEIS----RSHQ------------------ 149
nfa29490ACTINO VGEAPCLDASHDHG----FVVDEAEV-IYWGHCPDCSK------------ALSAPPK----------------------- 146
SCO0561ACTINO VGDAPCLTASDDRG----FVIDEAEV-IYRGLCPDCST------------PGSSPAP----------------------- 151
AAur_3058ACTINO VGHAPCLTPHWDENSKP-MTIQIADV-LYQGICQDCQS-------------KQQLPVTSVTQK----------------- 158
Arth_3077ACTINO VGHAPCLTPHWDENSEP-MTIQIADV-MYQGICQDCQR-------------TQKLPAERN-------------------- 155
Lxx02790ACTINO VGHAPCLR--LSEAAG--FAVDTAEV-TFWRLCPSCQE-------------STAATPSRSE------------------- 147
syc2438_d AIAPIDLSAVPAG-----FRPDRYEV-TILGQCAACAT---------------SIRKPKN-------------------- 149
Dgeo_2141 AVPTIPAAALPA-----GFRVTDVRL-EFMGLCPACAAQE----------EEPAASG----------------------- 137
TTC1639 DLPDLRALAREAHP---GVEVREAEV-TFKGLCPACKA---------------ALKG----------------------- 131
FRAAL5117ACTINO PVPDGMMAEIARRS---GFAITGYDL-QFRGMCPDCQANG----------SDPARAAGGTGPTVNTHAKGALHAEA---- 146
Francci3_3112ACTINO PVLDGMMAQIACRS---GFAITGYDL-RFRGLCPDCQANG----------SGPARGAAGTGQQ----------------- 146
Franean1DRAFT_4643ACTINO PVPDGMVAEIARRS---GFTITGYDL-QFRGLCPECQTAG----------EGHRPGRR---------------------- 141
CJE0367 DAKLYEYQEHLEKKIG--NLVNHLSVCAYVDSCKKCH------------------------------------------- 136
WS0545 ---YDELMQNCIKESG--YQIREASL-AMIGLCPECQAK----------------------------------------- 134
aq_1207 -----QKLNIPEEFYG--YKTESYSV-NVYGICPDCRAKMQS-------------------------------------- 144
BF3019 Q--VEMKQVEGLQMDG--HEVSEVHY-YYKGVCKKCLNNIRI----D--------------------------------- 139
FN2045 N--IDYSKSQ--ELLG--CDIEEKHI-YFKGICKNCKGKQN--------------------------------------- 142
SYN_02524 E--FDLKLPG-ADRHG--FEITENHV-DFYGVCPECRKGGDV----RQQKTQLKRKEPPAACEKNP-------------- 171
CAC2634 T--FDNLNKDVSSHTD--YDVISNKL-YFYGICKDCKDKA---------------------------------------- 138
GBAA0537 G--LEKLEEEAAKTTG--FVINSHRL-EIYGVCPECHKA----------------------------------------- 144
SAB1794c Q--LNEIERLAQHMTD--FDVTHHRM-EIYGVCKECQDK----------------------------------------- 148
Pcar_2798 G--FGILEPPGITDDG--FEIIDYAV-TLVGYCRQCRRNVDN-------------------------------------- 146
Atu0153 NLQIDNLPEAPE-------GMEIAHVDVVIRLRRKRG------------------------------------------- 139
AGR_C_249 NLQIDNLPEAPE-------GMEIAHVDVVIRLRRKRG------------------------------------------- 144
RHE_CH00106 NLTIDNLPEPPE-------GMEIAHVDVVIRLRAKQG------------------------------------------- 139
TM1515 ELKLGEVKGKRE-----------KIELVIKGICEE---------------CLKKVKDTM--------------------- 138
CT1455 ELPELMETLCVG-----HGYSLGSFSLQLFGECADKEA------------CARRISNKHKEKT----------------- 163
BF3244 ALQNAIENTKLS------KFQLSHYSLYIYGICSKCDR------------ANKRKRVNNNNKKEK--------------- 162
SYN_00953 QTLSLFREGLPG----YEHLIIDQITVNISGLCDD---------------CRRHVNDPVS-------------------- 151
LA2887 PSLLSLSSKLPP-----EFLVNKTDILLTGNLVSET-------------------------------------------- 145
PG0465 NVKSILADKDLR----PPRFSYRQAIICLYGICDKCRKKQ----------SALKKAADKAAPKKKK-------------- 166
Ava_0536 LPEAFIQEVEQR-----TGVKITDYSINFYGYRHP----------------QEEE------------------------- 149
LA1857 SKLQEKVLSELK----QDFKLKTGSSLENCNLSIT---------------LKGKKNP----------------------- 145
ruler .......170.......180.......190.......200.......210.......220.......230.......240
```

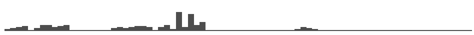

```
                 AGR_C_620 -----   142
               RHE_CH00378 -----   142
                   CC_0057 -----   135
                 Adeh_1564 -----   167
                 Pcar_1952 -----   143
                   aq_1418 -----   152
              Acid345_3367 -----   149
                 Bcen_0166 -----   142
                Reut_A2837 -----   143
                 Aave_4555 -----   145
                Reut_B5530 -----   146
                  A1S_0895 -----   138
                     b0683 -----   148
                    PA4764 -----   134
                    LA3094 -----   160
                    HP1027 -----   150
                    WS2043 -----   152
                   CJE0449 -----   157
                    RB8465 -----   184
                 Adeh_3806 -----   152
           AAur_2630ACTINO -----   168
           Arth_2638ACTINO -----   154
           FRAAL0074ACTINO -----   131
       Francci3_0061ACTINO -----   142
  Franean1DRAFT_4289ACTINO -----   192
            MAV_2036ACTINO -----   135
             MAP2139ACTINO -----   135
              Mb2380ACTINO -----   130
            BCG_2373ACTINO -----   130
              MT2428ACTINO -----   130
              Rv2359ACTINO -----   130
            MUL_3612ACTINO -----   137
              ML0824ACTINO -----   131
          MSMEG_4487ACTINO -----   136
           Mvan_3820ACTINO -----   131
           Mjls_3458ACTINO -----   131
           Mkms_3510ACTINO -----   131
           Mmcs_3447ACTINO -----   131
            nfa14570ACTINO -----   141
        RHA1_ro01222ACTINO -----   141
             SAV5631ACTINO -----   138
             SCO2508ACTINO -----   139
           Noca_1934ACTINO -----   135
            Tfu_0856ACTINO -----   129
           Acel_2085ACTINO -----   138
            Lxx25010ACTINO -----   138
            BAD_0517ACTINO -----   132
              BL1128ACTINO -----   146
              CE2180ACTINO -----   175
            NCgl2200ACTINO -----   144
             DIP1710ACTINO -----   142
              jk0612ACTINO -----   143
             PPA0948ACTINO -----   137
                   DET1505 R----   141
                cbdb_A1478 R----   141
                 Acid_1857 RTRRS   174
                   DET1147 -----   135
                cbdb_A1081 -----   135
gi|15642897|ref|NP_227938.1| -----   121
                   DET0020 -----   142
                  cbdb_A24 -----   142
             SAV4029ACTINO -----   145
             SCO4180ACTINO -----   145
  Franean1DRAFT_3571ACTINO -----   144
           Acel_0061ACTINO -----   150
           Noca_4251ACTINO -----   139
           FRAAL2798ACTINO -----   177
       Francci3_2661ACTINO -----   173
                   DET0630 -----   143
                 cbdb_A617 -----   143
           Rxyl_1140ACTINO -----   152
                  Ava_1165 -----   151
                 Tery_1958 -----   170
                 syc0557_d -----   147
                 Tery_3404 -----   174
                 syc1925_c -----   152
                  GBAA4313 -----   151
                  SAB1359c -----   149
                   CAC1682 -----   151
           Rxyl_1228ACTINO -----   138
                    TM1776 -----   152
                   CAC0951 -----   143
                 Dgeo_0519 -----   135
                   DR_0865 -----   132
                   TTC0925 -----   122
           Rxyl_1144ACTINO -----   137
                    pc1801 -----   130
                     LB183 -----   130
                   RB13099 -----   143
                Reut_A0164 -----   172
                   CC_0357 -----   163
                    PA5499 -----   167
                  GBAA4503 -----   137
                  SAB1426c -----   136
                 Dgeo_2727 -----   135
                   TTC1730 -----   147
              Acid345_4099 -----   143
                 SYN_02397 -----   150
             SAV3053ACTINO -----   138
             SCO5206ACTINO -----   138
                 Acid_1111 -----   141
           Acel_2095ACTINO -----   166
           Noca_0839ACTINO -----   143
           Mkms_4974ACTINO -----   108
           Mmcs_4885ACTINO -----   108
           Mjls_5253ACTINO -----   158
          MSMEG_6253ACTINO -----   154
             nfa3250ACTINO -----   143
        RHA1_ro04308ACTINO -----   152
            Tfu_0145ACTINO -----   140
           FRAAL3168ACTINO -----   148
  Franean1DRAFT_1681ACTINO -----   174
           Noca_0874ACTINO -----   146
          MSMEG_6383ACTINO -----   153
           Mvan_3209ACTINO -----   147
            MAV_2752ACTINO -----   134
            MAP1669cACTINO -----   146
            MUL_2189ACTINO -----   138
           Mjls_2712ACTINO -----   157
           Mmcs_2681ACTINO -----   195
           Mkms_2726ACTINO -----   157
          MSMEG_3460ACTINO -----   146
             Mb1944cACTINO -----   150
              MT1960ACTINO -----   150
             Rv1909cACTINO -----   150
           BCG_1948cACTINO -----   150
        RHA1_ro05274ACTINO -----   144
           Mvan_2983ACTINO -----   149
            nfa29490ACTINO -----   146
             SCO0561ACTINO -----   151
           AAur_3058ACTINO -----   158
           Arth_3077ACTINO -----   155
            Lxx02790ACTINO -----   147
                 syc2438_d -----   149
                 Dgeo_2141 -----   137
                   TTC1639 -----   131
           FRAAL5117ACTINO -----   146
       Francci3_3112ACTINO -----   146
  Franean1DRAFT_4643ACTINO -----   141
                   CJE0367 -----   136
                    WS0545 -----   134
                   aq_1207 -----   144
                    BF3019 -----   139
                    FN2045 -----   142
                 SYN_02524 -----   171
                   CAC2634 -----   138
                  GBAA0537 -----   144
                  SAB1794c -----   148
                 Pcar_2798 -----   146
                   Atu0153 -----   139
                 AGR_C_249 -----   144
               RHE_CH00106 -----   139
                    TM1515 -----   138
                    CT1455 -----   163
                    BF3244 -----   162
                 SYN_00953 -----   151
                    LA2887 -----   145
                    PG0465 -----   166
                  Ava_0536 -----   149
                    LA1857 -----   145
                     ruler .....
```
