# Supplementary material for: On the nature of fur evolution: A phylogenetic approach in Actinobacteria
Source: BMC Evol Biol. 2008 Jun 25;8:185. doi: 10.1186/1471-2148-8-185 (PMC2464607; doi:10.1186/1471-2148-8-185)

```
                                                       ******************* *  ************ **
       Badolescentis --TTTTTGTGGAGGGTTCGATTCTGGCTCAGGATGAACGCTGGCGGCGTGCTTAACACATGCAAGTCGAACGGGATCGGC   78
             Blongum --TTTTTGTGGAGGGTTCGATTCTGGCTCAGGATGAACGCTGGCGGCGTGCTTAACACATGCAAGTCGAACGGGATCCAT   78
          Aaurescens ---------------------------TCAGGATGAACGCTGGCGGCGTGCTTAACACATGCAAGTCGAACGATGATCCC   53
               AFB24 -----------AGAGTTTGATCCTGGCTCAGGATGAACGCTGGCGGCGTGCTTAACACATGCAAGTCGAACGATGATCCC   69
              TTwist --TTATTATGGAGAGTTTGATCCTGGCTCAGGACGAACGCTGGCGGCGTGCTTAACACATGCAAGTCGAACGGAAATCAG   78
            TTW08/27 --TTATTATGGAGAGTTTGATCCTGGCTCAGGACGAACGCTGGCGGCGTGCTTAACACATGCAAGTCGAACGGAAATCAG   78
               Lxyli ---------------------------------CGAACGCTGGCGGCGTGCTTAACACATGCAAGTCGAACGATGAACCA   47
        Savermitilis ----TTCACGGAGAGTTTGATCCTGGCTCAGGACGAACGCTGGCGGCGTGCTTAACACATGCAAGTCGAACGATGA----   72
         Scoelicolor --CATTCACGGAGAGTTTGATCCTGGCTCAGGACGAACGCTGGCGGCGTGCTTAACACATGCAAGTCGAACGATGA----   74
         FalniACN14a ----TTGATGGAGAGTTTGATCCTGGCTCAGGACGAACGCTGGCGGCGTGCTTAACACATGCAAGTCGAGCGAGGGG---   73
               FCcI3 ----TTGATGGAGAGTTTGATCCTGGCTCAGGACGAACGCTGGCGGCGTGCTTAACACATGCAAGTCGAGCGGGGAG---   73
            FEAN1pec ----TTGATGGAGAGTTTGATCCTGGCTCAGGACGAACGCTGGCGGCGTGCTTAACACATGCAAGTCGAGCGAGGAG---   73
     Acellulolyticus --------------GTTTGATCCTGGCTCAGGACGAACGCTGGCGGCGTGCTTAACACATGCAAGTCGAGCGGAAAGGCC   66
     MbovisAF2122/97 -TTTTGTTTGGAGAGTTTGATCCTGGCTCAGGACGAACGCTGGCGGCGTGCTTAACACATGCAAGTCGAACGGAAAGGTC   79
  MtuberculosisH37Rv -TTTTGTTTGGAGAGTTTGATCCTGGCTCAGGACGAACGCTGGCGGCGTGCTTAACACATGCAAGTCGAACGGAAAGGTC   79
       MbovisPasteur -TTTTGTTTGGAGAGTTTGATCCTGGCTCAGGACGAACGCTGGCGGCGTGCTTAACACATGCAAGTCGAACGGAAAGGTC   79
MtuberculosisCDC1551 --TTTGTTTGGAGAGTTTGATCCTGGCTCAGGACGAACGCTGGCGGCGTGCTTAACACATGCAAGTCGAACGGAAAGGTC   78
           Mulcerans -TTTTGTTTGGAGAGTTTGATCCTGGCTCAGGACGAACGCTGGCGGCGTGCTTAACACATGCAAGTCGAACGGAAAGGTC   79
              Mavium -------------AGTTTGATCCTGGCTCAGGACGAACGCTGGCGGCGTGCTTAACACATGCAAGTCGAACGGAAAGGCC   67
          MaviumK-10 -TTTTGTTTGGAGAGTTTGATCCTGGCTCAGGACGAACGCTGGCGGCGTGCTTAACACATGCAAGTCGAACGGAAAGGCC   79
          MlepraeTN --TTTGTTTGGAGAGTTTGATCCTGGCTCAGGACGAACGCTGGCGGCGTGCTTAACACATGCAAGTCGAACGGAAAGGTC   78
                MJLS -----------AGAGTTTGATTCTGGCTCAGGACGAACGCTGGCGGCGTGCTTAACACATGCAAGTCGAACGGAAAGGCC   69
                MKMS -----------AGAGTTTGATTCTGGCTCAGGACGAACGCTGGCGGCGTGCTTAACACATGCAAGTCGAACGGAAAGGCC   69
                MMCS ----------GAGAGTTTGATTCTGGCTCAGGACGAACGCTGGCGGCGTGCTTAACACATGCAAGTCGAACGGAAAGGCC   70
          Msmegmatis TTTTTGTTTGGAGAGTTTGATCCTGGCTCAGGACGAACGCTGGCGGCGTGCTTAACACATGCAAGTCGAACGGAAAGGCC   80
        Mvanbaalenii -----------AGAGTTTGATTCTGGCTCAGGACGAACGCTGGCGGCGTGCTTAACACATGCAAGTCGAACGGAAAGGCC   69
          Nfarcinica -TCTTCAACGGAGAGTTTGATCCTGGCTCAGGACGAACGCTGGCGGCGTGCTTAACACATGCAAGTCGAGCGGTAAGGCC   79
               RRHA1 --CTTCAACGGAGAGTTTGATCCTGGCTCAGGACGAACGCTGGCGGCGTGCTTAACACATGCAAGTCGAGCGGTAAGGCC   78
        Cdiphtheriae --------------------------------ACGAACGCTGGCGGCGTGCTTAACACATGCAAGTCGAACGGAAAGGC-   47
           Cjeikeium ------TATGGAGAGTTTGATCCTGGCTCAGGACGAACGCTGGCGGCGTGCTTAACACATGCAAGTCGAACGGAAAGGCT   74
          Cefficiens ----------------------CTGGCTCAGGACGAACGCTGGCGGCGTGCTTAACACATGCAAGTCGAACGATGAAGC-   57
         Cglutamicum ------TGTGGAGAGTTTGATCCTGGCTCAGGACGAACGCTGGCGGCGTGCTTAACACATGCAAGTCGAACGCTGAAAC-   73
        Nocardioides -----------AGAGTTTGATCCTGGCTCAGGACGAACGCTGGCGGCGTGCTTAACACATGCAAGTCGAGCGGAAAGGCC   69
              Pacnes -----------AGAGTTTGATCCTGGCTCAGGACGAACGCTGGCGGCGTGCTTAACACATGCAAGTCGAACGGAAAGGCC   69
            TfuscaYX --------------------------------ACGAACGCTGGCGGCGTGCTTAACACATGCAAGTCGAGCGGTAAGGCC   48
       Rxylanophilus -------------------------------GACGAACGCTGGCGGCGTGCTTTAGGCATGCAAGTCGAACGCGAAAGCC   49
               ruler 1.......10........20........30........40........50........60........70........80
```

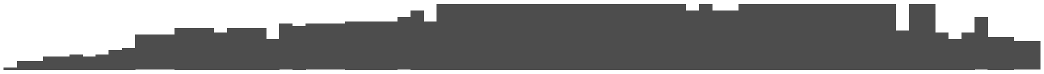

```
                                                 ** *****************  ****    * ** **     *    ** *
       Badolescentis TGGA------GCTTGCTCCGGCCGTGAGAGTGGCGAACGGGTGAGTAATGCGTGACCGACCTGCCCCATACACC-GGAAT   151
             Blongum CAGG------CTTTGCTT-GGTGGTGAGAGTGGCGAACGGGTGAGTAATGCGTGACCGACCTGCCCCATACACC-GGAAT   150
          Aaurescens T-GG--------CTTGCTGG-GGGGATTAGTGGCGAACGGGTGAGTAACACGTGAGTAACCTGCCCTTGACTCT-GGGAT   122
               AFB24 --AG--------CTTGCTGG-GGG-ATTAGTGGCGAACGGGTGAGTAACACGTGAGTAACCTGCCCTTAACTCT-GGGAT   136
              TTwist GGAG--------CTTGCTCCCTGTGATTAGTGGCGAACGGGTGAGTAACACGTGAGCAACCTGCCCCTAACTTT-GGGAT   149
            TTW08/27 GGAG--------CTTGCTCCCTGTGATTAGTGGCGAACGGGTGAGTAACACGTGAGCAACCTGCCCCTAACTTT-GGGAT   149
               Lxyli GGAG--------CTTGCTCTTGGGAATTAGTGGCGAACGGGTGAGTAACACGTGAGTAACCTGCCCTTGACTCT-GGGAT   118
        Savermitilis --AG--------CCCTTCGGGGTGGATTAGTGGCGAACGGGTGAGTAACACGTGGGCAATCTGCCCTGCACTCT-GGGAC   141
         Scoelicolor --AC--------CACTTCGGTGGGGATTAGTGGCGAACGGGTGAGTAACACGTGGGCAATCTGCCCTTCACTCT-GGGAC   143
         FalniACN14a CTTC--------------GGC---TCCTAGCGGCGAACGGGTGAGTAACACGTGGGCAACCTGCCCCGAGCTCT-GGAAT   135
               FCcI3 CTTC--------------GGC---TCTCAGCGGCGAACGGGTGAGTAACACGTGGGCAACCTGCCCCGAGCTCT-GGAAT   135
            FEAN1pec CTT----------------GC---TCCTAGCGGCGAACGGGTGAGTAACACGTGGGCAACCTGCCCCAAGCTCT-GGAAT   133
     Acellulolyticus CTTC--------------GGGGTACTCGAGCGGCGAACGGGTGAGTAACACGTGGGCAACCTACCCCCAGCACC-GGGAT   131
     MbovisAF2122/97 TCT------------TCGGAGATACTCGAGTGGCGAACGGGTGAGTAACACGTGGGTGATCTGCCCTGCACTTC-GGGAT   146
  MtuberculosisH37Rv TCT------------TCGGAGATACTCGAGTGGCGAACGGGTGAGTAACACGTGGGTGATCTGCCCTGCACTTC-GGGAT   146
       MbovisPasteur TCT------------TCGGAGATACTCGAGTGGCGAACGGGTGAGTAACACGTGGGTGATCTGCCCTGCACTTC-GGGAT   146
MtuberculosisCDC1551 TCT------------TCGGAGATACTCGAGTGGCGAACGGGTGAGTAACACGTGGGTGATCTGCCCTGCACTTC-GGGAT   145
           Mulcerans TCT------------TCGGAGATACTCGAGTGGCGAACGGGTGAGTAACACGTGGGCGATCTGCCCTGCACTTC-GGGAT   146
              Mavium TCT------------TCGGAGGTACTCGAGTGGCGAACGGGTGAGTAACACGTGGGCAATCTGCCCTGCACTTC-GGGAT   134
          MaviumK-10 TCT------------TCGGAGGTACTCGAGTGGCGAACGGGTGAGTAACACGTGGGCAATCTGCCCTGCACTTC-GGGAT   146
           MlepraeTN TCTAAAAAATCTTTTTTAGAGATACTCGAGTGGCGAACGGGTGAGTAACACGTGGGTAATCTGCCCTGCACTTCAGGGAT   158
                MJLS CTT------------C--GGGGTACTCGAGTGGCGAACGGGTGAGTAACACGTGGGTGATCTGCCCTGCACTTT-GGGAT   134
                MKMS CTT------------C--GGGGTACTCGAGTGGCGAACGGGTGAGTAACACGTGGGTGATCTGCCCTGCACTTT-GGGAT   134
                MMCS CTT------------C--GGGGTACTCGAGTGGCGAACGGGTGAGTAACACGTGGGTGATCTGCCCTGCACTTT-GGGAT   135
          Msmegmatis CTT------------TCGGGGGTACTCGAGTGGCGAACGGGTGAGTAACACGTGGGTGATCTGCCCTGCACTTT-GGGAT   147
        Mvanbaalenii CTT------------C--GGGGTACTCGAGTGGCGAACGGGTGAGTAACACGTGGGTGATCTGCCCTGCACTTT-GGGAT   134
          Nfarcinica CTT--------------CGGGGTACACGAGCGGCGAACGGGTGAGTAACACGTGGGTGATCTGCCCTGTACTTC-GGGAT   144
               RRHA1 CTT--------------CGGGGTACACGAGCGGCGAACGGGTGAGTAACACGTGGGTGATCTGCCCTGCACTTC-GGGAT   143
        Cdiphtheriae -CTA------GCTTGCTAGG--TACTCGAGTGGCGAACGGGTGAGTAACACGTGGGTGATCTGCCTCGTACTTC-GGGAT   117
           Cjeikeium CCTT------GCTTGCAAGGGGTACTCGAGTGGCGAACGGGTGAGTAACACGTGGGTGATCTGCCTTGCACTTC-GGGAT   147
          Cefficiens CCT-------GCTTGCAG-GG-TGGATTAGTGGCGAACGGGTGAGTAACACGTGGGTGATCTGCCCCGCACTTC-GGGAT   127
         Cglutamicum CAGA------GCTTGCTTTGG-TGGATGAGTGGCGAACGGGTGAGTAACACGTGGGTGATCTGCCCTACACTTT-GGGAT   145
        Nocardioides CT------------TTCGGGGGTACTCGAGCGGCGAACGGGTGAGTAACACGTGAGTAATCTGCCCTTCACTTT-GGGAT   136
              Pacnes CTGC--------TTTTGTGGGGTGCTCGAGTGGCGAACGGGTGAGTAACACGTGAGTAACCTGCCCTTGACTTT-GGGAT   140
            TfuscaYX CCTT------------CGGGGGTACACGAGCGGCGAACGGGTGAGTAACACGTGAGTAACCTGCCCCTGACTCT-GGGAT   115
       Rxylanophilus CCTT-----------CGGGGGTGAGTAGAGTGGCGAACGGGTGAGTAACACGTGGGTAACCTGCCCCTCGCAGG-GGGAT   117
               ruler ........90.......100.......110.......120.......130.......140.......150.......160
```

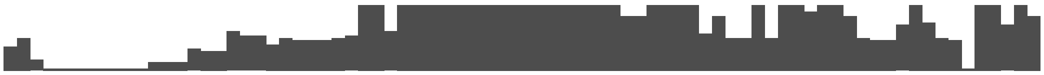

```
                    *      ******      **** *    *                                **  *
Badolescentis        AGCTCCTGGAAACGGGTGGTAATGCCGGATGCT--CCAGTTGGATGCATGTCCTTCTG--GGAAAGA-TTCTAT------   220
Blongum              AGCTCCTGGAAACGGGTGGTAATGCCGGATGCT--CCAGTTGATCGCATGGTCTTCTG--GGAAAGC-TTTCG-------   218
Aaurescens           AAGCCTGGGAAACTGGGTCTAATACCGGATA-CGACCA-TCTGGCGCATGTCATGGTGGTGGAAAGC-TTTT-G------   192
AFB24                AAGCCTGGGAAACTGGGTCTAATACCGGATA-TGACTC-CTCATCGCATGGTGGGG-GGTGGAAAGC-TTTTTG------   206
TTwist               AACTTCGGGAAACCGGGGCTAATACCAAATA-CGACCC-ATGAGGGCATCCTCTGTGGGTGGAAAGA-ATTT--------   218
TTW08/27             AACTTCGGGAAACCGGGGCTAATACCAAATA-CGACCC-ATGAGGGCATCCTCTGTGGGTGGAAAGA-ATTT--------   218
Lxyli                AACCTCCGGAAACGGAAGCTAATACCGGATA-CGAACC-GCGGAGGCATCTCCAGCGGTTGGAAAGA-ACTT--------   187
Savermitilis         AAGCCCTGGAAACGGGGTCTAATACCGGATAAT-ACTC-TCGCAGGCATC-TGTGAGGGTTAAAAGC-TCCG-G------   210
Scoelicolor          AAGCCCTGGAAACGGGGTCTAATACCGGATACTGACCC-TCGCAGGCATC-TGCGAGGGTCGAAAGC-TCCG-G------   213
FalniACN14a          AACCTCGGGAAACCGGGGCTAATGCCGGATATG-ACGC-TACCGGGCATC-TGGTGGTGTGGAAAGA--TTTAT------   204
FCcI3                AACCTCGGGAAACCGGGGCTAATGCCGGATATG-ACAT-TGCCGGGCATC-TGGTGGTGTGGAAAGA--TTTAT------   204
FEAN1pec             AACTCCGGGAAACCGGGGCTAATGCCGGATATG-ACAT-CATCGGGCATC-TGGTGATGTGGAAAGA--TTTAT------   202
Acellulolyticus      AACCCCGGGAAACCGGGGCTAATACCGGATATGCACCT-CGGGTGGCATC-ACCTGAGGTGGAAAGC--TTTTG------   201
MbovisAF2122/97      AAGCCTGGGAAACTGGGTCTAATACCGGATAGG-ACCA-CGGGATGCATG-TCTTGTGGTGGAAAGCGCTTTAG------   217
MtuberculosisH37Rv   AAGCCTGGGAAACTGGGTCTAATACCGGATAGG-ACCA-CGGGATGCATG-TCTTGTGGTGGAAAGCGCTTTAG------   217
MbovisPasteur        AAGCCTGGGAAACTGGGTCTAATACCGGATAGG-ACCA-CGGGATGCATG-TCTTGTGGTGGAAAGCGCTTTAG------   217
MtuberculosisCDC1551 AAGCCTGGGAAACTGGGTCTAATACCGGATAGG-ACCA-CGGGATGCATG-TCTTGTGGTGGAAAGCGCTTTAG------   216
Mulcerans            AAGCCTGGGAAACTGGGTCTAATACCGGATAGG-ACCA-CGGGATTCATG-TCCTGTGGTGGAAAGC--TTTTG------   215
Mavium               AAGCCTGGGAAACTGGGTCTAATACCGGATAGG-ACCT-CAAGACGCATG-TCTTCTGGTGGAAAGC--TTTTG------   203
MaviumK-10           AAGCCTGGGAAACTGGGTCTAATACCGGATAGG-ACCT-CAAGACGCATG-TCTTCTGGTGGAAAGC--TTTTG------   215
MlepraeTN            AAGCTTGGGAAACTGGGTCTAATACCGGATAGG-ACTT-CAAGGCGCATG-TCTTGTGGTGGAAAGC-TTTTTG------   228
MJLS                 AAGCCTGGGAAACTGGGTCTAATACCGAATACA-CCCTGCTGGTCGCATGGCCTGGTGGGGAAAGC--TTTTG------   205
MKMS                 AAGCCTGGGAAACTGGGTCTAATACCGAATACA-CCCTGCTGGTCGCATGGCCTGGTGGGGAAAGC--TTTTG------   205
MMCS                 AAGCCTGGGAAACTGGGTCTAATACCGAATACA-CCCTGCTGGTCGCATGGCCTGGTGGGGAAAGC--TTTTG------   206
Msmegmatis           AAGCCTGGGAAACTGGGTCTAATACCGAATACA-CCCTGCTGGTCGCATGGCCTGGTAGGGGAAAGC--TTTTG------   218
Mvanbaalenii         AAGCCTGGGAAACTGGGTCTAATACCGAATACA-CCCTTCTGGCTGCATGGTCTGGTTGGGGAAAGC--TTTTG------   205
Nfarcinica           AAGCCTGGGAAACTGGGTCTAATACCGGATATG-ACCT-TACATCGCATG-GTGTTTGGTGGAAAGA--TTTAT------   213
RRHA1                AAGCCTGGGAAACTGGGTCTAATACCGGATATG-ACCT-TCGGCTGCATG-GCTGAGGGTGGAAAGG--TTTAC------   212
Cdiphtheriae         AAGCCTGGGAAACTGGGTCTAATACTGGATAGG-ACCATGCTTTAGTGTG----TGTGGTGGAAAG--TTTTTT------   184
Cjeikeium            AAGCCTGGGAAACTGGGTCTAATACCGGATAGG-ACCATGCTTTAGTGTG----TGTGGTGGAAAG--TTTTTT------   214
Cefficiens           AAGCCTGGGAAACTGGGTCTAATACCGGATACG-ACCCCCTGGTAGGTCA----GGGGGTGGAAAGA-TTTTAT------   195
Cglutamicum          AAGCCTGGGAAACTGGGTCTAATACCGAATATTCACACCACCGTAGGG-G----TGGTGTGGAAAGC-TTTATG------   213
Nocardioides         AACTACCGGAAACGGTGGCTAATACCGGATACG-ACAC-AGGTACGCATGTGCTCTGTGTGGAAAGT--TTTTT------   206
Pacnes               AACTTCAGGAAACTGGGGCTAATACCGGATAGG-AGCT-CCTGCTGCATG-GTGGGGTTGGAAAGT--TTCGG------   209
TfuscaYX             AAGCCTGGGAAACTGGGTCTAATACCGGATATG-ACCT-CGTGCCGCATGGTGTGGGGTGGAAAGGTGTTTTTC------   187
Rxylanophilus        AACCGGGGGAAACCCCGGCTAATACCCCGTACG-CTTGCCGGGGCGCATGCTCCGGCAAGGAAAGGTAGCTTCGGCCATC   196
ruler                .......170.......180.......190.......200.......210.......220.......230.......240
```

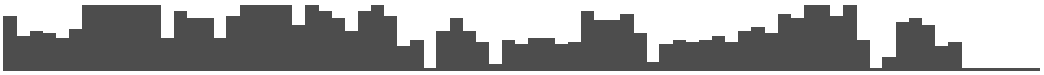

```
                      **     ** *     *** *  ** ** * *  ** * ***   ***  ***  * *   ** ******* * *****
Badolescentis        CGGTATGGGATGGGGTCGCGTCCTATCAGCTTGATGGCGGGGTAACGGCCCACCATGGCTTCGACGGGTAGCCGGCCTGA   300
Blongum              CGGTATGGGATGGGGTCGCGTCCTATCAGCTTGACGGCGGGGTAACGGCCCACCGTGGCTTCGACGGGTAGCCGGCCTGA   298
Aaurescens           TGGTTTTGGATGGACTCGCGGCCTATCAGCTTGTTGGTGGGGTAATGGCCTACCAAGGCGACGACGGGTAGCCGGCCTGA   272
AFB24                TGGTTTTGGATGGACTCGCGGCCTATCAGCTTGTTGGTGAGGTAATGGCTCACCAAGGCGACGACGGGTAGCCGGCCTGA   286
TTwist               CGGTTGGGGATGGGCTCGCGGCCTATCAGCTTGTTGGTGGGGTAACGGCCTACCAAGGCATCGACGGGTAGCCGGCCTGA   298
TTW08/27             CGGTTGGGGATGGGCTCGCGGCCTATCAGCTTGTTGGTGGGGTAACGGCCTACCAAGGCATCGACGGGTAGCCGGCCTGA   298
Lxyli                CGGTCGAGGATGGACTCGCGGCCTATCAGGTAGTTGGTGAGGTAACGGCTCACCAAGCCTACGACGGGTAGCCGGCCTGA   267
Savermitilis         CGGTGCAGGATGAGCCCGCGGCCTATCAGCTTGTTGGTGAGGTAGTGGCTCACCAAGGCGACGACGGGTAGCCGGCCTGA   290
Scoelicolor          CGGTGAAGGATGAGCCCGCGGCCTATCAGCTTGTTGGTGAGGTAATGGCTCACCAAGGCGACGACGGGTAGCCGGCCTGA   293
FalniACN14a          CGGCTCGGGATGGGCCCGCGGCCTATCAGCTTGTTGGTGGGGTGATGGCCTACCAAGGCGACGACGGGTAGCCGGCCTGA   284
FCcI3                CGGCTCGGGATGGGCCCGCGGCCTATCAGCTTGTTGGTGGGGTGATGGCCTACCAAGGCGACGACGGGTAGCCGGCCTGA   284
FEAN1pec             CGGCTTGGGATGGGCCCGCGGCCTATCAGCTTGTTGGTGGGGTGATGGCCTACCAAGGCGACGACGGGTAGCCGGCCTGA   282
Acellulolyticus      CGGCTGGGGATGGGCCCGCGGCCTATCAGCTTGTTGGTGGGGTAACGGCCTACCAAGGCGACGACGGGTAGCCGGCCTGA   281
MbovisAF2122/97      CGGTGTGGGATGAGCCCGCGGCCTATCAGCTTGTTGGTGGGGTGACGGCCTACCAAGGCGACGACGGGTAGCCGGCCTGA   297
MtuberculosisH37Rv   CGGTGTGGGATGAGCCCGCGGCCTATCAGCTTGTTGGTGGGGTGACGGCCTACCAAGGCGACGACGGGTAGCCGGCCTGA   297
MbovisPasteur        CGGTGTGGGATGAGCCCGCGGCCTATCAGCTTGTTGGTGGGGTGACGGCCTACCAAGGCGACGACGGGTAGCCGGCCTGA   297
MtuberculosisCDC1551 CGGTGTGGGATGAGCCCGCGGCCTATCAGCTTGTTGGTGGGGTGACGGCCTACCAAGGCGACGACGGGTAGCCGGCCTGA   296
Mulcerans            CGGTGTGGGATGGGCCCGCGGCCTATCAGCTTGTTGGTGGGGTAACGGCCTACCAAGGCGACGACGGGTAGCCGGCCTGA   295
Mavium               CGGTGTGGGATGGGCCCGCGGCCTATCAGCTTGTTGGTGGGGTGACGGCCTACCAAGGCGACGACGGGTAGCCGGCCTGA   283
MaviumK-10           CGGTGTGGGATGGGCCCGCGGCCTATCAGCTTGTTGGTGGGGTGACGGCCTACCAAGGCGACGACGGGTAGCCGGCCTGA   295
MlepraeTN            CGGTGCAGGATGGGCCCGCGGCCTATCAGCTTGTTGGTGGGGTGACGGCCTACCAAGGCGACGACGGGTAGCCGGCCTGA   308
MJLS                 CGGTGTGGGATGGGCCCGCGGCCTATCAGCTTGTTGGTGAGGTTACGGCTCACCAAGGCGACGACGGGTAGCCGGCCTGA   285
MKMS                 CGGTGTGGGATGGGCCCGCGGCCTATCAGCTTGTTGGTGAGGTTACGGCTCACCAAGGCGACGACGGGTAGCCGGCCTGA   285
MMCS                 CGGTGTGGGATGGGCCCGCGGCCTATCAGCTTGTTGGTGAGGTTACGGCTCACCAAGGCGACGACGGGTAGCCGGCCTGA   286
Msmegmatis           CGGTGTGGGATGGGCCCGCGGCCTATCAGCTTGTTGGTGGGGTGATGGCCTACCAAGGCGACGACGGGTAGCCGGCCTGA   298
Mvanbaalenii         CGGTGTGGGATGGGCCCGCGGCCTATCAGCTTGTTGGTGAGGTTACGGCTCACCAAGGCGACGACGGGTAGCCGGCCTGA   285
Nfarcinica           CGGTACAGGATGGGCCCGCGGCCTATCAGCTTGTTGGTGGGGTAATGGCCTACCAAGGCGACGACGGGTAGCCGGCCTGA   293
RRHA1                TGGTGCAGGATGGGCCCGCGGCCTATCAGCTTGTTGGTGGGGTAATGGCCTACCAAGGCGACGACGGGTAGCCGACCTGA   292
Cdiphtheriae         CGGTACGAGATGAGCCCGCGGCCTATCAGCTTGTTGGTGGGGTAATGGCCTACCAAGGCGTCGACGGGTAGCCGGCCTGA   264
Cjeikeium            CGGTGCAAGATGAGCCCGCGGCCTATCAGCTTGTTGGTGGGGTAATGGCCTACCAAGGCGACGACGGGTAGCCGGCCTGA   294
Cefficiens           CGGTGTGGGATGAGCCTGCGGCCTATCAGCTTGTTGGTGGGGTAATGGCCTACCAAGGCGTCGACGGGTAGCCGGCCTGA   275
Cglutamicum          CGGTGTGGGATGAGCCTGCGGCCTATCAGCTTGTTGGTGGGGTAATGGCCTACCAAGGCGTCGACGGGTAGCCGGCCTGA   293
Nocardioides         CGGTGGGGGATGTGCTCGCGGCCTATCAGCTTGTTGGTGGGGTAATGGCCTACCAAGGCTTCGACGGGTAGCCGGCCTGA   286
Pacnes               CGGTTGGGGATGGACTCGCGGCTTATCAGCTTGTTGGTGGGGTAGTGGCTTACCAAGGCTTTGACGGGTAGCCGGCCTGA   289
TfuscaYX             CGGTCAGGGATGGGCTCGCGGCCTATCAGCTTGTTGGTGGGGTAACGGCCTACCAAGGCGATGACGGGTAGCCGGCCTGA   267
Rxylanophilus        CGGCGAGGGAGGGGCCCGCGGCCCATTAGCTAGTTGGTGGGGTAACGGCCCACCAAGGCGACGATGGGTAGCTGGCCTGA   276
ruler                .......250.......260.......270.......280.......290.......300.......310.......320
```

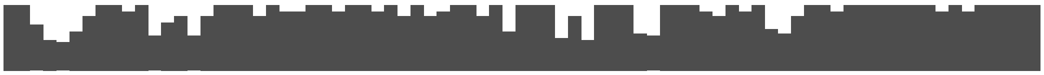

```
                     ***** *  * ****** *********** *************** *************   ***** ***  *******
       Badolescentis GAGGGCGACCGGCCACATTGGGACTGAGATACGGCCCAGACTCCTACGGGAGGCAGCAGTGGGGAATATTGCACAATGGG   380
             Blongum GAGGGCGACCGGCCACATTGGGACTGAGATACGGCCCAGACTCCTACGGGAGGCAGCAGTGGGGAATATTGCACAATGGG   378
          Aaurescens GAGGGTGACCGGCCACACTGGGACTGAGACACGGCCCAGACTCCTACGGGAGGCAGCAGTGGGGAATATTGCACAATGGG   352
               AFB24 GAGGGTGACCGGCCACACTGGGACTGAGACACGGCCCAGACTCCTACGGGAGGCAGCAGTGGGGAATATTGCACAATGGG   366
              TTwist GAGGGTGATCGGCCACACTGGGACTGAGACACGGCCCAGACTCCTACGGGAGGCAGCAGTGGGGAATATTGCACAATGGG   378
            TTW08/27 GAGGGTGATCGGCCACACTGGGACTGAGACACGGCCCAGACTCCTACGGGAGGCAGCAGTGGGGAATATTGCACAATGGG   378
               Lxyli GAGGGTGACCGGCCACACTGGGACTGAGACACGGCCCAGACTCCTACGGGAGGCAGCAGTGGGGAATATTGCACAATGGG   347
         Savermitilis GAGGGCGACCGGCCACACTGGGACTGAGACACGGCCCAGACTCCTACGGGAGGCAGCAGTGGGGAATATTGCACAATGGG   370
         Scoelicolor GAGGGCGACCGGCCACACTGGGACTGAGACACGGCCCAGACTCCTACGGGAGGCAGCAGTGGGGAATATTGCACAATGGG   373
         FalniACN14a GAGGGCGACCGGCCACACTGGGACTGAGACACGGCCCAGACTCCTACGGGAGGCAGCAGTGGGGAATATTGCGCAATGGG   364
               FCcI3 GAGGGCGACCGGCCACACTGGGACTGAGACACGGCCCAGACTCCTACGGGAGGCAGCAGTGGGGAATATTGCGCAATGGG   364
            FEAN1pec GAGGGCGATCGGCCACACTGGGACTGAGACACGGCCCAGACTCCTACGGGAGGCAGCAGTGGGGAATATTGCGCAATGGG   362
      Acellulolyticus GAGGGCGACCGGCCACACTGGGACTGAGACACGGCCCAGACTCCTACGGGAGGCAGCAGTGGGGAATATTGCGCAATGGG   361
      MbovisAF2122/97 GAGGGTGTCCGGCCACACTGGGACTGAGATACGGCCCAGACTCCTACGGGAGGCAGCAGTGGGGAATATTGCACAATGGG   377
   MtuberculosisH37Rv GAGGGTGTCCGGCCACACTGGGACTGAGATACGGCCCAGACTCCTACGGGAGGCAGCAGTGGGGAATATTGCACAATGGG   377
        MbovisPasteur GAGGGTGTCCGGCCACACTGGGACTGAGATACGGCCCAGACTCCTACGGGAGGCAGCAGTGGGGAATATTGCACAATGGG   377
MtuberculosisCDC1551 GAGGGTGTCCGGCCACACTGGGACTGAGATACGGCCCAGACTCCTACGGGAGGCAGCAGTGGGGAATATTGCACAATGGG   376
           Mulcerans GAGGGTGTCCGGCCACACTGGGACTGAGATACGGCCCAGACTCCTACGGGAGGCAGCAGTGGGGAATATTGCACAATGGG   375
              Mavium GAGGGTGTCCGGCCACACTGGGACTGAGATACGGCCCAGACTCCTACGGGAGGCAGCAGTGGGGAATATTGCACAATGGG   363
          MaviumK-10 GAGGGTGTCCGGCCACACTGGGACTGAGATACGGCCCAGACTCCTACGGGAGGCAGCAGTGGGGAATATTGCACAATGGG   375
           MlepraeTN GAGGGTGTACGGCCACACTGGGACTGAGATACGGCCCAGACTCCTACGGGAGGCAGCAGTGGGGAATATTGCACAATGGG   388
                MJLS GAGGGTGACCGGCCACACTGGGACTGAGATACGGCCCAGACTCCTACGGGAGGCAGCAGTGGGGAATATTGCACAATGGG   365
                MKMS GAGGGTGACCGGCCACACTGGGACTGAGATACGGCCCAGACTCCTACGGGAGGCAGCAGTGGGGAATATTGCACAATGGG   365
                MMCS GAGGGTGACCGGCCACACTGGGACTGAGATACGGCCCAGACTCCTACGGGAGGCAGCAGTGGGGAATATTGCACAATGGG   366
          Msmegmatis GAGGGTGACCGGCCACACTGGGACTGAGATACGGCCCAGACTCCTACGGGAGGCAGCAGTGGGGAATATTGCACAATGGG   378
         Mvanbaalenii GAGGGTGACCGGCCACACTGGGACTGAGATACGGCCCAGACTCCTACGGGAGGCAGCAGTGGGGAATATTGCACAATGGG   365
          Nfarcinica GAGGGCGACCGGCCACACTGGGACTGAGACACGGCCCAGACTCCTACGGGAGGCAGCAGTGGGGAATATTGCACAATGGG   373
               RRHA1 GAGGGTGACCGGCCACACTGGGACTGAGACACGGCCCAGACTCCTACGGGAGGCAGCAGTGGGGAATATTGCACAATGGG   372
         Cdiphtheriae GAGGGTGGACGGCCACATTGGGACTGAGATACGGCCCAGACTCCTACGGGAGGCAGCAGTGGGGAATATTGCACAATGGG   344
            Cjeikeium GAGGGTGTACGGCCACATTGGGACTGAGACACGGCCCAGACTCCTACGGGAGGCAGCAGTGGGGAATATTGCACAATGGG   374
          Cefficiens GAGGGTGATCGGCCACATTGGGACTGAGACACGGCCCAGACTCCTACGGGAGGCAGCAGTGGGGAATATTGCACAATGGG   355
         Cglutamicum GAGGGTGTACGGCCACATTGGGACTGAGACACGGCCCAGACTCCTACGGGAGGCAGCAGTGGGGAATATTGCACAATGGG   373
         Nocardioides GAGGGTGACCGGCCACACTGGGACTGAGACACGGCCCAGACTCCTACGGGAGGCAGCAGTGGGGAATATTGGACAATGGG   366
              Pacnes GAGGGTGACCGGCCACATTGGGACTGAGATACGGCCCAGACTCCTACGGGAGGCAGCAGTGGGGAATATTGCACAATGGG   369
            TfuscaYX GAGGGCGACCGGCCACACTGGGACTGAGACACGGCCCAGACTCCTGCGGGAGGCAGCAGTGGGGAATATTGCGCAATGGG   347
        Rxylanophilus GAGGGTGGTCAGCCACACTGGGACTGAGACACGGCCCAGACTCCTACGGGAGGCAGCAGCCAGGAATCTTGCGCAATGGG   356
               ruler .......330.......340.......350.......360.......370.......380.......390.......400
```

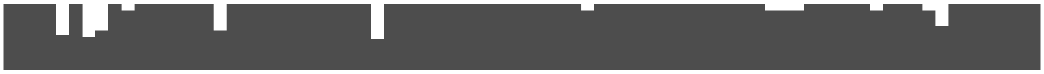

```
                     ** ********  **** ********** * ** *  *** ******* *****   ** *        *  ***
       Badolescentis CGCAAGCCTGATGCAGCGACGCCGCGTGCGGGATGACGGCCTTCGGGTTGTAAACCGCTTTTGACTGGGAGCAAG--CCC   458
             Blongum CGCAAGCCTGATGCAGCGACGCCGCGTGAGGGATGGAGGCCTTCGGGTTGTAAACCTCTTTTATCGGGGAGCAAG--CG-   455
          Aaurescens CGCAAGCCTGATGCAGCGACGCCGCGTGAGGGATGACGGCCTTCGGGTTGTAAACCTCTTTCAGTAGGGAAGAAG--CGA   430
               AFB24 CGAAAGCCTGATGCAGCGACGCCGCGTGAGGGATGACGGCCTTCGGGTTGTAAACCTCTTTCAGTAGGGAAGAAG--CGA   444
              TTwist CGCAAGCCTGATGCAGCAACGCCGCGTGGGGGAAGAAGGCCTTCGGGTTGTAAACCTCTTTTGGCAGGGAAGAAG--CGA   456
            TTW08/27 CGCAAGCCTGATGCAGCAACGCCGCGTGGGGGAAGAAGGCCTTCGGGTTGTAAACCTCTTTTGGCAGGGAAGAAG--CGA   456
               Lxyli CGAAAGCCTGATGCAGCAACGCCGCGTGAGGGATGACGGCCTTCGGGTTGTAAACCTCTTTTAGTAGGGAAGAAG--CGA   425
         Savermitilis CGAAAGCCTGATGCAGCGACGCCGCGTGAGGGATGACGGCCTTCGGGTTGTAAACCTCTTTCAGCAGGGAAGAAG--CGA   448
         Scoelicolor CGAAAGCCTGATGCAGCGACGCCGCGTGAGGGATGACGGCCTTCGGGTTGTAAACCTCTTTCAGCAGGGAAGAAG--CGA   451
         FalniACN14a CGAAAGCCTGACGCAGCGACGCCGCGTGGGGGATGACGGCCTTCGGGTTGTAAACCTCTTTCAGCAGGGACGAAG--CGC   442
               FCcI3 CGGAAGCCTGACGCAGCGACGCCGCGTGGGGGATGACGGCCTTCGGGTTGTAAACCTCTTTCAGCAGGGACGAAG--CGA   442
            FEAN1pec CGAAAGCCTGACGCAGCGACGCCGCGTGAGGGATGACGGCCTTCGGGTTGTAAACCTCTTTCAGCAGGGACGAAG--CGA   440
      Acellulolyticus CGAAAGCCTGACGCAGCGACGCCGCGTGGGGGAAGAAGGCCTTCGGGTTGTAAACCCCTTTCAGCAGGAACGAAG--CGA   439
      MbovisAF2122/97 CGCAAGCCTGATGCAGCGACGCCGCGTGGGGGATGACGGCCTTCGGGTTGTAAACCTCTTTCACCATCGACGAAG--GTC   455
   MtuberculosisH37Rv CGCAAGCCTGATGCAGCGACGCCGCGTGGGGGATGACGGCCTTCGGGTTGTAAACCTCTTTCACCATCGACGAAG--GTC   455
        MbovisPasteur CGCAAGCCTGATGCAGCGACGCCGCGTGGGGGATGACGGCCTTCGGGTTGTAAACCTCTTTCACCATCGACGAAG--GTC   455
MtuberculosisCDC1551 CGCAAGCCTGATGCAGCGACGCCGCGTGGGGGATGACGGCCTTCGGGTTGTAAACCTCTTTCACCATCGACGAAG--GTC   454
           Mulcerans CGCAAGCCTGATGCAGCGACGCCGCGTGGGGGATGACGGCCTTCGGGTTGTAAACCTCTTTCACCATCGACGAAG--GTT   453
              Mavium CGCAAGCCTGATGCAGCGACGCCGCGTGGGGGATGACGGCCTTCGGGTTGTAAACCTCTTTCACCATCGACGAAG--GTC   441
          MaviumK-10 CGCAAGCCTGATGCAGCGACGCCGCGTGGGGGATGACGGCCTTCGGGTTGTAAACCTCTTTCACCATCGACGAAG--GTC   453
           MlepraeTN CGCAAGCCTGATGCAGCGACGCCGCGTGGGGGATGACGGCTTTCGGGTTGTAAACCTCTTTCACCATCGACGAAG--GTC   466
                MJLS CGCAAGCCTGATGCAGCGACGCCGCGTGGGGGATGACGGCCTTCGGGTTGTAAACCTCTTTCAGTAGGGACGAAG--CGC   443
                MKMS CGCAAGCCTGATGCAGCGACGCCGCGTGGGGGATGACGGCCTTCGGGTTGTAAACCTCTTTCAGTAGGGACGAAG--CGC   443
                MMCS CGCAAGCCTGATGCAGCGACGCCGCGTGGGGGATGACGGCCTTCGGGTTGTAAACCTCTTTCAGTAGGGACGAAG--CGC   444
          Msmegmatis CGCAAGCCTGATGCAGCGACGCCGCGTGAGGGATGACGGCCTTCGGGTTGTAAACCTCTTTCAGCACAGACGAAG--CGC   456
         Mvanbaalenii CGCAAGCCTGATGCAGCGACGCCGCGTGAGGGATGACGGCCTTCGGGTTGTAAACCTCTTTCGCCAGGGACGAAG--CGC   443
          Nfarcinica CGAAAGCCTGATGCAGCGACGCCGCGTGAGGGATGACGGCCTTCGGGTTGTAAACCTCTTTCGACAGGGACGAAG--CGC   451
               RRHA1 CGAAAGCCTGATGCAGCGACGCCGCGTGAGGGATGACGGCCTTCGGGTTGTAAACCTCTTTCAGCAGGGACGAAG--CGA   450
         Cdiphtheriae CGCAAGCCTGATGCAGCGACGCCGCGTGGGGGATGACGGCCTTCGGGTTGTAAACCTCTTTCGCTAGGGACGAAG--CTT   422
            Cjeikeium CGCAAGCCTGATGCAGCGACGCCGCGTGGGGGATGACGGCCTTCGGGTTGTAAACTCCTTTCGCTAGGGAAGAAG--CCA   452
          Cefficiens CGCAAGCCTGATGCAGCGACGCCGCGTGGGGGATGACGGCCTTCGGGTTGTAAACTCCTTTCGACAGGGACGAAG--CTT   433
         Cglutamicum CGCAAGCCTGATGCAGCGACGCCGCGTGGGGGATGAAGGCCTTCGGGTTGTAAACTCCTTTCGCTAGGGACGAAG--CCT   451
         Nocardioides CGGAAGCCTGATCCAGCAACGCCGCGTGAGGGATGACGGCCTTCGGGTTGTAAACCTCTTTCAGTACCGACGAAG--CAC   444
              Pacnes CGGAAGCCTGATGCAGCAACGCCGCGTGCGGGATGACGGCCTTCGGGTTGTAAACCGCTTTCGCCTGTGACGAAG--CGT   447
            TfuscaYX CGGAAGCCTGACGCAGCGACGCCGCGTGGGGGATGAAGGCCTTCGGGTTGTAAACCTCTTTTACCACTCACGAAGGCCCC   427
        Rxylanophilus CGAAAGCCTGACGCAGCAACGCCGCGTGGGCGATGAAGGCCTTCGGGTCGTAAAGCCCTGTTGTCGGGGACGAAGGGCGA   436
               ruler .......410.......420.......430.......440.......450.......460.......470.......480
```

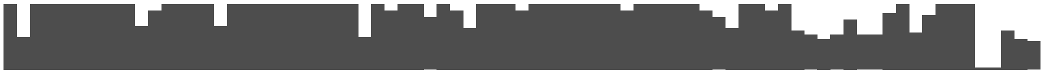

```
                                                       **       *  ***  ***** *********************** *******
      Badolescentis TTC-------------GGGGTGAGTGTACCTTTCGAATAAGCACCGGCTAACTACGTGCCAGCAGCCGCGGTAATACGTA  525
            Blongum ----------------AGAGTGAGTTTACCCGTTGAATAAGCACCGGCTAACTACGTGCCAGCAGCCGCGGTAATACGTA  519
        Aaurescens AAG-----------------TGACGGTACCTGCAGAAGAAGCGCCGGCTAACTACGTGCCAGCAGCCGCGGTAATACGTA  493
              AFB24 AAG-----------------TGACGGTACCTGCAGAAGAAGCGCCGGCTAACTACGTGCCAGCAGCCGCGGTAATACGTA  507
             TTwist AAG-----------------TGACGGTACCTGCAGAAAAAGCGCCGGCTAACTACGTGCCAGCAGCCGCGGTAATACGTA  519
           TTW08/27 AAG-----------------TGACGGTACCTGCAGAAAAAGCGCCGGCTAACTACGTGCCAGCAGCCGCGGTAATACGTA  519
              Lxyli AAG-----------------TGACGGTACCTGCAGAAAAAGCACCGGCTAACTACGTGCCAGCAGCCGCGGTAATACGTA  488
       Savermitilis AAG-----------------TGACGGTACCTGCAGAAGAAGCGCCGGCTAACTACGTGCCAGCAGCCGCGGTAATACGTA  511
        Scoelicolor AAG-----------------TGACGGTACCTGCAGAAGAAGCGCCGGCTAACTACGTGCCAGCAGCCGCGGTAATACGTA  514
       FalniACN14a AAG-----------------TGACGGTACCTGCAGAAGAAGCACCGGCCAACTACGTGCCAGCAGCCGCGGTAATACGTA  505
              FCcI3 GAG-----------------TGACGGTACCTGCAGAAGAAGCACCGGCCAACTACGTGCCAGCAGCCGCGGTAATACGTA  505
           FEAN1pec GAG-----------------TGACGGTACCTGCAGAAGAAGCACCGGCCAACTACGTGCCAGCAGCCGCGGTAATACGTA  503
    Acellulolyticus AAG-----------------TGACGGTACCTGCAGAAGAAGCACCGGCTAACTACGTGCCAGCAGCCGCGGTAATACGTA  502
     MbovisAF2122/97 CGGGTTCTCTC-----GGATTGACGGTAGGTGGAGAAGAAGCACCGGCCAACTACGTGCCAGCAGCCGCGGTAATACGTA  530
 MtuberculosisH37Rv CGGGTTCTCTC-----GGATTGACGGTAGGTGGAGAAGAAGCACCGGCCAACTACGTGCCAGCAGCCGCGGTAATACGTA  530
       MbovisPasteur CGGGTTCTCTC-----GGATTGACGGTAGGTGGAGAAGAAGCACCGGCCAACTACGTGCCAGCAGCCGCGGTAATACGTA  530
MtuberculosisCDC1551 CGGGTTCTCTC-----GGATTGACGGTAGGTGGAGAAGAAGCACCGGCCAACTACGTGCCAGCAGCCGCGGTAATACGTA  529
          Mulcerans CGGGTTTTCTC-----GGATTGACGGTAGGTGGAGAAGAAGCACCGGCCAACTACGTGCCAGCAGCCGCGGTAATACGTA  528
             Mavium CGGGTTTTCTC-----GGATTGACGGTAGGTGGAGAAGAAGCACCGGCCAACTACGTGCCAGCAGCCGCGGTAATACGTA  516
          MaviumK-10 CGGGTTTTCTC-----GGATTGACGGTAGGTGGAGAAGAAGCACCGGCCAACTACGTGCCAGCAGCCGCGGTAATACGTA  528
          MlepraeTN TGGGTTTTCTC-----GGATTGACGGTAGGTGGAGAAGAAGCACCGGCCAACTACGTGCCAGCAGCCGCGGTAATACGTA  541
               MJLS AAG-----------------TGACGGTACCTACAGAAGAAGGACCGGCCAACTACGTGCCAGCAGCCGCGGTAATACGTA  506
               MKMS AAG-----------------TGACGGTACCTACAGAAGAAGGACCGGCCAACTACGTGCCAGCAGCCGCGGTAATACGTA  506
               MMCS AAG-----------------TGACGGTACCTACAGAAGAAGGACCGGCCAACTACGTGCCAGCAGCCGCGGTAATACGTA  507
         Msmegmatis AAG-----------------TGACGGTATGTGCAGAAGAAGGACCGGCCAACTACGTGCCAGCAGCCGCGGTAATACGTA  519
       Mvanbaalenii AAG-----------------TGACGGTACCTGGAGAAGAAGGACCGGCCAACTACGTGCCAGCAGCCGCGGTAATACGTA  506
         Nfarcinica AAG-----------------TGACGGTACCTGTAGAAGAAGCACCGGCCAACTACGTGCCAGCAGCCGCGGTAATACGTA  514
              RRHA1 AAG-----------------TGACGGTACCTGCAGAAGAAGCACCGGCTAACTACGTGCCAGCAGCCGCGGTAATACGTA  513
       Cdiphtheriae TTGTG-----------------ACGGTACCTAGATAAGAAGCACCGGCTAACTACGTGCCAGCAGCCGCGGTAATACGTA  485
          Cjeikeium CTGTGTGG------------TGACGGTACCTGGATAAGAAGCACCGGCTAACTACGTGCCAGCAGCCGCGGTAATACGTA  520
         Cefficiens TT--GT---------------GACGGTACCTGTAGAAGAAGCACCGGCTAACTACGTGCCAGCAGCCGCGGTAATACGTA  496
        Cglutamicum TATGGT---------------GACGGTACCTGGAGAAGAAGCACCGGCTAACTACGTGCCAGCAGCCGCGGTAATACGTA  516
       Nocardioides CGGTGATGGT--------GGTGACGGTAGGTACAGAAGAAGCACCGGCCAACTACGTGCCAGCAGCCGCGGTAATACGTA  516
             Pacnes GAGTGACGGT--------AATG--GGTA-------AAGAAGCACCGGCTAACTACGTGCCAGCAGCCGCGGTGATACGTA  510
           TfuscaYX TGGTTTTCTGG-----GGGTTGACGGTAGGTGGTGAATAAGGACCGGCTAACTACGTGCCAGCAGCCGCGGTAATACGTA  502
      Rxylanophilus AGGGTTAATAGCCCCTAGCCTGACGGTACCCGACGAGGAAGCCCCGGCTAACTACGTGCCAGCAGCCGCGGTAATACGTA  516
              ruler .......490.......500.......510.......520.......530.......540.......550.......560
```

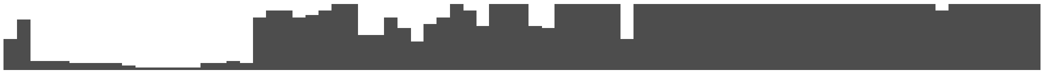

```
                    **   * ****** ****** * * *********** *   ***** **        **     *** **       ***
      Badolescentis GGGTGCAAGCGTTATCCGGAATTATTGGGCGTAAAGGGCTCGTAGGCGGTTCGTCGCGTCCGGTGTGAAAGTCCATCGCT  605
            Blongum GGGTGCAAGCGTTATCCGGAATTATTGGGCGTAAAGGGCTCGTAGGCGGTTCGTCGCGTCCGGTGTGAAAGTCCATCGCT  599
         Aaurescens GGGCGCAAGCGTTATCCGGAATTATTGGGCGTAAAGAGCTCGTAGGCGGTTTGTCGCGTCTGCTGTGAAAGACCGGGGCT  573
              AFB24 GGGCGCAAGCGTTATCCGGAATTATTGGGCGTAAAGAGCTCGTAGGCGGTTTGTCGCGTCTGCCGTGAAAGTCCGGGGCT  587
             TTwist GGGCGCGAGCGTTGTCCGGAATTATTGGGCGTAAAGAGCTCGTAGGCGGTTTGTCACGTCTGCCGTGAAAATCCGAGGCT  599
           TTW08/27 GGGCGCGAGCGTTGTCCGGAATTATTGGGCGTAAAGAGCTCGTAGGCGGTTTGTCACGTCTGCCGTGAAAATCCGAGGCT  599
              Lxyli GGGTGCGAGCGTTGTCCGGAATTATTGGGCGTAAAGAGCTCGTAGGCGGTCTGTCGCGTCTGCTGTGAAAACCCGAGGCT  568
       Savermitilis GGGCGCAAGCGTTGTCCGGAATTATTGGGCGTAAAGAGCTCGTAGGCGGCTTGTCACGTCGGGTGTGAAAGCCCGGGGCT  591
        Scoelicolor GGGCGCAAGCGTTGTCCGGAATTATTGGGCGTAAAGAGCTCGTAGGCGGCTTGTCACGTCGGTTGTGAAAGCCCGGGGCT  594
        FalniACN14a GGGTGCAAGCGTTGTCCGGAATTATTGGGCGTAAAGAGCTCGTAGGCGGCCTGTCGCGTCGGCTGTGAAAACCCGGGGCT  585
              FCcI3 GGATGCAAGCGTTGTCCGGAATTATTGGGCGTAAAGAGCTCGTAGGCGGCCTGTTGCGTCGGCTGTGAAAACCCGGGGCT  585
           FEAN1pec GGGTGCAAGCGTTGTCCGGAATTATTGGGCGTAAAGAGCTCGTAGGCGGCCTGTCGCGTCGGCTGTGAAAACCCGGGGCT  583
    Acellulolyticus GGGTGCAAGCGTTGTCCGGATTTACTGGGCGTAAAGAGCTCGTAGGCGGTCTGTCGCGTCGGATGTGAAAACCCAGGGCT  582
    MbovisAF2122/97 GGGTGCGAGCGTTGTCCGGAATTACTGGGCGTAAAGAGCTCGTAGGTGGTTTGTCGCGTTGTTCGTGAAATCTCACGGCT  610
 MtuberculosisH37Rv GGGTGCGAGCGTTGTCCGGAATTACTGGGCGTAAAGAGCTCGTAGGTGGTTTGTCGCGTTGTTCGTGAAATCTCACGGCT  610
      MbovisPasteur GGGTGCGAGCGTTGTCCGGAATTACTGGGCGTAAAGAGCTCGTAGGTGGTTTGTCGCGTTGTTCGTGAAATCTCACGGCT  610
MtuberculosisCDC1551 GGGTGCGAGCGTTGTCCGGAATTACTGGGCGTAAAGAGCTCGTAGGTGGTTTGTCGCGTTGTTCGTGAAATCTCACGGCT  609
          Mulcerans GGGTGCGAGCGTTGTCCGGAATTACTGGGCGTAAAGAGCTCGTAGGTGGTTTGTCGCGTTGTTCGTGAAATCTCACGGCT  608
             Mavium GGGTGCGAGCGTTGTCCGGAATTACTGGGCGTAAAGAGCTCGTAGGTGGTTTGTCGCGTTGTTCGTGAAATCTCACGGCT  596
         MaviumK-10 GGGTGCGAGCGTTGTCCGGAATTACTGGGCGTAAAGAGCTCGTAGGTGGTTTGTCGCGTTGTTCGTGAAATCTCACGGCT  608
          MlepraeTN GGGTGCGAGCGTTGTCCGGAATTACTGGGCGTAAAGAGCTCGTAGGTGGTTTGTCGCGTTGTTCGTGAAATCTCACGGCT  621
               MJLS GGGTCCGAGCGTTGTCCGGAATTACTGGGCGTAAAGAGCTCGTAGGTGGTTTGTCGCGTTGTTCGTGAAAACCGGGGGCT  586
               MKMS GGGTCCGAGCGTTGTCCGGAATTACTGGGCGTAAAGAGCTCGTAGGTGGTTTGTCGCGTTGTTCGTGAAAACCGGGGGCT  586
               MMCS GGGTCCGAGCGTTGTCCGGAATTACTGGGCGTAAAGAGCTCGTAGGTGGTTTGTCGCGTTGTTCGTGAAAACCGGGGGCT  587
         Msmegmatis GGGTCCGAGCGTTGTCCGGAATTACTGGGCGTAAAGAGCTCGTAGGTGGTTTGTCGCGTTGTTCGTGAAAACTCACAGCT  599
       Mvanbaalenii GGGTCCGAGCGTTGTCCGGAATTACTGGGCGTAAAGAGCTCGTAGGTGGTTTGTCGCGTTGTTCGTGAAAACTCACAGCT  586
         Nfarcinica GGGTGCGAGCGTTGTCCGGAATTACTGGGCGTAAAGAGCTTGTAGGCGGTTTGTCGCGTCGTCCGTGAAAACTTGGGGCT  594
              RRHA1 GGGTGCAAGCGTTGTCCGGAATTACTGGGCGTAAAGAGTTCGTAGGCGGTTTGTCGCGTCGTTTGTGAAAACTCACAGCT  593
       Cdiphtheriae GGGTGCGAGCGTTGTCCGGAATTACTGGGCGTAAAGAGCTCGTAGGTGGTTTGTCGCGTCGTCTGTGAAATTCCGGGGCT  565
          Cjeikeium GGGTGCGAGCGTTGTCCGGAATTACTGGGCGTAAAGAGCTCGTAGGTGGTTTGTCGCGTCGTCTGTGAAATCCCGGGGCT  600
         Cefficiens GGGTGCGAGCGTTGTCCGGAATTACTGGGCGTAAAGAGCTCGTAGGTGGTTTGTCGCGTCGTCTGTGAAATCCCGGGGCT  576
        Cglutamicum GGGTGCGAGCGTTGTCCGGAATTACTGGGCGTAAAGAGCTCGTAGGTGGTTTGTCGCGTCGTCTGTGAAATCCCGGGGCT  596
       Nocardioides GGGTGCGAGCGTTGTCCGGAATTATTGGGCGTAAAGGGCTCGTAGGCGGTTTGTCGCGTCGGGAGTGAAAACACTGGGCT  596
             Pacnes GGGTGCGAGCGTTGTCCGGATTTATTGGGCGTAAAGGGCTCGTAGGTGGTTGATCGCGTCGGAAGTGTAATCTTGGGGCT  590
           TfuscaYX GGGTCCGAGCGTTGTCCGGAATTATTGGGCGTAAAGAGCTCGTAGGCGGCCTGTCGCGTCTGCTGTGAAAGTCCGGGGCT  582
      Rxylanophilus GGGGGCGAGCGTTGTCCGGAATCATTGGGCGTAAAGAGCGTGTAGGCGGCCCGGTAAGTCTGCTGTGAAAACCCGGGGCT  596
              ruler .......570.......580.......590.......600.......610.......620.......630.......640
```

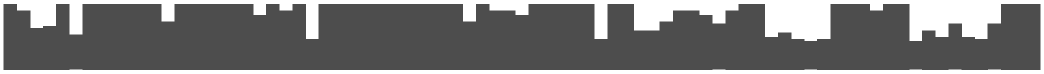

```
                      ***         **    *  ** *      ** ***      ** ** **  ******** **** * ***** ****
       Badolescentis TAACGGTGGATCCGCGCCGGGTACGGGCG-GGCTTGAGTGCGGTAGGGGAGACTGGAATTCCCGGTGTAACGGTGGAATG   684
             Blongum TAACGGTGGATCCGCGCCGGGTACGGGCG-GGCTTGAGTGCGGTAGGGGAGACTGGAATTCCCGGTGTAACGGTGGAATG   678
          Aaurescens CAACTCCGGTTCTGCAGTGGGTACGGGCA-GACTAGAGTGCAGTAGGGGAGACTGGAATTCCTGGTGTAGCGGTGAAATG   652
               AFB24 CAACTCCGGATCTGCGGTGGGTACGGGCA-GACTAGAGTGATGTAGGGGAGACTGGAATTCCTGGTGTAGCGGTGAAATG   666
              TTwist CAACCTCGGACCTGCGGTGGGTACGGGCA-GGCTAGAGTGCGATAGGGGAGAGCGGAATTCCTGGTGTAGCGGTGAAATG   678
            TTW08/27 CAACCTCGGACCTGCGGTGGGTACGGGCA-GGCTAGAGTGCGATAGGGGAGAGCGGAATTCCTGGTGTAGCGGTGAAATG   678
               Lxyli CAACCTCGGGCCTGCAGTGGGTACGGGCA-GACTAGAGTGCGGTAGGGGAGAATGGAATTCCTGGTGTAGCGGTGGAATG   647
        Savermitilis TAACCCCGGGTCTGCATTCGATACGGGCT-AGCTAGAGTGTGGTAGGGGAGATCGGAATTCCTGGTGTAGCGGTGAAATG   670
         Scoelicolor TAACCCCGGGTCTGCAGTCGATACGGGCA-GGCTAGAGTTCGGTAGGGGAGATCGGAATTCCTGGTGTAGCGGTGAAATG   673
        FalniACN14a CAACCCCGGGCCTGCAGTCGATACGGGCA-GGCTAGAGTCCGGCAGGGGAGACTGGAATTCCTGGTGTAGCGGTGAAATG   664
               FCcI3 CAACTCCGGGCCTGCAGTCGATACGGGCA-GGCTAGAGTCCGGCAGGGGAGACTGGAATTCCTGGTGTAGCGGTGAAATG   664
           FEAN1pec CAACCCCGGGCCTGCAGTCGATACGGGCA-GGCTAGAGTCCGGTAGGGGAGACTGGAATTCCTGGTGTAGCGGTGAAATG   662
      Acellulolyticus CAACCCTGGGCCTGCATTCGATACGGGCA-GACTAGAGTGCGGCAGGGGAGACTGGAATTCCTGGTGTAGCGGTGAAATG   661
      MbovisAF2122/97 TAACTGTGAGCGTGCGGGCGATACGGGCA-GACTAGAGTACTGCAGGGGAGACTGGAATTCCTGGTGTAGCGGTGGAATG   689
 MtuberculosisH37Rv TAACTGTGAGCGTGCGGGCGATACGGGCA-GACTAGAGTACTGCAGGGGAGACTGGAATTCCTGGTGTAGCGGTGGAATG   689
       MbovisPasteur TAACTGTGAGCGTGCGGGCGATACGGGCA-GACTAGAGTACTGCAGGGGAGACTGGAATTCCTGGTGTAGCGGTGGAATG   689
MtuberculosisCDC1551 TAACTGTGAGCGTGCGGGCGATACGGGCA-GACTAGAGTACTGCAGGGGAGACTGGAATTCCTGGTGTAGCGGTGGAATG   688
           Mulcerans TAACTGTGAGCGTGCGGGCGATACGGGCA-GACTAGAGTACTGCAGGGGAGACTGGAATTCCTGGTGTAGCGGTGGAATG   687
              Mavium TAACTGTGAGCGTGCGGGCGATACGGGCA-GACTAGAGTACTGCAGGGGAGACTGGAATTCCTGGTGTAGCGGTGGAATG   675
          MaviumK-10 TAACTGTGAGCGTGCGGGCGATACGGGCA-GACTAGAGTACTGCAGGGGAGACTGGAATTCCTGGTGTAGCGGTGGAATG   687
          MlepraeTN TAACTGTGAGCGTGCGGGCGATACGGGCA-GACTGGAGTACTGCAGGGGAGACTGGAATTCCTGGTGTAGCGGTGGAATG   700
                MJLS TAACCCTCGGCGTGCGGGCGATACGGGCA-GACTTGAGTACTGCAGGGGAGACTGGAATTCCTGGTGTAGCGGTGGAATG   665
                MKMS TAACCCTCGGCGTGCGGGCGATACGGGCA-GACTTGAGTACTGCAGGGGAGACTGGAATTCCTGGTGTAGCGGTGGAATG   665
                MMCS TAACCCTCGGCGTGCGGGCGATACGGGCA-GACTTGAGTACTGCAGGGGAGACTGGAATTCCTGGTGTAGCGGTGGAATG   666
          Msmegmatis TAACTGTGGGCGTGCGGGCGATACGGGCA-GACTAGAGTACTGCAGGGGAGACTGGAATTCCTGGTGTAGCGGTGGAATG   678
        Mvanbaalenii CAACTGTGGGCGTGCGGGCGATACGGGCA-GACTGGAGTACTGCAGGGGAGACTGGAATTCCTGGTGTAGCGGTGGAATG   665
          Nfarcinica CAACCCCAAGCTTGCGGGCGATACGGGCA-GACTTGAGTACTGCAGGGGAGACTGGAATTCCTGGTGTAGCGGTGAAATG   673
               RRHA1 CAACTGTGAGCCTGCAGGCGATACGGGCA-GACTTGAGTACTGCAGGGGAGACTGGAATTCCTGGTGTAGCGGTGAAATG   672
        Cdiphtheriae TAACTTCGGGCGTGCAGGCGATACGGGCATAACTTGAGTGCTGTAGGGGAGACTGGAATTCCTGGTGTAGCGGTGGAATG   645
           Cjeikeium TAACTTCGGGCGTGCAGGCGATACGGGCATAACTAGAGTGCTGTAGGGGAGACTGGAATTCCTGGTGTAGCGGTGAAATG   680
          Cefficiens TAACTTCGGGCGTGCAGGCGATACGGGCATAACTTGAGTGCTGTAGGGGAGACTGGAATTCCTGGTGTAGCGGTGAAATG   656
         Cglutamicum TAACTTCGGGCGTGCAGGCGATACGGGCATAACTTGAGTGCTGTAGGGGAGACTGGAATTCCTGGTGTAGCGGTGAAATG   676
        Nocardioides TAACTCAGTGCTTGCTTTCGATACGGGCA-GACTAGAGGCATGCAGGGGAGAACGGAATTCCTGGTGTAGCGGTGAAATG   675
              Pacnes TAACCCTGAGCGTGCTTTCGATACGGGTT-GACTTGAGGAAGGTAGGGGAGAATGGAATTCCTGGTGGAGCGGTGGAATG   669
            TfuscaYX TAACTCCGGGTGTGCAGTGGATACGGGCA-GGCTTGAGGTAGGTAGGGGAGACTGGAATTCCTGGTGTAGCGGTGAAATG   661
       Rxylanophilus CAACCCCGGGCGTGCAGTGGAAACTGCCG-GGCTAGAGGGCGGCAGAGGCGAGTGGAATTCCCGGTGTAGCGGTGAAATG   675
               ruler .......650.......660.......670.......680.......690.......700.......710.......720
```

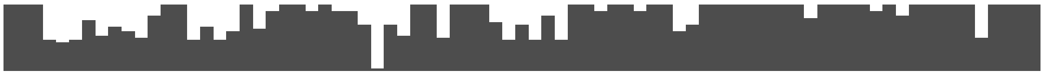

```
                      * ******* *** *******  * ******** * ** *****      ******** **   ******   *** **
       Badolescentis TGTAGATATCGGGAAGAACACCAATGGCGAAGGCAGGTCTCTGGGCCGTCACTGACGCTGAGGAGCGAAAGCGTGGGGAG   764
             Blongum TGTAGATATCGGGAAGAACACCAATGGCGAAGGCAGGTCTCTGGGCCGTTACTGACGCTGAGGAGCGAAAGCGTGGGGAG   758
          Aaurescens CGCAGATATCAGGAGGAACACCGATGGCGAAGGCAGGTCTCTGGGCTGTAACTGACGCTGAGGAGCGAAAGCATGGGGAG   732
               AFB24 CGCAGATATCAGGAGGAACACCGATGGCGAAGGCAGGTCTCTGGGCATTAACTGACGCTGAGGAGCGAAAGCATGGGGAG   746
              TTwist CGCAGATATCAGGAGGAACACCGATGGCGAAGGCAGCTCTCTGGGTCGCTACTGACGCTGAGGAGCGAAAGCGTGGGGAG   758
            TTW08/27 CGCAGATATCAGGAGGAACACCGATGGCGAAGGCAGCTCTCTGGGTCGCTACTGACGCTGAGGAGCGAAAGCGTGGGGAG   758
               Lxyli CGCAGATATCAGGAGGAACACCGATGGCGAAGGCAGTTCTCTGGGCCGTAACTGACGCTGAGGAGCGAAAGCGTGGGGAG   727
        Savermitilis CGCAGATATCAGGAGGAACACCGGTGGCGAAGGCGGATCTCTGGGCCATTACTGACGCTGAGGAGCGAAAGCGTGGGGAG   750
         Scoelicolor CGCAGATATCAGGAGGAACACCGGTGGCGAAGGCGGATCTCTGGGCCGATACTGACGCTGAGGAGCGAAAGCGTGGGGAG   753
        FalniACN14a CGCAGATATCAGGAGGAACACCGGTGGCGAAGGCGGGTCTCTGGGCCGGAACTGACGCTAAGGAGCGAAAGCGTGGGGAG   744
               FCcI3 CGCAGATATCAGGAGGAACACCGGTGGCGAAGGCGGGTCTCTGGGCCGGAACTGACGCTAAGGAGCGAAAGCGTGGGGAG   744
           FEAN1pec CGCAGATATCAGGAGGAACACCGGTGGCGAAGGCGGGTCTCTGGGCCGGAACTGACGCTAAGGAGCGAAAGCGTGGGGAG   742
      Acellulolyticus CGCAGATATCAGGAGGAACACCGGTGGCGAAGGCGGGTCTCTGGGCCGCAACTGACGCTGAGGAGCGAAAGCGTGGGGAG   741
      MbovisAF2122/97 CGCAGATATCAGGAGGAACACCGGTGGCGAAGGCGGGTCTCTGGGCAGTAACTGACGCTGAGGAGCGAAAGCGTGGGGAG   769
 MtuberculosisH37Rv CGCAGATATCAGGAGGAACACCGGTGGCGAAGGCGGGTCTCTGGGCAGTAACTGACGCTGAGGAGCGAAAGCGTGGGGAG   769
       MbovisPasteur CGCAGATATCAGGAGGAACACCGGTGGCGAAGGCGGGTCTCTGGGCAGTAACTGACGCTGAGGAGCGAAAGCGTGGGGAG   769
MtuberculosisCDC1551 CGCAGATATCAGGAGGAACACCGGTGGCGAAGGCGGGTCTCTGGGCAGTAACTGACGCTGAGGAGCGAAAGCGTGGGGAG   768
           Mulcerans CGCAGATATCAGGAGGAACACCGGTGGCGAAGGCGGGTCTCTGGGCAGTAACTGACGCTGAGGAGCGAAAGCGTGGGGAG   767
              Mavium CGCAGATATCAGGAGGAACACCGGTGGCGAAGGCGGGTCTCTGGGCAGTAACTGACGCTGAGGAGCGAAAGCGTGGGGAG   755
          MaviumK-10 CGCAGATATCAGGAGGAACACCGGTGGCGAAGGCGGGTCTCTGGGCAGTAACTGACGCTGAGGAGCGAAAGCGTGGGGAG   767
          MlepraeTN CGCAGATATCAGGAGGAACACCAGTGGCGAAGGCGGGTCTCTGGGCAGTAACTGACGCTGAGGAGCGAAAGCGTGGGGAG   780
                MJLS CGCAGATATCAGGAGGAACACCGGTGGCGAAGGCGGGTCTCTGGGCAGTAACTGACGCTGAGGAGCGAAAGCGTGGGGAG   745
                MKMS CGCAGATATCAGGAGGAACACCGGTGGCGAAGGCGGGTCTCTGGGCAGTAACTGACGCTGAGGAGCGAAAGCGTGGGGAG   745
                MMCS CGCAGATATCAGGAGGAACACCGGTGGCGAAGGCGGGTCTCTGGGCAGTAACTGACGCTGAGGAGCGAAAGCGTGGGGAG   746
          Msmegmatis CGCAGATATCAGGAGGAACACCGGTGGCGAAGGCGGGTCTCTGGGCAGTAACTGACGCTGAGGAGCGAAAGCGTGGGGAG   758
        Mvanbaalenii CGCAGATATCAGGAGGAACACCGGTGGCGAAGGCGGGTCTCTGGGCAGTAACTGACGCTGAGGAGCGAAAGCGTGGGGAG   745
          Nfarcinica CGCAGATATCAGGAGGAACACCGGTGGCGAAGGCGGGTCTCTGGGCAGTAACTGACGCTGAGAAGCGAAAGCGTGGGTAG   753
               RRHA1 CGCAGATATCAGGAGGAACACCGGTGGCGAAGGCGGGTCTCTGGGCAGTAACTGACGCTGAGGAACGAAAGCGTGGGTAG   752
        Cdiphtheriae CGCAGATATCAGGAGGAACACCGATGGCGAAGGCAGGTCTCTGGGCAGTAACTGACGCTGAGGAGCGAAAGCATGGGGAG   725
           Cjeikeium CGCAGATATCAGGAGGAACACCGATGGCGAAGGCAGGTCTCTGGGCAGTTACTGACGCTGAGGAGCGAAAGCATGGGTAG   760
          Cefficiens CGCAGATATCAGGAGGAACACCAATGGCGAAGGCAGGTCTCTGGGCAGTAACTGACGCTGAGGAGCGAAAGCATGGGTAG   736
         Cglutamicum CGCAGATATCAGGAGGAACACCAATGGCGAAGGCAGGTCTCTGGGCAGTAACTGACGCTGAGGAGCGAAAGCATGGGTAG   756
        Nocardioides CGCAGATATCAGGAGGAACACCGGTGGCGAAGGCGGTTCTCTGGGCATGTCCTGACGCTGAGGAGCGAAAGTGTGGGGAG   755
              Pacnes CGCAGATATCAGGAGGAACACCAGTGGCGAAGGCGGTTCTCTGGGCCTTTCCTGACGCTGAGGAGCGAAAGCGTGGGGAG   749
            TfuscaYX CGCAGATATCAGGAGGAACACCGGTGGCGAAGGCGGGTCTCTGGGCCTTACCTGACGCTGAGGAGCGAAAGCGTGGGGAG   741
       Rxylanophilus CGCAGATATCGGGAGGAACACCAGTAGCGAAGGCGGCTCGCTGGGCCGTTCCTGACGCTGAGACGCGAAAGCTAGGGGAG   755
               ruler .......730.......740.......750.......760.......770.......780.......790.......800
```

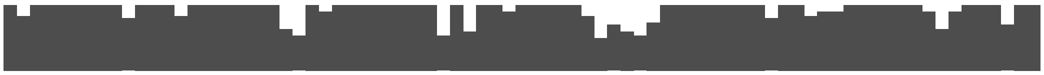

```
                    * ****** *******************   * ******* ***   ** *  **  *     *             * *
Badolescentis       CGAACAGGATTAGATACCCTGGTAGTCCACGCCGTAAACGGTGGATGCTGGATGTGGGGACCAT-TCCACGGTCTCCGTG  843
Blongum             CGAACAGGATTAGATACCCTGGTAGTCCACGCCGTAAACGGTGGATGCTGGATGTGGGGCCCGT-TCCACGGGTTCCGTG  837
Aaurescens          CGAACAGGATTAGATACCCTGGTAGTCCATGCCGTAAACGTTGGGCACTAGGTGTGGGGGACAT-TCCACGTTTTCCGCG  811
AFB24               CGAACAGGATTAGATACCCTGGTAGTCCATGCCGTAAACGTTGGGCACTAGGTGTGGGGGACAT-TCCACGTTTTCCGCG  825
TTwist              CGAACAGGATTAGATACCCTGGTAGTCCACGCCGTAAACGTTGGGCGCTAGACGTGGGGGTGGT-TCCACCGCTTCCGTG  837
TTW08/27            CGAACAGGATTAGATACCCTGGTAGTCCACGCCGTAAACGTTGGGCGCTAGACGTGGGGGTGGT-TCCACCGCTTCCGTG  837
Lxyli               CGAACAGGATTAGATACCCTGGTAGTCCACGCCGTAAACGTTGGGCGCTAGATGTGGGGACCAT-TCCACGGTTTCCGTG  806
Savermitilis        CGAACAGGATTAGATACCCTGGTAGTCCACGCCGTAAACGGTGGGAACTAGGTGTTGGCGACAT-TCCACGTCGTCGGTG  829
Scoelicolor         CGAACAGGATTAGATACCCTGGTAGTCCACGCCGTAAACGGTGGGCACTAGGTGTGGGCAACAT-TCCACGTTGTCCGTG  832
FalniACN14a         CGAACAGGATTAGATACCCTGGTAGTCCACGCCGTAAACGTTGGGCGCTAGGTGTGGGGGACCT-TCCACGGCCTCCGTG  823
FCcI3               CGAACAGGATTAGATACCCTGGTAGTCCACGCCGTAAACGTTGGGCGCTAGGTGTGGGGGACCT-TCCACGGCCTCCGTG  823
FEAN1pec            CGAACAGGATTAGATACCCTGGTAGTCCACGCCGTAAACGTTGGGCGCTAGGTGTGGGGGACCT-TCCACGGCCTCCGTG  821
Acellulolyticus     CGAACAGGATTAGATACCCTGGTAGTCCACGCTGTAAACGTTGGGCGCTAGGTGTGGGGGACTT-TCCACGTCCTCCGTG  820
MbovisAF2122/97     CGAACAGGATTAGATACCCTGGTAGTCCACGCCGTAAACGGTGGGTACTAGGTGTGGGTTTCCT-TCCTTGGGATCCGTG  848
MtuberculosisH37Rv  CGAACAGGATTAGATACCCTGGTAGTCCACGCCGTAAACGGTGGGTACTAGGTGTGGGTTTCCT-TCCTTGGGATCCGTG  848
MbovisPasteur       CGAACAGGATTAGATACCCTGGTAGTCCACGCCGTAAACGGTGGGTACTAGGTGTGGGTTTCCT-TCCTTGGGATCCGTG  848
MtuberculosisCDC1551 CGAACAGGATTAGATACCCTGGTAGTCCACGCCGTAAACGGTGGGTACTAGGTGTGGGTTTCCT-TCCTTGGGATCCGTG  847
Mulcerans           CGAACAGGATTAGATACCCTGGTAGTCCACGCCGTAAACGGTGGGTACTAGGTGTGGGTTTCCT-TCCTTGGGATCCGTG  846
Mavium              CGAACAGGATTAGATACCCTGGTAGTCCACGCCGTAAACGGTGGGTACTAGGTGTGGGTTTCCT-TCCTTGGGATCCGTG  834
MaviumK-10          CGAACAGGATTAGATACCCTGGTAGTCCACGCCGTAAACGGTGGGTACTAGGTGTGGGTTTCCT-TCCTTGGGATCCGTG  846
MlepraeTN           CAAACAGGATTAGATACCCTGGTAGTCCACGCCGTAAACGGTGGGTACTAGGTGTGGGTTTCCT-TCCTTGGGATCCGTG  859
MJLS                CGAACAGGATTAGATACCCTGGTAGTCCACGCCGTAAACGGTGGGTACTAGGTGTGGGTTTCCT-TCCTTGGGATCCGTG  824
MKMS                CGAACAGGATTAGATACCCTGGTAGTCCACGCCGTAAACGGTGGGTACTAGGTGTGGGTTTCCT-TCCTTGGGATCCGTG  824
MMCS                CGAACAGGATTAGATACCCTGGTAGTCCACGCCGTAAACGGTGGGTACTAGGTGTGGGTTTCCT-TCCTTGGGATCCGTG  825
Msmegmatis          CGAACAGGATTAGATACCCTGGTAGTCCACGCCGTAAACGGTGGGTACTAGGTGTGGGTTTCCT-TCCTTGGGATCCGTG  837
Mvanbaalenii        CGAACAGGATTAGATACCCTGGTAGTCCACGCCGTAAACGGTGGGTACTAGGTGTGGGTTTCCT-TCCTTGGGATCCGTG  824
Nfarcinica          CGAACAGGATTAGATACCCTGGTAGTCCACGCCGTAAACGGTGGGCGCTAGGTGTGGGTTTCCT-TCCACGGGATCCGTG  832
RRHA1               CAAACAGGATTAGATACCCTGGTAGTCCACGCCGTAAACGGTGGGCGCTAGGTGTGGGTT-CCT-TCCACGGGATCTGTG  830
Cdiphtheriae        CGAACAGGATTAGATACCCTGGTAGTCCATGCCGTAAACGGTGGGCGCTAGGTGTGAGGGTCTT--CCACGACTTTCGTG  803
Cjeikeium           CGAACAGGATTAGATACCCTGGTAGTCCATGCCGTAAACGGTGGGCGCTAGGTGTGGGGGTTTTATTTACGATTCCCGTG  840
Cefficiens          CGAACAGGATTAGATACCCTGGTAGTCCATGCCGTAAACGGTGGGCGCTAGGTGTAGGGGACTT--CCACGTCTTCTGTG  814
Cglutamicum         CGAACAGGATTAGATACCCTGGTAGTCCATGCCGTAAACGGTGGGCGCTAGGTGTAGGGGTCTT--CCACGACTTCTGTG  834
Nocardioides        CGAACAGGATTAGATACCCTGGTAGTCCACACCGTAAACGTTGGGCGCTAGGTGTGGGATCCAT-TCCACGGGTTCCGTG  834
Pacnes              CGAACAGGCTTAGATACCCTGGTAGTCCACGCTGTAAACGGTGGGTACTAGGTGTGGGGTCCAT-TCCACGGGTTCCGTG  828
TfuscaYX            CGAACAGGATTAGATACCCTGGTAGTCCACGCCGTAAACGTTGGGCGCTAGGTGTGGGGACTTT--CCACGGTTTCCGTG  819
Rxylanophilus       CGAACAGGATTAGATACCCTGGTAGTCCTAGCCGTAAACGATGGGCACTAGGTGTGGGGGGAGT--CGAATCCCTCCGTG  833
ruler               .......810.......820.......830.......840.......850.......860.......870.......880
```

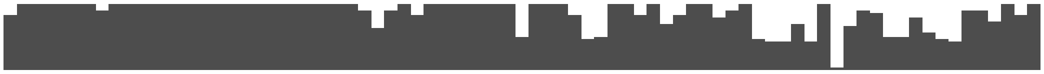

```
                     ** *** ***** *****   *************************************   *********** ******
Badolescentis       TCGGAGCCAACGCGTTAAGCATCCCGCCTGGGGAGTACGGCCGCAAGGCTAAAACTCAAAGAAATTGACGGGGGCCCGCA  923
Blongum             TCGGAGCTAACGCGTTAAGCATCCCGCCTGGGGAGTACGGCCGCAAGGCTAAAACTCAAAGAAATTGACGGGGGCCCGCA  917
Aaurescens          CCGTAGCTAACGCATTAAGTGCCCCGCCTGGGGAGTACGGCCGCAAGGCTAAAACTCAAAGGAATTGACGGGGGCCCGCA  891
AFB24               CCGTAGCTAACGCATTAAGTGCCCCGCCTGGGGAGTACGGCCGCAAGGCTAAAACTCAAAGGAATTGACGGGGGCCCGCA  905
TTwist              TCGCAGCTAACGCATTAAGCGCCCCGCCTGGGGAGTACGGCCGCAAGGCTAAAACTCAAAGGAATTGACGGGGGCCCGCA  917
TTW08/27            TCGCAGCTAACGCATTAAGCGCCCCGCCTGGGGAGTACGGCCGCAAGGCTAAAACTCAAGGGAATTGACGGGGGCCCGCA  917
Lxyli               TCGCAGCTAACGCATTAAGCGCCCCGCCTGGGGAGTACGGCCGCAAGGCTAAAACTCAAAGGAATTGACGGGGGCCCGCA  886
Savermitilis        CCGCAGCTAACGCATTAAGTTCCCCGCCTGGGGAGTACGGCCGCAAGGCTAAAACTCAAAGGAATTGACGGGGGCCCGCA  909
Scoelicolor         CCGCAGCTAACGCATTAAGTGCCCCGCCTGGGGAGTACGGCCGCAAGGCTAAAACTCAAAGGAATTGACGGGGGCCCGCA  912
FalniACN14a         CCGCAGCTAACGCATTAAGCGCCCCGCCTGGGGAGTACGGCCGCAAGGCTAAAACTCAAAGGAATTGACGGGGGCCCGCA  903
FCcI3               CCGCAGCTAACGCATTAAGCGCCCCGCCTGGGGAGTACGGCCGCAAGGCTAAAACTCAAAGGAATTGACGGGGGCCCGCA  903
FEAN1pec            CCGCAGCTAACGCATTAAGCGCCCCGCCTGGGGAGTACGGCCGCAAGGCTAAAACTCAAAGGAATTGACGGGGGCCCGCA  901
Acellulolyticus     CCGCAGCTAACGCATTAAGCGCCCCGCCTGGGGAGTACGGCCGCAAGGCTAAAACTCAAAGGAATTGACGGGGGCCCGCA  900
MbovisAF2122/97     CCGTAGCTAACGCATTAAGTACCCCGCCTGGGGAGTACGGCCGCAAGGCTAAAACTCAAAGGAATTGACGGGGGCCCGCA  928
MtuberculosisH37Rv  CCGTAGCTAACGCATTAAGTACCCCGCCTGGGGAGTACGGCCGCAAGGCTAAAACTCAAAGGAATTGACGGGGGCCCGCA  928
MbovisPasteur       CCGTAGCTAACGCATTAAGTACCCCGCCTGGGGAGTACGGCCGCAAGGCTAAAACTCAAAGGAATTGACGGGGGCCCGCA  928
MtuberculosisCDC1551 CCGTAGCTAACGCATTAAGTACCCCGCCTGGGGAGTACGGCCGCAAGGCTAAAACTCAAAGGAATTGACGGGGGCCCGCA  927
Mulcerans           CCGTAGCTAACGCATTAAGTACCCCGCCTGGGGAGTACGGCCGCAAGGCTAAAACTCAAAGGAATTGACGGGGGCCCGCA  926
Mavium              CCGTAGCTAACGCATTAAGTACCCCGCCTGGGGAGTACGGCCGCAAGGCTAAAACTCAAAGGAATTGACGGGGGCCCGCA  914
MaviumK-10          CCGTAGCTAACGCATTAAGTACCCCGCCTGGGGAGTACGGCCGCAAGGCTAAAACTCAAAGGAATTGACGGGGGCCCGCA  926
MlepraeTN           CCGTAGCTAACGCATTAAGTACCCCGCCTGGGGAGTACGGCCGCAAGGCTAAAACTCAAAGGAATTGACGGGGGCCCGCA  939
MJLS                CCGTAGCTAACGCATTAAGTACCCCGCCTGGGGAGTACGGCCGCAAGGCTAAAACTCAAAGAAATTGACGGGGGCCCGCA  904
MKMS                CCGTAGCTAACGCATTAAGTACCCCGCCTGGGGAGTACGGCCGCAAGGCTAAAACTCAAAGAAATTGACGGGGGCCCGCA  904
MMCS                CCGTAGCTAACGCATTAAGTACCCCGCCTGGGGAGTACGGCCGCAAGGCTAAAACTCAAAGAAATTGACGGGGGCCCGCA  905
Msmegmatis          CCGTAGCTAACGCATTAAGTACCCCGCCTGGGGAGTACGGCCGCAAGGCTAAAACTCAAAGGAATTGACGGGGGCCCGCA  917
Mvanbaalenii        CCGTAGCTAACGCATTAAGTACCCCGCCTGGGGAGTACGGCCGCAAGGCTAAAACTCAAAGAAATTGACGGGGGCCCGCA  904
Nfarcinica          CCGTAGCTAACGCATTAAGCGCCCCGCCTGGGGAGTACGGCCGCAAGGCTAAAACTCAAAGGAATTGACGGGGGCCCGCA  912
RRHA1               CCGTAGCTAACGCATTAAGCGCCCCGCCTGGGGAGTACGGCCGCAAGGCTAAAACTCAAAGGAATTGACGGGGGCCCGCA  910
Cdiphtheriae        CCGTAGCTAACGCATTAAGCGCCCCGCCTGGGGAGTACGGCCGCAAGGCTAAAACTCAAAGGAATTGACGGGGGCCCGCA  883
Cjeikeium           CCGTAGCTAACGCATTAAGCGCCCCGCCTGGGGAGTACGGCCGCAAGGCTAAAACTCAAAGGAATTGACGGGGGCCCGCA  920
Cefficiens          CCGCAGCTAACGCATTAAGCGCCCCGCCTGGGGAGTACGGCCGCAAGGCTAAAACTCAAAGGAATTGACGGGGGCCCGCA  894
Cglutamicum         CCGCAGCTAACGCATTAAGCGCCCCGCCTGGGGAGTACGGCCGCAAGGCTAAAACTCAAAGGAATTGACGGGGGCCCGCA  914
Nocardioides        CCGCAGCTAACGCATTAAGCGCCCCGCCTGGGGAGTACGGCCGCAAGGCTAAAACTCAAAGGAATTGACGGGGGCCCGCA  914
Pacnes              CCGTAGCTAACGCTTTAAGTACCCCGCCTGGGGAGTACGGCCGCAAGGCTAAAACTCAAAGGAATTGACGGGGCCCCGCA  908
TfuscaYX            CCGTAGCTAACGCATTAAGCGCCCCGCCTGGGGAGTACGGCCGCAAGGCTAAAACTCAAAGGAATTGACGGGGGCCCGCA  899
Rxylanophilus       CCGCAGCCAACGCGTTAAGTGCCCCGCCTGGGGAGTACGGCCGCAAGGCTAAAACTCAAAAGAATTGACGGGGGCCCGCA  913
ruler               .......890.......900.......910.......920.......930.......940.......950.......960
```

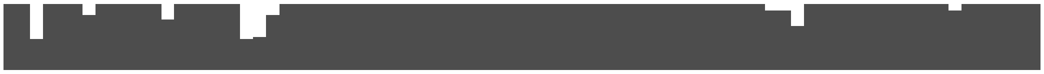

```
                      ***** **********    ******* * ******** ***********  ** **** **                 *
        Badolescentis CAAGCGGCGGAGCATGCGGATTAATTCGATGCAACGCGAAGAACCTTACCTGGGCTTGACATGT-TCCCGACAGCCCCAG 1002
              Blongum CAAGCGGCGGAGCATGCGGATTAATTCGATGCAACGCGAAGAACCTTACCTGGGCTTGACATGT-TCCCGACGGTCGTAG  996
           Aaurescens CAAGCGGCGGAGCATGCGGATTAATTCGATGCAACGCGAAGAACCTTACCAAGGCTTGACATGA-ACCGGAAAGACCTGG  970
                AFB24 CAAGCGGCGGAGCATGCGGATTAATTCGATGCAACGCGAAGAACCTTACCAAGGCTTGACATGG-GCCGGACCGGGCTGG  984
               TTwist CAAGCGGCGGAGCATGCGGATTAATTCAATGCAACGCGAAGAACCTTACCAAGGCTTGATATAT-ACCGGAAAGGCGTAG  996
             TTW08/27 CAAGCGGCGGAGCATGCGGATTAATTCAATGCAACGCGAAGAACCTTACCAAGGCTTGATATAT-ACCGGAAAGGCGTAG  996
                Lxyli CAAGCGGCGGAGCATGCGGATTAATTCGATGCAACGCGAAGAACCTTACCAAGGCTTGACATAT-ACGAGAACGGGCTAG  965
         Savermitilis CAAGCAGCGGAGCATGTGGCTTAATTCGACGCAACGCGAAGAACCTTACCAAGGCTTGACATAC-ACCGGAAAGCATTAG  988
          Scoelicolor CAAGCGGCGGAGCATGTGGCTTAATTCGACGCAACGCGAAGAACCTTACCAAGGCTTGACATAC-ACCGGAAAGCATCAG  991
          FalniACN14a CAAGCGGCGGAGCATGTGGCTTAATTCGATGCAACGCGAAGAACCTTACCAGGGCTTGACATGC-AGGGAAATCTCGTAG  982
                FCcI3 CAAGCGGCGGAGCATGTGGCTTAATTCGATGCAACGCGAAGAACCTTACCAGGGCTTGACATGC-AGGGAAATCTCGTAG  982
             FEAN1pec CAAGCGGCGGAGCATGTGGCTTAATTCGATGCAACGCGAAGAACCTTACCAGGGCTTGACATGC-AGAGAAATCCTGTAG  980
      Acellulolyticus CAAGCGGCGGAGCATGTGGCTTAATTCGATGCAACGCGAAGAACCTTACCTGGGCTTGACATGC-AGGGAAATCCGGCAG  979
       MbovisAF2122/97 CAAGCGGCGGAGCATGTGGATTAATTCGATGCAACGCGAAGAACCTTACCTGGGTTTGACATGC-ACAGGACGCGTCTAG 1007
   MtuberculosisH37Rv CAAGCGGCGGAGCATGTGGATTAATTCGATGCAACGCGAAGAACCTTACCTGGGTTTGACATGC-ACAGGACGCGTCTAG 1007
        MbovisPasteur CAAGCGGCGGAGCATGTGGATTAATTCGATGCAACGCGAAGAACCTTACCTGGGTTTGACATGC-ACAGGACGCGTCTAG 1007
 MtuberculosisCDC1551 CAAGCGGCGGAGCATGTGGATTAATTCGATGCAACGCGAAGAACCTTACCTGGGTTTGACATGC-ACAGGACGCGTCTAG 1006
            Mulcerans CAAGCGGCGGAGCATGTGGATTAATTCGATGCAACGCGAAGAACCTTACCTGGGTTTGACATGC-ACAGGACGCGTCTAG 1005
               Mavium CAAGCGGCGGAGCATGTGGATTAATTCGATGCAACGCGAAGAACCTTACCTGGGTTTGACATGC-ACAGGACGCGTCTAG  993
           MaviumK-10 CAAGCGGCGGAGCATGTGGATTAATTCGATGCAACGCGAAGAACCTTACCTGGGTTTGACATGC-ACAGGACGCGTCTAG 1005
           MlepraeTN CAAGCGGCGGAGCATGTGGATTAATTCGATGCAACGCGAAGAACCTTACCTGGGTTTGACATGC-ACAGGATGCGTCTAG 1018
                 MJLS CAAGCGGCGGAGCATGTGGATTAATTCGATGCAACGCGAAGAACCTTACCTGGGTTTGACATGC-ACAGGACGCTGGTAG  983
                 MKMS CAAGCGGCGGAGCATGTGGATTAATTCGATGCAACGCGAAGAACCTTACCTGGGTTTGACATGC-ACAGGACGCTGGTAG  983
                 MMCS CAAGCGGCGGAGCATGTGGATTAATTCGATGCAACGCGAAGAACCTTACCTGGGTTTGACATGC-ACAGGACGCTGGTAG  984
           Msmegmatis CAAGCGGCGGAGCATGTGGATTAATTCGATGCAACGCGAAGAACCTTACCTGGGTTTGACATGC-ACAGGACGCCGGCAG  996
         Mvanbaalenii CAAGCGGCGGAGCATGTGGATTAATTCGATGCAACGCGAAGAACCTTACCTGGGTTTGACATGC-ACAGGACGTGCCTAG  983
           Nfarcinica CAAGCGGCGGAGCATGTGGATTAATTCGATGCAACGCGAAGAACCTTACCTGGGTTTGACATAC-ACCGGAAACCTGCAG  991
                RRHA1 CAAGCGGCGGAGCATGTGGATTAATTCGATGCAACGCGAAGAACCTTACCTGGGTTTGACATAT-ACCGGAAAGCCGTAG  989
         Cdiphtheriae CAAGCGGCGGAGCATGTGGATTAATTCGATGCAACGCGAAGAACCTTACCTGGGCTTGACATAT-GCAGGATCGGCGTAG  962
            Cjeikeium CAAGCGGCGGAGCATGTGGATTAATTCGATGCAACGCGAAGAACCTTACCTGGGCTTGACATAC-ACCGGATCGCTGCAG  999
           Cefficiens CAAGCGGCGGAGCATGTGGATTAATTCGATGCAACGCGAAGAACCTTACCTGGGCTTGACATAC-ACTAGATCGCTGCAG  973
          Cglutamicum CAAGCGGCGGAGCATGTGGATTAATTCGATGCAACGCGAAGAACCTTACCTGGGCTTGACATGG-ACCGGATCGGCGTAG  993
         Nocardioides CAAGCGGCGGAGCATGCGGATTAATTCGATGCAACGCGAAGAACCTTACCTGGGTTTGACATAC-GCCGGAAAGCGCCAG  993
               Pacnes CAAGCGGCGGAGCATGCGGATTAATTCGATGCAACGCGTAGAACCTTACCTGGGTTTGACATGG-ATCGGGAGTGCTCAG  987
             TfuscaYX CAAGCGGCGGAGCATGTTGCTTAATTCGACGCAACGCGAAGAACCTTACCAAGGCTTGACATCA-CCGGTAATCCTCCAG  978
        Rxylanophilus CAAGCAGCGGAGCATGTTCTTTAATTCGATGCAACGCGAAGAACCTTACCTGGGCTTGACATGCTGGTGGTAGGCGCCGG  993
                ruler .......970.......980.......990......1000......1010......1020......1030......1040
```

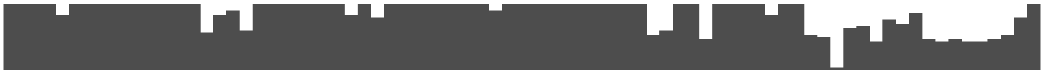

```
                        *                            ****** **** **  *********************************
        Badolescentis AGATGGG--GCCTCCCTTCGGGGCGGGTTCACAGGTGGTGCATGGTCGTCGTCAGCTCGTGTCGTGAGATGTTGGGTTAA 1080
              Blongum AGATACG--GCTTCCCTTCGGGGCGGGTTCACAGGTGGTGCATGGTCGTCGTCAGCTCGTGTCGTGAGATGTTGGGTTAA 1074
           Aaurescens AAACAGG-TGCCCCGCTT-GCGGTCGGTTTACAGGTGGTGCATGGTTGTCGTCAGCTCGTGTCGTGAGATGTTGGGTTAA 1048
                AFB24 AAACAGT-CCTTCCCCTTTGGGGCCGGTTCACAGGTGGTGCATGGTTGTCGTCAGCTCGTGTCGTGAGATGTTGGGTTAA 1063
               TTwist AGATA---CGCCCCCCGC-AAGGTCGGTATACAGGTGGTGCACGGTTGTCGTCAGCTCGTGTCGTGAGATGTTGGGTTAA 1072
             TTW08/27 AGATA---CGCCCCCCGC-AAGGTCGGTATACAGGTGGTGCACGGTTGTCGTCAGCTCGTGTCGTGAGATGTTGGGTTAA 1072
                Lxyli AAATAGT-CAACTCTTTG-GACACTCGTAAACAGGTGGTGCATGGTTGTCGTCAGCTCGTGTCGTGAGATGTTGGGTTAA 1043
         Savermitilis AGATAG--TGCCCCCCTT-GTGGTCGGTGTACAGGTGGTGCATGGCTGTCGTCAGCTCGTGTCGTGAGATGTTGGGTTAA 1065
          Scoelicolor AGATGG--TGCCCCCCTT-GTGGTCGGTGTACAGGTGGTGCATGGCTGTCGTCAGCTCGTGTCGTGAGATGTTGGGTTAA 1068
          FalniACN14a AGATACG---GGGTCCGTAAGGGTCC-TGCACAGGTGGTGCATGGCTGTCGTCAGCTCGTGTCGTGAGATGTTGGGTTAA 1058
                FCcI3 AGATACG---GGGTCCGTAAGGGTCC-TGCACAGGTGGTGCATGGCTGTCGTCAGCTCGTGTCGTGAGATGTTGGGTTAA 1058
             FEAN1pec AGATATG---GGGTCCTTAGGGGCTC-TGCACAGGTGGTGCATGGCTGTCGTCAGCTCGTGTCGTGAGATGTTGGGTTAA 1056
      Acellulolyticus AGATGCC---GGGTCCGCAAGGGCCC-TGCACAGGTGGTGCATGGCTGTCGTCAGCTCGTGTCGTGAGATGTTGGGTTAA 1055
      MbovisAF2122/97 AGATAGG---CGTTCCCTTGTGGCCTGTGTGCAGGTGGTGCATGGCTGTCGTCAGCTCGTGTCGTGAGATGTTGGGTTAA 1084
   MtuberculosisH37Rv AGATAGG---CGTTCCCTTGTGGCCTGTGTGCAGGTGGTGCATGGCTGTCGTCAGCTCGTGTCGTGAGATGTTGGGTTAA 1084
        MbovisPasteur AGATAGG---CGTTCCCTTGTGGCCTGTGTGCAGGTGGTGCATGGCTGTCGTCAGCTCGTGTCGTGAGATGTTGGGTTAA 1084
 MtuberculosisCDC1551 AGATAGG---CGTTCCCTTGTGGCCTGTGTGCAGGTGGTGCATGGCTGTCGTCAGCTCGTGTCGTGAGATGTTGGGTTAA 1083
            Mulcerans AGATAGG---CGTTCCCTTGTGGCCTGTGTGCAGGTGGTGCATGGCTGTCGTCAGCTCGTGTCGTGAGATGTTGGGTTAA 1082
               Mavium AGATAGG---CGTTCCCTTGTGGCCTGTGTGCAGGTGGTGCATGGCTGTCGTCAGCTCGTGTCGTGAGATGTTGGGTTAA 1070
           MaviumK-10 AGATAGG---CGTTCCCTTGTGGCCTGTGTGCAGGTGGTGCATGGCTGTCGTCAGCTCGTGTCGTGAGATGTTGGGTTAA 1082
            MlepraeTN AGATAGG---CACTCCCTTGTGGCCTGTGTGCAGGTGGTGCATGGCTGTCGTCAGCTCGTGTCGTGAGATGTTGGGTTAA 1095
                 MJLS AGATATC---AGTTCCCTTGTGGCCTGTGTGCAGGTGGTGCATGGCTGTCGTCAGCTCGTGTCGTGAGATGTTGGGTTAA 1060
                 MKMS AGATATC---AGTTCCCTTGTGGCCTGTGTGCAGGTGGTGCATGGCTGTCGTCAGCTCGTGTCGTGAGATGTTGGGTTAA 1060
                 MMCS AGATATC---AGTTCCCTTGTGGCCTGTGTGCAGGTGGTGCATGGCTGTCGTCAGCTCGTGTCGTGAGATGTTGGGTTAA 1061
           Msmegmatis AGATGTC---GGTTCCCTTGTGGCCTGTGTGCAGGTGGTGCATGGCTGTCGTCAGCTCGTGTCGTGAGATGTTGGGTTAA 1073
         Mvanbaalenii AGATAGG---TATTCCCTTGTGGCCTGTGTGCAGGTGGTGCATGGCTGTCGTCAGCTCGTGTCGTGAGATGTTGGGTTAA 1060
           Nfarcinica AGATGTA---GGCCCCCTTGTGGTCGGTGTACAGGTGGTGCATGGCTGTCGTCAGCTCGTGTCGTGAGATGTTGGGTTAA 1068
                RRHA1 AGATACG---GCCCCCCTTGTGGTCGGTATACAGGTGGTGCATGGCTGTCGTCAGCTCGTGTCGTGAGATGTTGGGTTAA 1066
         Cdiphtheriae TGATACG---TTTTCCCTTGTGGTCTGTATACAGGTGGTGCATGGTTGTCGTCAGCTCGTGTCGTGAGATGTTGGGTTAA 1039
            Cjeikeium AGATGTA---GTTTCCCTTGTGGCTGGTGTACAGGTGGTGCATGGTTGTCGTCAGCTCGTGTCGTGAGATGTTGGGTTAA 1076
           Cefficiens AGATGTA---GCTTCCCTTGTGGCTGGTGTACAGGTGGTGCATGGTTGTCGTCAGCTCGTGTCGTGAGATGTTGGGTTAA 1050
          Cglutamicum AGATACG---TTTTCCCTTGTGGTCGGTTCACAGGTGGTGCATGGTTGTCGTCAGCTCGTGTCGTGAGATGTTGGGTTAA 1070
         Nocardioides AGATGG---TGCCCCTTTTAG--TCGGTGTACAGGTGGTGCATGGCTGTCGTCAGCTCGTGTCGTGAGATGTTGGGTTAA 1068
               Pacnes AGATGGGTGTGCCTCTTTTGGGGTCGGTTCACAGGTGGTGCATGGCTGTCGTCAGCTCGTGTCGTGAGATGTTGGGTTAA 1067
             TfuscaYX AGATGGG--GGGTCCTTCGGGGATCGGTG-ACAGGTGGTGCATGGCTGTCGTCAGCTCGTGTCGTGAGATGTTGGGTTAA 1055
        Rxylanophilus AAACGGTGGCCGACCCTTCGGGGAGCCAGCACAGGTGCTGCATGGCTGTCGTCAGCTCGTGTCGTGAGATGTTGGGTTAA 1073
                ruler ......1050......1060......1070......1080......1090......1100......1110......1120
```

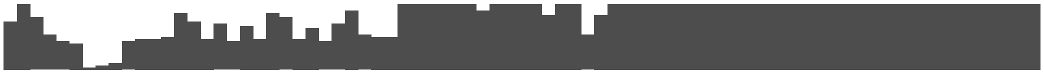

```
                     *********************        ******** *              *  *** ****    * *** ****
       Badolescentis GTCCCGCAACGAGCGCAACCCTCGCCCTGTGTTGCCAGCA-------CGTCGTGGTGGGAACTCACGGGGGACCGCCGGG 1153
             Blongum GTCCCGCAACGAGCGCAACCCTCGCCCCGTGTTGCCAGCG-------GATTATGCCGGGAACTCACGGGGGACCGCCGGG 1147
          Aaurescens GTCCCGCAACGAGCGCAACCCTCGTTCTATGTTGCCAGCG-------CGTTATGGCGGGGACTCATAGGAGACTGCCGGG 1121
               AFB24 GTCCCGCAACGAGCGCAACCCTCGTTCCATGTTGCCAGCG-------CGTAATGGCGGGGACTCATGGGAGACTGCCGGG 1136
              TTwist GTCCCGCAACGAGCGCAACCCTCGTCCTGTGTTGCCAGCG-------CGTAATGGCGGGGACTCACAGGAGACTGCCGGG 1145
           TTW08/27 GTCCCGCAACGAGCGCAACCCTCGTCCTGTGTTGCCAGCG-------CGTAATGGCGGGGACTCACAGGAGACTGCCGGG 1145
               Lxyli GTCCCGCAACGAGCGCAACCCTCGTTCTATGTTGCCAGCA-------CGTAATGGTGGGAACTCATAGGAGACTGCCGGG 1116
        Savermitilis GTCCCGCAACGAGCGCAACCCTTGTTCTGTGTTGCCAGCATGCCCTTCGGGGTGATGGGGACTCACAGGAGACCGCCGGG 1145
         Scoelicolor GTCCCGCAACGAGCGCAACCCTTGTCCCGTGTTGCCAGCAAGCCCTTCGGGGTGTTGGGGACTCACGGGAGACCGCCGGG 1148
         FalniACN14a GTCCCGCAACGAGCGCAACCCTCGTCCTATGTTGCCAGCG-------AGTTATGTCGGGGACTCATAGGAGACTGCCGGG 1131
               FCcI3 GTCCCGCAACGAGCGCAACCCTCGTCCTATGTTGCCAGCG-------AGTCGTGTCGGGGACTCATAGGAGACTGCCGGG 1131
            FEAN1pec GTCCCGCAACGAGCGCAACCCTTGTCCTATGTTGCCAGCG-------AGTCATGTCGGGGACTCATAGGAGACTGCCGGG 1129
      Acellulolyticus GTCCCGCAACGAGCGCAACCCTCGTCCCATGTTGCCAGCG-------GGTAATGCCGGGGACTCATGGGAGACTGCCGGG 1128
      MbovisAF2122/97 GTCCCGCAACGAGCGCAACCCTTGTCTCATGTTGCCAGCA-------CGTAATGGTGGGGACTCGTGAGAGACTGCCGGG 1157
  MtuberculosisH37Rv GTCCCGCAACGAGCGCAACCCTTGTCTCATGTTGCCAGCA-------CGTAATGGTGGGGACTCGTGAGAGACTGCCGGG 1157
        MbovisPasteur GTCCCGCAACGAGCGCAACCCTTGTCTCATGTTGCCAGCA-------CGTAATGGTGGGGACTCGTGAGAGACTGCCGGG 1157
MtuberculosisCDC1551 GTCCCGCAACGAGCGCAACCCTTGTCTCATGTTGCCAGCA-------CGTAATGGTGGGGACTCGTGAGAGACTGCCGGG 1156
           Mulcerans GTCCCGCAACGAGCGCAACCCTTGTCTCATGTTGCCAGCA-------CGTAATGGTGGGGACTCGTGAGAGACTGCCGGG 1155
              Mavium GTCCCGCAACGAGCGCAACCCTTGTCTCATGTTGCCAGCG-------GGTAATGCCGGGGACTCGTGAGAGACTGCCGGG 1143
          MaviumK-10 GTCCCGCAACGAGCGCAACCCTTGTCTCATGTTGCCAGCG-------GGTAATGCCGGGGACTCGTGAGAGACTGCCGGG 1155
           MlepraeTN GTCCCGCAACGAGCGCAACCCTTGTCTCATGTTGCCAGCA-------CGTAATGGTGGGGACTCGTGAGAGACTGCCGCG 1168
                MJLS GTCCCGCAACGAGCGCAACCCTTGTCTCATGTTGCCAGCA-------CGTTATGGTGGGGACTCGTGAGAGACTGCCGGG 1133
                MKMS GTCCCGCAACGAGCGCAACCCTTGTCTCATGTTGCCAGCA-------CGTTATGGTGGGGACTCGTGAGAGACTGCCGGG 1133
                MMCS GTCCCGCAACGAGCGCAACCCTTGTCTCATGTTGCCAGCA-------CGTTATGGTGGGGACTCGTGAGAGACTGCCGGG 1134
          Msmegmatis GTCCCGCAACGAGCGCAACCCTTGTCTCATGTTGCCAGCA-------CGTTATGGTGGGGACTCGTGAGAGACTGCCGGG 1146
        Mvanbaalenii GTCCCGCAACGAGCGCAACCCTTATCTTATGTTGCCAGCG-------CGTAATGGCGGGGACTCGTGAGAGACTGCCGGG 1133
          Nfarcinica GTCCCGCAACGAGCGCAACCCTTGTCCTGTGTTGCCAGCG-------CGTTATGGCGGGGACTCGCAGGAGACTGCCGGG 1141
               RRHA1 GTCCCGCAACGAGCGCAACCCTTGTCTTATGTTGCCAGCA-------CGTAATGGTGGGGACTCGTAAGAGACTGCCGGG 1139
        Cdiphtheriae GTCCCGCAACGAGCGCAACCCTTGTCTTATGTTGCCAGCA-------CGTGATGGTGGGGACTCATGAGAGACTGCCGGG 1112
           Cjeikeium GTCCCGCAACGAGCGCAACCCTTGTCTTGTGTTGCCAGCA-------CGTTATGGTGGGGACTCGCGAGAGACTGCCGGG 1149
          Cefficiens GTCCCGCAACGAGCGCAACCCTTGTCTTATGTTGCCATCA-------CGTGATGGTGGGCACTCATGAGAGACTGCCGGG 1123
          Cglutamicum GTCCCGCAACGAGCGCAACCCTTGTCTTATGTTGCCAGCA-------CATTGTGGTGGGTACTCATGAGAGACTGCCGGG 1143
         Nocardioides GTCCCGCAACGAGCGCAACCCTCGTCCTATGTTGCCAGCA-------CGTGATGGTGGGGACTCATAGGAGACTGCCGGG 1141
              Pacnes GTCCCGCAACGAGCGCAACCCTTGTTCACTGTTGCCAGCA-------CGTTATGGTGGGGACTCAGTGGAGACCGCCGGG 1140
            TfuscaYX GTCCCGCAACGAGCGCAACCCCTGTCCCATGTTGCCAGCA-------CGTGGTGGTGGGGACTCATGGGAGACTGCCGGG 1128
       Rxylanophilus GTCCCGCAACGAGCGCAACCCCCGCCCTGTGTTGCCAGCA---------TTGAGTTGGGGACTCACAGGGGACTGCCGGT 1144
               ruler ......1130......1140......1150......1160......1170......1180......1190......1200
```

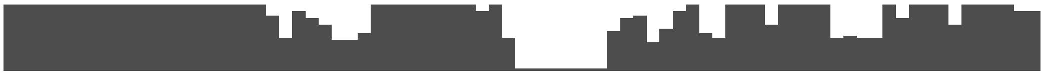

```
                     *  **  **************** *******  ******** ** *   * *  *****  *  * ***********
       Badolescentis GTCAACTCGGAGGAAGGTGGGGATGACGTCAGATCATCATGCCCCTTACGTCCAGGGCTTCACGCATGCTACAATGGCCG 1233
             Blongum GTTAACTCGGAGGAAGGTGGGGATGACGTCAGATCATCATGCCCCTTACGTCCAGGGCTTCACGCATGCTACAATGGCCG 1227
          Aaurescens GTCAACTCGGAGGAAGGTGGGGACGACGTCAAATCATCATGCCCCTTATGTCTTGGGCTTCACGCATGCTACAATGGCCG 1201
               AFB24 GTCAACTCGGAGGAAGGTGGGGACGACGTCAAATCATCATGCCCCTTATGTCTTGGGCTTCACGCATGCTACAATGGCCG 1216
              TTwist GTCAACTCGGAGGAAGGTGGGGATGACGTCAAATCATCATGCCCTTCG-GTCTTGGGCTTCACGCGTGCTACAATGGCTG 1224
            TTW08/27 GTCAACTCGGAGGAAGGTGGGGATGACGTCAAATCATCATGCCCTTCG-GTCTTGGGCTTCACGCGTGCTACAATGGCTG 1224
               Lxyli GTCAACTCGGAGGAAGGTGGGGATGACGTCAAATCATCATGCCCCTTATGTCTTGGGCTTCACGCATGCTACAATGGCCG 1196
        Savermitilis GTCAACTCGGAGGAAGGTGGGGACGACGTCAAGTCATCATGCCCCTTATGTCTTGGGCTGCACACGTGCTACAATGGCCG 1225
         Scoelicolor GTCAACTCGGAGGAAGGTGGGGACGACGTCAAGTCATCATGCCCCTTATGTCTTGGGCTGCACACGTGCTACAATGGCCG 1228
         FalniACN14a GTCAACTCGGAGGAAGGTGGGGATGACGTCAAGTCATCATGCCCCTTACGTCCTGGGCTGCACACATGCTACAATGGCCG 1211
               FCcI3 GTCAACTCGGAGGAAGGTGGGGATGACGTCAAGTCATCATGCCCCTTACGTCCTGGGCTGCACACATGCTACAATGGCCG 1211
            FEAN1pec GTCAACTCGGAGGAAGGTGGGGATGACGTCAAGTCATCATGCCCCTTACGTCCTGGGCTGCACACATGCTACAATGGCCG 1209
      Acellulolyticus GTCAACTCGGAGGAAGGTGGGGATGACGTCAAGTCATCATGCCCCTTACGTCCAGGGCTGCACACATGCTACAATGGCCG 1208
      MbovisAF2122/97 GTCAACTCGGAGGAAGGTGGGGATGACGTCAAGTCATCATGCCCCTTATGTCCAGGGCTTCACACATGCTACAATGGCCG 1237
  MtuberculosisH37Rv GTCAACTCGGAGGAAGGTGGGGATGACGTCAAGTCATCATGCCCCTTATGTCCAGGGCTTCACACATGCTACAATGGCCG 1237
       MbovisPasteur GTCAACTCGGAGGAAGGTGGGGATGACGTCAAGTCATCATGCCCCTTATGTCCAGGGCTTCACACATGCTACAATGGCCG 1237
MtuberculosisCDC1551 GTCAACTCGGAGGAAGGTGGGGATGACGTCAAGTCATCATGCCCCTTATGTCCAGGGCTTCACACATGCTACAATGGCCG 1236
           Mulcerans GTCAACTCGGAGGAAGGTGGGGATGACGTCAAGTCATCATGCCCCTTATGTCCAGGGCTTCACACATGCTACAATGGCCG 1235
              Mavium GTCAACTCGGAGGAAGGTGGGGATGACGTCAAGTCATCATGCCCCTTATGTCCAGGGCTTCACACATGCTACAATGGCCG 1223
          MaviumK-10 GTCAACTCGGAGGAAGGTGGGGATGACGTCAAGTCATCATGCCCCTTATGTCCAGGGCTTCACACATGCTACAATGGCCG 1235
           MlepraeTN GTCAACTCGGAGGAAGGTGGGGATGACGTCAAGTCATCATGCCCCTTATGTCCAGGGCTTCACACATGCTACAATGGCCG 1248
                MJLS GTCAACTCGGAGGAAGGTGGGGATGACGTCAAGTCATCATGCCCCTTATGTCCAGGGCTTCACACATGCTACAATGGCCG 1213
                MKMS GTCAACTCGGAGGAAGGTGGGGATGACGTCAAGTCATCATGCCCCTTATGTCCAGGGCTTCACACATGCTACAATGGCCG 1213
                MMCS GTCAACTCGGAGGAAGGTGGGGATGACGTCAAGTCATCATGCCCCTTATGTCCAGGGCTTCACACATGCTACAATGGCCG 1214
          Msmegmatis GTCAACTCGGAGGAAGGTGGGGATGACGTCAAGTCATCATGCCCCTTATGTCCAGGGCTTCACACATGCTACAATGGCCG 1226
        Mvanbaalenii GTCAACTCGGAGGAAGGTGGGGATGACGTCAAGTCATCATGCCCCTTATGTCCAGGGCTTCACACATGCTACAATGGCCG 1213
          Nfarcinica GTCAACTCGGAGGAAGGTGGGGACGACGTCAAGTCATCATGCCCCTTATGTCCAGGGCTTCACACATGCTACAATGGCCG 1221
               RRHA1 GTCAACTCGGAGGAAGGTGGGGACGACGTCAAGTCATCATGCCCCTTATGTCCAGGGCTTCACACATGCTACAATGGCCA 1219
        Cdiphtheriae GTTAACTCGGAGGAAGGTGGGGATGACGTCAAATCATCATGCCCCTTATGTCCAGGGCTTCACACATGCTACAATGGTCG 1192
           Cjeikeium GTTAACTCGGAGGAAGGTGGGGATGACGTCAAATCATCATGCCCCTTATGTCCAGGGCTTCACACATGCTACAATGGTCG 1229
          Cefficiens GTTAACTCGGAGGAAGGTGGGGATGACGTCAAATCATCATGCCCCTTATGTCCAGGGCTTCACACATGCTACAATGGTCG 1203
          Cglutamicum GTTAACTCGGAGGAAGGTGGGGATGACGTCAAATCATCATGCCCCTTATGTCCAGGGCTTCACACATGCTACAATGGTCG 1223
         Nocardioides GTCAACTCGGAGGAAGGTGGGGATGACGTCAAGTCATCATGCCCCTTATGTCCAGGGCTTCACGCATGCTACAATGGCCG 1221
              Pacnes GTCAACTCGGAGGAAGGTGGGGATGACGTCAAGTCATCATGCCCCTTATGTCCAGGGCTTCACGCATGCTACAATGGCTG 1220
            TfuscaYX GTCAACTCGGAGGAAGGTGGGGATGACGTCAAGTCATCATGCCCCTTATGCCTTGGGCTGCAAACATGCTACAATGGCCG 1208
       Rxylanophilus GACAAACCGGAGGAAGGTGGGGATGACGTCAAGTCATCATGGCCCTTATGCCCAGGGCTAGAAACGTGCTACAATGGCCG 1224
               ruler ......1210......1220......1230......1240......1250......1260......1270......1280
```

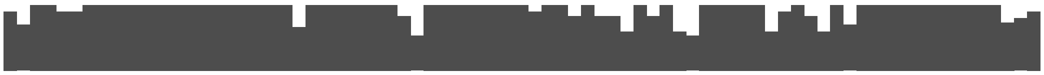

```
                      * **  * *   ** *    *  * *   ***  ***     **     * ** ********* *  ********* **
Badolescentis         GTACAACGGGA-TGCGACCTCGTGAGGGGGAGCGGATCCCTT-AAAACCGGTCTCAGTTCGGATTGGAGTCTGCAACCCG  1311
Blongum               GTACAACGGGA-TGCGACGCGGCGACGCGGAGCGGATCCCTG-AAAACCGGTCTCAGTTCGGATCGCAGTCTGCAACTCG  1305
Aaurescens            GTACAAAGGGT-TGCGATACTGTGAGGTGGAGCTAATCCCAA-AAAGCCGGTCTCAGTTCGGATTGGGGTCTGCAACTCG  1279
AFB24                 GTACAAAGGGT-TGCGATACTGTGAGGTGGAGCTAATCCCAA-AAAGCCGGTCTCAGTTCGGATTGGGGTCTGCAACTCG  1294
TTwist                GTACAGAGGGT-TGCAATATCGCAAGGTGGAGCGAATCTCAA-AAAGCCAGTCTCAGTTCGGATTGGGGTCTGCAACTCG  1302
TTW08/27              GTACAGAGGGT-TGCAATATCGCAAGGTGGAGCGAATCTCAA-AAAGCCAGTCTCAGTTCGGATTGGGGTCTGCAACTCG  1302
Lxyli                 GTACAAAGGGC-TGCAATACCGTAAGGTGGAGCGAATCCCAA-AAAGCCGGTCTCAGTTCGGATTGAGGTCTGCAACTCG  1274
Savermitilis          ATACAATGAGC-TGCGATACCGCAAGGTGGAGCGAATCTCAA-AAAGTCGGTCTCAGTTCGGATTGGGGTCTGCAACTCG  1303
Scoelicolor           GTACAATGAGC-TGCGATACCGCAAGGTGGAGCGAATCTCAA-AAAGCCGGTCTCAGTTCGGATTGGGGTCTGCAACTCG  1306
FalniACN14a           GTACAAAGGGC-TGCGATACCGTGAGGTGGAGCGAATCCCAA-AAAGCCGGTCTCAGTTCGGATCGGGGTCTGCAACTCG  1289
FCcI3                 GTACAAAGGGC-TGCGATGCCGTGAGGTGGAGCGAATCCCAA-AAAGCCGGTCTCAGTTCGGATCGGGGTCTGCAACTCG  1289
FEAN1pec              GTACAATGGGC-TGCGATACCGCGAGGTGGAGCGAATCCCAA-AAAGCCGGTCTCAGTTCGGATCGGGGTCTGCAACTCG  1287
Acellulolyticus       GTACAAAGGGC-TGCGAAACCGTGAGGTGGAGCGAATCCCAA-AAAGCCGGTCTCAGTTCGGATCGGGGTCTGCAACTCG  1286
MbovisAF2122/97       GTACAAAGGGC-TGCGATGCCGCGAGGTTAAGCGAATCCTTA-AAAGCCGGTCTCAGTTCGGATCGGGGTCTGCAACTCG  1315
MtuberculosisH37Rv    GTACAAAGGGC-TGCGATGCCGCGAGGTTAAGCGAATCCTTA-AAAGCCGGTCTCAGTTCGGATCGGGGTCTGCAACTCG  1315
MbovisPasteur         GTACAAAGGGC-TGCGATGCCGCGAGGTTAAGCGAATCCTTA-AAAGCCGGTCTCAGTTCGGATCGGGGTCTGCAACTCG  1315
MtuberculosisCDC1551  GTACAAAGGGC-TGCGATGCCGCGAGGTTAAGCGAATCCTTA-AAAGCCGGTCTCAGTTCGGATCGGGGTCTGCAACTCG  1314
Mulcerans             GTGCAAAGGGC-TGCGATGCCGCGAGGTTAAGCGAATCCTTT-AACGCCGGTCTCAGTTCGGATCGGGGTCTGCAACTCG  1313
Mavium                GTACAAAGGGC-TGCGATGCCGTAAGGTTAAGCGAATCCTTTTAAAGCCGGTCTCAGTTCGGATTGGGGTCTGCAACTCG  1302
MaviumK-10            GTACAAAGGGC-TGCGATGCCGTAAGGTTAAGCGAATCCTTTTAAAGCCGGTCTCAGTTCGGATTGGGGTCTGCAACTCG  1314
MlepraeTN             GTACAAAGGGC-TGCGATGCCGCAAGGTTAAGCGAATCCTTTTAAAGCCGGTCTCAGTTCGGATCGGGGTCTGCAACTCG  1327
MJLS                  GTACAAAGGGC-TGCGATGCCGTGAGGTGGAGCGAATCCTTTCAAAGCCGGTCTCAGTTCGGATCGGGGTCTGCAACTCG  1292
MKMS                  GTACAAAGGGC-TGCGATGCCGTGAGGTGGAGCGAATCCTTTCAAAGCCGGTCTCAGTTCGGATCGGGGTCTGCAACTCG  1292
MMCS                  GTACAAAGGGC-TGCGATGCCGTGAGGTGGAGCGAATCCTTTCAAAGCCGGTCTCAGTTCGGATCGGGGTCTGCAACTCG  1293
Msmegmatis            GTACAAAGGGC-TGCGATGCCGTGAGGTGGAGCGAATCCTTTCAAAGCCGGTCTCAGTTCGGATCGGGGTCTGCAACTCG  1305
Mvanbaalenii          GTACAAAGGGC-TGCGATGCCGTGAGGTGGAGCGAATCCTTTCAAAGCCGGTCTCAGTTCGGATCGGGGTCTGCAACTCG  1292
Nfarcinica            GTACAGAGGGC-TGCGATACCGTGAGGTGGAGCGAATCCCTT-AAAGCCGGTCTCAGTTCGGATCGGGGTCTGCAACTCG  1299
RRHA1                 GTACAGAGGGC-TGCGAGACCGTGAGGTGGAGCGAATCCCTT-AAAGCTGGTCTCAGTTCGGATCGGGGTCTGCAACTCG  1297
Cdiphtheriae          GTACAACGCGC-TGCGAGCCTGTGAGGGTGAGCGAATCGCTG-AAAGCCGGCCTCAGTTCGGATTGGGGTCTGCAACTCG  1270
Cjeikeium             GTACAGTGGGT-TGCGATACCGTGAGGTGGAGCTAATCCCTT-AAAGCCGGTCTCAGTTCGGATTGGAGTCTGCAACTCG  1307
Cefficiens            GTACAGCGAGT-TGCCACACCGTGAGGTGGAGCTAATCTCTC-AAAGCCGGCCTCAGTTCGGATTGGGGTCTGCAACTCG  1281
Cglutamicum           GTACAGCGAGT-TGCCACACCGTGAGGTGGAGCTAATCTCTT-AAAGCCGGCCTCAGTTCGGATTGGGGTCTGCAACTCG  1301
Nocardioides          GTACAAAGGGC-TGCGATGCTGTAAGGCGGAGCGAATCCCAA-AAAGCCGGTCTCAGTTCGGATTGGGGTCTGCAACTCG  1299
Pacnes                GTACAGAGAGT-GGCGAGCCTGTGAGGGTGAGCGAATCTCGG-AAAGCCGGTCTCAGTTCGGATTGGGGTCTGCAACTCG  1298
TfuscaYX              GTACAGAGGGCGTGCGATGTCGTGAGGCGGAGCGAATCTCTT-AAAGCCGGTCTCAGTTCGGATTGGGGTCTGCAACTCG  1287
Rxylanophilus         GTACAATGGGC-TGCGATGCCGCGAGGCGGAGCGAATCCCATCAAAGCCGGTCTAAGTTCGGATTGGAGTCTGCAACTCG  1303
ruler                 ......1290......1300......1310......1320......1330......1340......1350......1360
```

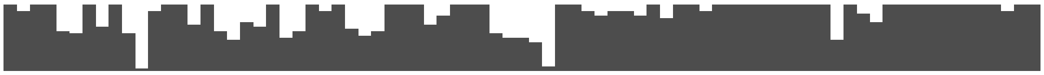

```
                      **  * *****  ***** ************  *******  *  ********* ********** **************
Badolescentis         ACTCCATGAAGGCGGAGTCGCTAGTAATCGCGGATCAGCAACGCCGCGGTGAATGCGTTCCCGGGCCTTGTACACACCGC  1391
Blongum               ACTGCGTGAAGGCGGAGTCGCTAGTAATCGCGAATCAGCAACGTCGCGGTGAATGCGTTCCCGGGCCTTGTACACACCGC  1385
Aaurescens            ACCCCATGAAGTCGGAGTCGCTAGTAATCGCAGATCAGCAACGCTGCGGTGAATACGTTCCCGGGCCTTGTACACACCGC  1359
AFB24                 ACCCCATGAAGTCGGAGTCGCTAGTAATCGCAGATCAGCAACGCTGCGGTGAATACGTTCCCGGGCCTTGTACACACCGC  1374
TTwist                ACCCCATGAAGTCGGAGTCGCTAGTAATCGCAGATCAGCAATGCTGCGGTGAATACGTTCCCGGGCCTTGTACACACCGC  1382
TTW08/27              ACCCCATGAAGTCGGAGTCGCTAGTAATCGCAGATCAGCAATGCTGCGGTGAATACGTTCCCGGGCCTTGTACACACCGC  1382
Lxyli                 ACCTCATGAAGTCGGAGTCGCTAGTAATCGCAGATCAGCAACGCTGCGGTGAATACGTTCCCGGGCCTTGTACACACCGC  1354
Savermitilis          ACCCCATGAAGTTGGAGTTGCTAGTAATCGCAGATCAGCATTGCTGCGGTGAATACGTTCCCGGGCCTTGTACACACCGC  1383
Scoelicolor           ACCCCATGAAGTCGGAGTCGCTAGTAATCGCAGATCAGCATTGCTGCGGTGAATACGTTCCCGGGCCTTGTACACACCGC  1386
FalniACN14a           ACCCCGTGAAGTCGGAGTCGCTAGTAATCGCAGATCAGCAATGCTGCGGTGAATACGTTCCCGGGCCTTGTACACACCGC  1369
FCcI3                 ACCCCGTGAAGTCGGAGTCGCTAGTAATCGCAGATCAGCAATGCTGCGGTGAATACGTTCCCGGGCCTTGTACACACCGC  1369
FEAN1pec              ACCCCGTGAAGTCGGAGTCGCTAGTAATCGCAGATCAGCAATGCTGCGGTGAATACGTTCCCGGGCCTTGTACACACCGC  1367
Acellulolyticus       ACCCCGTGAAGTCGGAGTCGCTAGTAATCGCGGATCAGCAATGCCGCGGTGAATACGTTCCCGGGCCTTGTACACACCGC  1366
MbovisAF2122/97       ACCCCGTGAAGTCGGAGTCGCTAGTAATCGCAGATCAGCAACGCTGCGGTGAATACGTTCCCGGGCCTTGTACACACCGC  1395
MtuberculosisH37Rv    ACCCCGTGAAGTCGGAGTCGCTAGTAATCGCAGATCAGCAACGCTGCGGTGAATACGTTCCCGGGCCTTGTACACACCGC  1395
MbovisPasteur         ACCCCGTGAAGTCGGAGTCGCTAGTAATCGCAGATCAGCAACGCTGCGGTGAATACGTTCCCGGGCCTTGTACACACCGC  1395
MtuberculosisCDC1551  ACCCCGTGAAGTCGGAGTCGCTAGTAATCGCAGATCAGCAACGCTGCGGTGAATACGTTCCCGGGCCTTGTACACACCGC  1394
Mulcerans             ACCCCGTGAAGTCGGAGTCGCTAGTAATCGCAGATCAGCAACGCTGCGGTGAATACGTTCCCGGGCCTTGTACACACCGC  1393
Mavium                ACCCCATGAAGTCGGAGTCGCTAGTAATCGCAGATCAGCAACGCTGCGGTGAATACGTTCCCGGGCCTTGTACACACCGC  1382
MaviumK-10            ACCCCATGAAGTCGGAGTCGCTAGTAATCGCAGATCAGCAACGCTGCGGTGAATACGTTCCCGGGCCTTGTACACACCGC  1394
MlepraeTN             ACCCCGTGAAGTCGGAGTCGCTAGTAATCGCAGATCAGCAACGCTGCGGTGAATACGTTCCCGGGCCTTGTACACACCGC  1407
MJLS                  ACCCCGTGAAGTCGGAGTCGCTAGTAATCGCAGATCAGCAACGCTGCGGTGAATACGTTCCCGGGCCTTGTACACACCGC  1372
MKMS                  ACCCCGTGAAGTCGGAGTCGCTAGTAATCGCAGATCAGCAACGCTGCGGTGAATACGTTCCCGGGCCTTGTACACACCGC  1372
MMCS                  ACCCCGTGAAGTCGGAGTCGCTAGTAATCGCAGATCAGCAACGCTGCGGTGAATACGTTCCCGGGCCTTGTACACACCGC  1373
Msmegmatis            ACCCCGTGAAGTCGGAGTCGCTAGTAATCGCAGATCAGCAACGCTGCGGTGAATACGTTCCCGGGCCTTGTACACACCGC  1385
Mvanbaalenii          ACCCCGTGAAGTCGGAGTCGCTAGTAATCGCAGATCAGCAACGCTGCGGTGAATACGTTCCCGGGCCTTGTACACACCGC  1372
Nfarcinica            ACCCCGTGAAGTTGGAGTCGCTAGTAATCGCAGATCAGCAACGCTGCGGTGAATACGTTCCCGGGCCTTGTACACACCGC  1379
RRHA1                 ACCCCGTGAAGTCGGAGTCGCTAGTAATCGCAGATCAGCAACGCTGCGGTGAATACGTTCCCGGGCCTTGTACACACCGC  1377
Cdiphtheriae          ACCCCATGAAGTCGGAGTCGCTAGTAATCGCAGATCAGCAACGCTGCGGTGAATACGTTCCCGGGCCTTGTACACACCGC  1350
Cjeikeium             ACTCCATGAAGTCGGAGTCGCTAGTAATCGCAGATCAGCAACGCTGCGGTGAATACGTTCCCGGGCCTTGTACACACCGC  1387
Cefficiens            ACCCCATGAAGTCGGAGTCGCTAGTAATCGCAGATCAGCAACGCTGCGGTGAATACGTTCCCGGGCCTTGTACACACCGC  1361
Cglutamicum           ACCCCATGAAGTCGGAGTCGCTAGTAATCGCAGATCAGCAACGCTGCGGTGAATACGTTCCCGGGCCTTGTACACACCGC  1381
Nocardioides          ACCCCATGAAGTCGGAGTCGCTAGTAATCGCAGATCAGCAACGCTGCGGTGAATACGTTCCCGGGCCTTGTACACACCGC  1379
Pacnes                ACCTCATGAAGTCGGAGTCGCTAGTAATCGCAGATCAGCAACGCTGCGGTGAATACGTTCCCGGGCCTTGTACACACCGC  1378
TfuscaYX              ACCCCATGAAGGTGGAGTCGCTAGTAATCGCGGATCAGCAACGCCGCGGTGAATACGTTCCCGGGCCTTGTACACACCGC  1367
Rxylanophilus         ACTCCATGAAGGCGGAGTTGCTAGTAATCGCGGATCAGCA-TGCCGCGGTGAATACGTTCCCGGGCCTTGTACACACCGC  1382
ruler                 ......1370......1380......1390......1400......1410......1420......1430......1440
```

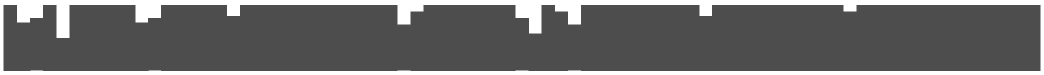

```
                       *****    ** *  **  ** *  ******* *  * *
Badolescentis          CCGTCAAGTCATGAAAGTGGGTAGCACCCGAAGCCGGTG-GCCCAACCTTTTTG-GGG--GGAGCCGTC----TAAGGTG  1463
Blongum                CCGTCAAGTCATGAAAGTGGGCAGCACCCGAAGCCGGTG-GCCTAACCCCTTGT-GGGATGGAGCCGTC----TAAGGTG  1459
Aaurescens             CCGTCAAGTCACGAAAGTTGGTAACACCCGAAGCCGGTG-GCCTAACCC-TTGT-GGG-GGGAGCCGTC----GAAGGTG  1431
AFB24                  CCGTCAAGTCACGAAAGTTGGTAACACCCGAAGCCGGTG-GCCTAACCCCTTGT-GGGAGGGAGCTGTC----GAAGGTG  1448
TTwist                 CCGTCAAGTCATGAAAGCTGGTAACACCCGAAGCCGGTG-ACTTAACCT-TTTT--GGAGAGAGCCGTC----GAAGGTG  1454
TTW08/27               CCGTCAAGTCATGAAAGCTGGTAACACCCGAAGCCGGTG-ACTTAACCT-TTTT-TGGAGAGAGCCGTC----GAAGGTG  1455
Lxyli                  CCGTCAAGTCATGAAAGTCGGTAACACCCGAAGCCGGTG-GCCCAACCC-TTGT--GGAGGGAGCCGTC----GAAGGTG  1426
Savermitilis           CCGTCACGTCACGAAAGTCGGTAACACCCGAAGCCGGTG-GCCCAACCCCTTGT-GGGAGGGAGCTGTC----GAAGGTG  1457
Scoelicolor            CCGTCACGTCACGAAAGTCGGTAACACCCGAAGCCGGTG-GCCCAACCCCTTGT-GGGAGGGAGCTGTC----GAAGGTG  1460
FalniACN14a            CCGTCACGTCACGAAAGTCGGTAACACCCGAAGCCGGTG-GCCTAACCCTTGT--GGG-GGGAGCCGTC----GAAGGTG  1441
FCcI3                  CCGTCACGTCACGAAAGTCGGTAACACCCGAAGCCGGTG-GCCTAACCCTTGT--GGG-GGGAGCCGTC----GAAGGTG  1441
FEAN1pec               CCGTCACGTCACGAAAGTCGGTAACACCCGAAGCCGGTG-GCCCAACCCTTGT--GGG-GGGAGCCGTC----GAAGGTG  1439
Acellulolyticus        CCGTCACGTCACGAAAGTCGGTAACACCCGAAGCCGGTG-GCCCAACCCGCAA--GGGAGGGAGCCGTC----GAAGGTG  1439
MbovisAF2122/97        CCGTCACGTCATGAAAGTCGGTAACACCCGAAGCCAGTG-GCCTAACCCTC----GGGAGGGAGCTGTC----GAAGGTG  1466
MtuberculosisH37Rv     CCGTCACGTCATGAAAGTCGGTAACACCCGAAGCCAGTG-GCCTAACCCTC----GGGAGGGAGCTGTC----GAAGGTG  1466
MbovisPasteur          CCGTCACGTCATGAAAGTCGGTAACACCCGAAGCCAGTG-GCCTAACCCTC----GGGAGGGAGCTGTC----GAAGGTG  1466
MtuberculosisCDC1551   CCGTCACGTCATGAAAGTCGGTAACACCCGAAGCCAGTG-GCCTAACCCTC----GGGAGGGAGCTGTC----GAAGGTG  1465
Mulcerans              CCGTCACGTCATGAAAGTCGGTAACACCCGAAGCCAGTG-GCCTAACCCTTTTTTGGGAGGGAGCTGTC----GAAGGTG  1468
Mavium                 CCGTCACGTCATGAAAGTCGGTAACACCCGAAGCCAGTG-GCCTAACCCTTTT--GGGAGGGAGCTGTC----GAAGGTG  1455
MaviumK-10             CCGTCACGTCATGAAAGTCGGTAACACCCGAAGCCAGTG-GCCTAACCCTTTT--GGGAGGGAGCTGTC----GAAGGTG  1467
MlepraeTN              CCGTCACGTCATGAAAGTCGGTAACACCCGAAGCCAGTG-GCCTAACCCTC----GGGAGGGAGCTGTC----CAAGGTG  1478
MJLS                   CCGTCACGTCATGAAAGTCGGTAACACCCGAAGCCGGTG-GCCTAACCCCTTGT-GGGAGGGAGCCGTC----GAAGGTG  1446
MKMS                   CCGTCACGTCATGAAAGTCGGTAACACCCGAAGCCGGTG-GCCTAACCCCTTGT-GGGAGGGAGCCGTC----GAAGGTG  1446
MMCS                   CCGTCACGTCATGAAAGTCGGTAACACCCGAAGCCGGTG-GCCTAACCCCTTGT-GGGAGGGAGCCGTC----GAAGGTG  1447
Msmegmatis             CCGTCACGTCATGAAAGTCGGTAACACCCGAAGCCGGTG-GCCTAACCC-TTGT-GG-AGGGAGCCGTC----GAAGGTG  1457
Mvanbaalenii           CCGTCACGTCATGAAAGTCGGTAACACCCGAAGCCGGTG-GCCTAACCCCTTGT-GGGAGGGAGCCGTC----GAAGGTG  1446
Nfarcinica             CCGTCACGTCATGAAAGTCGGTAACACCCGAAGCCGGTG-GCCTAACCCCTTGT-GGGAGGGAGCCGTC----GAAGGTG  1453
RRHA1                  CCGTCACGTCATGAAAGTCGGTAACACCCGAAGCCGGTG-GCCTAACCCCTTGT-GGGAGGGAGCCGTC----GAAGGTG  1451
Cdiphtheriae           CCGTCACGTCATGAAAGTTGGTAACACCCGAAGCCAGTG-GCC-------------------------------------  1392
Cjeikeium              CCGTCGCGTCATGAAAGTTGGTAACACCCGAAGCCAGTG-GCCCAAACTTGT---TAGGG--AGCTGTC----GAAGGTG  1457
Cefficiens             CCGTCACGTCATGAAAGTTGGTAACACCCGAAGCCAGTG-GCCCAACC--------------------------------  1408
Cglutamicum            CCGTCACGTCATGAAAGTTGGTAACACCCGAAGCCAGTG-GCCCAACCTTTT---AGGGGGGAGCTGTC----GAAGGTG  1453
Nocardioides           CCGTCACGTCACGAAAGTCGGCAACACCCGAAGCCGGTG-GCCTAACCCTTG---TGGGGGGAGCCGTC----GAAGGTG  1451
Pacnes                 CCGTCAAGTCATGAAAGTTGGTAACACCCGAAGCCGGTG-GCCTAACCGTTG---TGGGGG-AGCCGTC----GAAGGTG  1449
TfuscaYX               CCGTCACGTCACGAAAGTCGGCAACACCCGAAACACGTG-GCCTAACCCTTTGT-GGGGGGGAGCGTGT----GAAGGTG  1441
Rxylanophilus          CCGTCACACCACGGGAGTCGGCAATACCCGAAGCAGGCGCAGCTAACCCCTCTTCGGAGGGGAGGCGGCTTGCGAAGGTA  1462
ruler                  ......1450......1460......1470......1480......1490......1500......1510......1520
```

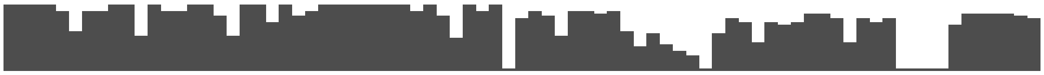

```
Badolescentis          AGACTCGTGATTGGGACTAAGTCGTAACAAGGTAGCCGTACCGGAAGGTGCGGCTGGATCACCTCCTTTCT------  1534
Blongum                AGGCTCGTGATTGGGACTAAGTCGTAACAAGGTAGCCGTACCGGAAGGTGCGGCTGGATCACCTCCTTTCT------  1530
Aaurescens             GGACCGGCGATTGGGACTAAGTCGTAACAAGGTAGCCGTACCGGAAGGTG---------------------------  1481
AFB24                  GGACTGGCGATTGGGACTAAGTCGTAACAAGGTAGCCGTACCGGAAGGTGCGGCTGGATCACCTCCTTT--------  1517
TTwist                 GGATTGGTGATTGGGACTAAGTCGTAACAAGGTAGCCGTACCGGAAGGTGCGGCTGGATCACCTCCTTTCT------  1525
TTW08/27               GGATTGGTGATTGGGACTAAGTCGTAACAAGGTAGCCGTACCGGAAGGTGCGGCTGGATCACCTCCTTTCT------  1526
Lxyli                  GGGTCGGTGATTAGGACTAAG--------------------------------------------------------  1447
Savermitilis           GGACTGGCGATTGGGACGAAGTCGTAACAAGGTAGCCGTACCGGAAGGTGCGGCTGGATCACCTCCTTTCTAA----  1530
Scoelicolor            GGACTGGCGATTGGGACGAAGTCGTAACAAGGTAGCCGTACCGGAAGGTGCGGCTGGATCACCTCCTTTCT------  1531
FalniACN14a            GGACCGGCGATTGGGACGAAGTCGTAACAAGGTAGCCGTACCGGAAGGTGCGGCTGGATCACCTCCTTT--------  1510
FCcI3                  GGACCGGCGATTGGGACGAAGTCGTAACAAGGTAGCCGTACCGGAAGGTGCGGCTGGATCACCTCCTTTCT------  1512
FEAN1pec               GGACCGGCGATTGGGACGAAGTCGTAACAAGGTAGCCGTACCGGAAGGTGCGGCTGGATCACCTCCTTT--------  1508
Acellulolyticus        GGACCGGCGATTGGGACGAAGTCGTAACAAGGTAGCCGTACCGGAAGGTGTGGCTGGATCACCTCCTTTC-------  1509
MbovisAF2122/97        GGATCGGCGATTGGGACGAAGTCGTAACAAGGTAGCCGTACCGGAAGGTGCGGCTGGATCACCTCCTTTCT------  1537
MtuberculosisH37Rv     GGATCGGCGATTGGGACGAAGTCGTAACAAGGTAGCCGTACCGGAAGGTGCGGCTGGATCACCTCCTTTCT------  1537
MbovisPasteur          GGATCGGCGATTGGGACGAAGTCGTAACAAGGTAGCCGTACCGGAAGGTGCGGCTGGATCACCTCCTTTCT------  1537
MtuberculosisCDC1551   GGATCGGCGATTGGGACGAAGTCGTAACAAGGTAGCCGTACCGGAAGGTGCGGCTGGATCACCTCCTTTCT------  1536
Mulcerans              GGATCGGCGATTGGGACGAAGTCGTAACAAGGTAGCCGTACCGGAAGGTGCGGCTGGATCACCTCCTTTCT------  1539
Mavium                 GGATCGGCGATTGGGACGAAGTCGTAACAAGGTAGCCGTACCGGAAGGTGCGGCTGGATCAC--------------  1517
MaviumK-10             GGATCGGCGATTGGGACGAAGTCGTAACAAGGTAGCCGTACCGGAAGGTGCGGCTGGATCACCTCCTTTCTAAGGAG  1544
MlepraeTN              GGATCGGCGATTGGGACGAAGTCGTAACAAGGTAGCCGTACCGGAAGGTGCGGCTGGATCACCTCCTTTCT------  1549
MJLS                   GGATCGGCGATTGGGACGAAGTCGTAACAAGGTAGCCGTACCGGAAGGTGCGGCTGGATCACCTCCTTT--------  1515
MKMS                   GGATCGGCGATTGGGACGAAGTCGTAACAAGGTAGCCGTACCGGAAGGTGCGGCTGGATCACCTCCTTT--------  1515
MMCS                   GGATCGGCGATTGGGACGAAGTCGTAACAAGGTAGCCGTACCGGAAGGTGCGGCTGGATCACCTCCTT---------  1515
Msmegmatis             GGATCGGCGATTGGGACGAAGTCGTAACAAGGTAGCCGTACCGGAAGGTGCGGCTGGATCACCTCCTTTCT------  1528
Mvanbaalenii           GGATCGGCGATTGGGACGAAGTCGTAACAAGGTAGCCGTACCGGAAGGTGCGGCTGGATCACCTCCTTT--------  1515
Nfarcinica             GGATCGGCGATTGGGACGAAGTCGTAACAAGGTAGCCGTACCGGAAGGTGCGGCTGGATCACCTCCTTTCTAAG---  1527
RRHA1                  GGATCGGCGATTGGGACGAAGTCGTAACAAGGTAGCCGTACCGGAAGGTGCGGCTGGATCACCTCCTT---------  1519
Cdiphtheriae           -----------------------------------------------------------------------------  1392
Cjeikeium              GGATTGGCGATTGGGACGAAGTCGTAACAAGGTAGCCGTACCGGAAGGTGCGGCTGGATCACCTCCTTTC-------  1527
Cefficiens             -----------------------------------------------------------------------------  1408
Cglutamicum            GGATCGGCGATTGGGACGAAGTCGTAACAAGGTAGCCGTACCGGAAGGTGCGGCTGGATCACCTCCTTTCT------  1524
Nocardioides           GGGCTGGCGATTGGGACGAAGTCGTAACAAGGTAGCCGTACCGGAAGGTGCGGCTGGATCACCTCCTTT--------  1520
Pacnes                 GGACTGGTGATTAGGACTAAGTCGTAACAAGGTAGCCGTACCGGAAGGTGCGGCTGGATCACCTCCTTTCTAAGGA-  1525
TfuscaYX               GGGCTGGCGATTGGGACGAAGTCGTAACAA-----------------------------------------------  1471
Rxylanophilus          GGGCCGGCGACTGGGGTGAAGTCGTAACAAGGTAGCCGTACCTGAAGGTGC--------------------------  1513
ruler                  ......1530......1540......1550......1560......1570......1580......1590.......
```

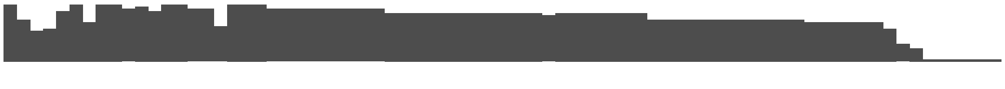

Supplement: Additional File 6 — Multiple alignment of the 16S sequences. [file 1471-2148-8-185-S6.pdf]
